# Dietary biomarkers—an update on their validity and applicability in epidemiological studies

**DOI:** 10.1093/nutrit/nuad119

**Published:** 2023-10-03

**Authors:** Rikard Landberg, Prasoona Karra, Rachel Hoobler, Erikka Loftfield, Inge Huybrechts, Jodi I Rattner, Stefania Noerman, Liesel Claeys, Vanessa Neveu, Nanna Hjort Vidkjaer, Otto Savolainen, Mary C Playdon, Augustin Scalbert

**Affiliations:** Division of Food and Nutrition Science, Department of Life Sciences, Chalmers University of Technology, Gothenburg, Sweden; Department of Nutrition and Integrative Physiology, University of Utah, Salt Lake City, UT, USA; Cancer Control and Population Sciences Program, Huntsman Cancer Institute, University of Utah Salt Lake City, UT, USA; Department of Nutrition and Integrative Physiology, University of Utah, Salt Lake City, UT, USA; Cancer Control and Population Sciences Program, Huntsman Cancer Institute, University of Utah Salt Lake City, UT, USA; Division of Cancer Epidemiology and Genetics, National Cancer Institute, Bethesda, MD, USA; International Agency for Research on Cancer, Nutrition and Metabolism Branch, Lyon, France; International Agency for Research on Cancer, Nutrition and Metabolism Branch, Lyon, France; Division of Food and Nutrition Science, Department of Life Sciences, Chalmers University of Technology, Gothenburg, Sweden; International Agency for Research on Cancer, Molecular Mechanisms and Biomarkers Group, Lyon, France; International Agency for Research on Cancer, Nutrition and Metabolism Branch, Lyon, France; Division of Food and Nutrition Science, Department of Life Sciences, Chalmers University of Technology, Gothenburg, Sweden; Division of Food and Nutrition Science, Department of Life Sciences, Chalmers University of Technology, Gothenburg, Sweden; Department of Nutrition and Integrative Physiology, University of Utah, Salt Lake City, UT, USA; Cancer Control and Population Sciences Program, Huntsman Cancer Institute, University of Utah Salt Lake City, UT, USA; International Agency for Research on Cancer, Nutrition and Metabolism Branch, Lyon, France

**Keywords:** blood, cohort, dietary biomarkers, epidemiology, food component biomarkers, food intake biomarkers, urine, validation

## Abstract

The aim of this literature review was to identify and provide a summary update on the validity and applicability of the most promising dietary biomarkers reflecting the intake of important foods in the Western diet for application in epidemiological studies. Many dietary biomarker candidates, reflecting intake of common foods and their specific constituents, have been discovered from intervention and observational studies in humans, but few have been validated. The literature search was targeted for biomarker candidates previously reported to reflect intakes of specific food groups or components that are of major importance in health and disease. Their validity was evaluated according to 8 predefined validation criteria and adapted to epidemiological studies; we summarized the findings and listed the most promising food intake biomarkers based on the evaluation. Biomarker candidates for alcohol, cereals, coffee, dairy, fats and oils, fruits, legumes, meat, seafood, sugar, tea, and vegetables were identified. Top candidates for all categories are specific to certain foods, have defined parent compounds, and their concentrations are unaffected by nonfood determinants. The correlations of candidate dietary biomarkers with habitual food intake were moderate to strong and their reproducibility over time ranged from low to high. For many biomarker candidates, critical information regarding dose response, correlation with habitual food intake, and reproducibility over time is yet unknown. The nutritional epidemiology field will benefit from the development of novel methods to combine single biomarkers to generate biomarker panels in combination with self-reported data. The most promising dietary biomarker candidates that reflect commonly consumed foods and food components for application in epidemiological studies were identified, and research required for their full validation was summarized.

## INTRODUCTION

Diet is an important modifiable risk factor for noncommunicable diseases, including cardiovascular disease, type 2 diabetes, and certain cancers.^1^ Evidence of dietary relationships with disease largely stems from observational studies, where self-reporting tools like food-frequency questionnaires (FFQs), 24-hour recalls (24-HRs), and weighed food records (FRs) have been used to estimate food intake.^2^^,^^3^ Yet, large random and systematic measurement errors hamper the accuracy of these tools to capture long-term food intake, although methods have been developed to tackle measurement errors, such as combining multiple 24-HRs.^4^^,^^5^ There is a need for new tools and methods to reflect long-term dietary exposures objectively and more accurately.^5^^,^^6^ Dietary biomarkers are promising instruments for objective dietary assessment, as they are molecules (molecular weight <1000 Da) derived from specific foods that are absorbed and detected in biological samples from humans in response to food intake, and they do not depend on participant recall, motivation, or behavior.^7^ Dietary biomarkers vary in their definition and applications. Recovery biomarkers provide a quantitative measure of food intake, as their excretion corresponds to the intake amount and can thus be used to correct for dietary measurement error. Concentration biomarkers correlate with food intake and can rank individuals with respect to food intake, although metabolism and other characteristics may affect their measured level. Replacement and prediction biomarkers are highly predictive of food intake, but they do not fulfill the requirements of recovery biomarkers.^6^^,^^7^ More recently, other classification schemes have emerged to account for other applications and features,^5^^,^^8^ including classifying exposure biomarkers into biomarkers of food component intake, food intake, and dietary patterns,^8^ either as single biomarkers or multiple-biomarker panels. Few valid and reliable food-related biomarkers exist at present, but recent developments in high-resolution mass spectrometry (MS), and to some extent nuclear magnetic resonance spectroscopy (NMR)–based techniques, have increasingly been used for diet-related biomarker discovery, validation, and implementation.^9^^,^^10^ Large databases now facilitate research and development of biomarker validation and implementation.^11–13^ Both controlled-feeding studies and large-scale epidemiological studies that leverage metabolomics have discovered novel biomarkers for a wide diversity of foods, food groups, and dietary patterns.^14^^,^^15^ Carefully controlled intervention studies are particularly useful to assess the pharmacokinetics of biomarker candidates as well as to establish dose response. Observational studies are useful to characterize biomarker variability under free-living conditions, and to estimate long-term biomarker stability. In some cases, analyses of putative dietary biomarkers in relation to health outcomes have complemented those using self-reported dietary data.^16–21^ However, few candidate biomarkers meet all proposed criteria for validation, often because methodological studies are lacking. Although standardized validation schemes are scarce,^22^ typical validation criteria include the following: biomarker assay validation, biomarker kinetics (half-life), assessment of the correlation of the biomarker with true food intake (using surrogate measures), dose response, specificity and sensitivity, exploration of nonfood intake–related determinants, and between-person and within-person variation over time.^7^^,^^23–25^ Other criteria include robustness across studies of different designs.^15^^,^^17^^,^^26^

The aim of this review was to identify and provide a summary update on the validity and applicability of the most promising dietary biomarkers reflecting important foods in the Western diet that can be applied in epidemiological studies. Each biomarker was evaluated against the recently developed 8-step validation process for diet-related biomarkers that systematically assesses candidate biomarker plausibility, dose response, time response, robustness, reliability, stability, analytical performance, and interlaboratory reproducibility.^25^ This scheme was extended by evaluating data on biomarker reproducibility over time, and knowledge gaps and opportunities for future research are highlighted.

## METHODS

### Selected food items

The biomarker candidates searched for were previously reported to reflect intake of specific food groups or components that are of major importance in health and disease, or that are included in official dietary guidelines and by nongovernmental organizations such as the World Cancer Research Fund/American Institute for Cancer Research.^27^ The Food Biomarker Alliance (FoodBAll; https://www.wur.nl/en/project/foodball.htm) extensively reviewed putative dietary biomarkers,^25^ which served as the foundation for the current review. The literature on biomarkers of the following foods or food components was reviewed: alcohol, cereals, coffee, dairy, fats and oils, fish, fruits, legumes, meat, seafood, sugar, tea, and vegetables. For cereals, whole-grain wheat, rye, oats, as well as bran from wheat, rye, and sourdough fermented bread (rye) were included. Whole-grain and refined-grain rice, maize, millet, sorghum, barley, and their products were excluded given the lack of promising, specific candidate biomarkers for them.^26^ Among other plant foods, total fruit intake, total vegetable intake, specific fruits and vegetables, and legumes were reviewed. Intake biomarkers of processed meat, red meat, poultry,^28^^,^^29^ total fish, lean fish, and other seafood^29^^,^^30^ were evaluated. Putative dairy intake biomarkers included biomarkers of total dairy, milk, yogurt, and cheese products.^31–34^ Biomarkers of commonly consumed beverages like tea, coffee, and alcohol were also evaluated.^35^ Among sugars, fructose and sucrose^36^ were included in the review based on their extensive consumption.^37^ Additionally, candidate biomarkers^38^ of fat and oil intakes, as well for which the typical biomarkers were primarily determined by the fat or oil food source, were reviewed (eg, fish oil vs plant oils, etc).^36^

### Biomarker validation criteria

In this review, a modified version of the systematic framework for food intake biomarker validation defined and reported by the FoodBAll consortium^25^ was used. The review focuses on biomarkers of habitual food intake in population studies and does not include biomarkers of compliance in short-term dietary intervention studies. In the FoodBAll consortium framework, 8 validation criteria that apply to different study designs include the following: plausibility (chemical/biological plausibility and specificity), dose response (across different levels of intake), time response (biomarker kinetics), robustness (reflection of a specific food in a whole-meal/diet context), reliability (comparable with other biomarkers or dietary instruments used to reflect the same food), stability (chemically and biologically), analytical performance (accuracy of the assay), and interlaboratory reproducibility (similar results across at least 2 laboratories).^25^ In the modified criteria applicable to epidemiological studies (Table 1), the plausibility, dose–response, and time–response criteria from those defined by Dragsted et al^25^ were included. Instead of robustness, a specific criterion for “correlation with habitual food intake” and “correlation with short-term food intake” was used, which addresses the correlation with intake under free-living conditions but does not formally consider that the biomarker must have been validated in controlled dietary intervention studies. The magnitude of correlation between human specimen–derived biomarkers and dietary intakes estimated by FFQs, 24-HRs, or FRs is represented by the correlation coefficient “*r*”. Correlations with *r* < 0.2 were considered “weak,” “moderate” when *r* = 0.2–0.5, and “strong” when *r* > 0.5.^39^ The stability criteria were excluded because biomarkers used from free-living studies typically rely on samples from biobanks and cohorts that have been stored over a longer time period and chemical stability tests of such storage conditions are lacking for most biomarkers. The analytical performance criteria were simplified to indicate information regarding “biospecimen” and “analytical method” used to measure the biomarker. Finally, the interlaboratory reproducibility criterion was excluded because these data were largely unavailable. In addition, “reproducibility over time” was included, mainly represented by the intraclass correlation coefficient (ICC) of repeated measures over time to provide a measure of how well the long-term biomarker concentration could be reflected in a single measurement. Most cohorts provide biospecimens measured at a single time point and the candidate biomarkers are typically measured against habitual dietary intake during the prior 12 months. Reproducibility over time was considered as “poor” when ICC < 0.4, “fair” when ICC = 0.4–0.6, “good” when ICC = 0.60–0.75, and excellent when ICC > 0.75.^40^

**Table 1:** Candidate dietary biomarker validation criteria

Candidate dietary biomarker validation criteria	Description
Nature of the biomarker and its specificity/plausibility	Is the biomarker a parent compound or a metabolite derived from intake/metabolism of the diet exposure? To what extent is the biomarker specific for the food?
Biospecimen	Is the biomarker present in plasma, urine, or other matrices (such as adipose tissue, nails, hair)?
Analytical method	What analytical methods were used to analyze the biomarker (LC, GC, NMR or other)?
Correlation with habitual food intake	What is the magnitude of correlation of the biomarker with habitual food intake assessed by FFQ?
Time response	What is the temporal relationship of the biomarker with food intake assessed by pharmacokinetics parameters (ie, elimination half-life)?
Reproducibility over time	What is the ratio of between-subject variation to the sum of between- and within-subject variation (ICC)? How does this relate to half-life and frequency of intake?
Dose response	What is the biomarker concentration following sequential increases in food intake under controlled or free-living conditions?

*Abbreviations*: FFQ, food-frequency questionnaire; FR, food record; GC, gas chromatography; ICC, intraclass correlation coefficient; LC, liquid chromatography; NMR, nuclear magnetic resonance spectroscopy; 24-HR, 24-hour dietary recall.

### Selection of studies

Dietary candidate biomarkers have typically emerged from small short-term human feeding trials or cross-sectional population-based studies.^5^ To evaluate the different elements of dietary biomarker validity as outlined above, data primarily from cross-sectional studies nested within prospective cohort studies were included. Assessment of dose response used information from dietary intervention studies. Animal studies were not reviewed.

### Search strategy and biomarker evaluation process

In this paper, the search strategies reported in the recent review articles on food intake biomarkers derived from the FoodBAll consortium^26^ were replicated and extended. For each dietary exposure, we present a summary of candidate dietary biomarkers and validation criteria assessment (see Table S1 in the Supporting Information online). For each dietary exposure, standardized summary sheets of top biomarkers were compiled with appraisal of validation criteria along with key references (see Text S1 in the Supporting Information online).

## RESULTS AND DISCUSSION

Table S1 in the Supporting Information online summarizes the results from a literature review of human epidemiological studies on candidate biomarkers of intake of specific foods, food groups, and food components. The largest number of candidate biomarkers were identified for intakes of cereals and beverages, and the least for biomarkers of dairy and legume intake. The most extensively validated candidate biomarkers are those reflecting cereal intake, both in terms of addressing validation criteria and replication across multiple studies. Fundamental validation criteria that are most often unreported include biomarker specificity, reproducibility over time, dose response, and for some promising candidate biomarkers, correlation with habitual food intakes. Putative biomarkers and assessment of their validity per category of dietary exposures are provided below. Detailed references can be found in the narrative summaries for each dietary exposure (see Text S1 in the Supporting Information online).

### Biomarkers of dairy intake

There are 6 main chemical classes of biomarkers of dairy intake (ie, milk, cheese, and yogurt): (1) long-chain fatty acids and *trans*-fatty acids; (2) medium-chain fatty acids; (3) phosphatidylcholines, lysophosphatidylcholines, and cholesterol esters; (4) sugars; (5) quinolone derivatives; and (6) sphingomyelins.^31–34^ Other metabolites that do not fit into these classes include dairy additives, such as undecanoic acid.^41^ Caprate, a medium-chain fatty acid, has established plausibility, as it is a component in animal fat; however, because it is a component of all animal fat, and some plant and seed oils, it is nonspecific. The long-chain fatty acids pentadecanoic acid and heptadecanoic acid are synthesized by bacterial flora in ruminants; however, they are also found in meat, rendering them nonspecific to dairy. Sugars found in dairy are milk constituents, including lactose, galactose, and their metabolites, but can also be extracted from some fruits. Additionally, galactonate, a metabolite of galactose, is a product of hepatic glucose metabolism and thus can be either exogenous or endogenously derived. However, galactonate may reflect dairy intake in populations with high intakes.^42^ Quinolone derivatives are used as antibiotics, and thus they are not specific to dairy. Blood and urine are common biospecimen sources of dairy intake biomarkers, but long-chain fatty acid dairy biomarkers are detectable in adipose tissue and erythrocytes. Dairy intake biomarkers have been analyzed from human samples using gas chromatography–MS (GC-MS), liquid chromatography–MS (LC-MS), GC coupled with a flame ionization detector (GC-FID), and NMR. Using these techniques, low to moderate correlations with dairy intake have been observed for long-chain fatty acids, such as pentadecanoic acid (15:0), myristic acid (14:0), and *trans*-palmitoleate (*trans*-16:1n-7) in both plasma and adipose tissue.^43–52^ Galactonate, sphingomyelins (SMs), and medium-chain fatty acids have low to moderate correlations with the consumption of dairy products, and lactose has a low correlation with dairy intake.^52–55^ Moderate correlations with dairy consumption have been observed for both serum cholesterol esters and 2,8-quinolinediol based on 7-day FRs.^56^ Studies that reported on half-lives of these candidate dairy biomarkers were not found. Fair to good mean ICC values have been observed for heptadecanoic acid (17:0), *trans*-palmitoleic acid (*trans*-16:1n-7), and pentadecanoic acid (15:0) (ICCs ranging from 0.52 to 0.72 measured over 2 to 3 y).^57^ Additionally, among fatty acid derivatives, SMs, and quinolone derivatives, good to excellent mean ICC values were also observed for *N,N,N*-trimethyl-5-aminovalerate (0.87), 3-bromo-5-chloro-2,6-dihydroxybenzoic (0.75), SMs (d17:2/16:0, d18:2/15:0) (0.65), and quinate (0.81) over 6 months.^52^ Overall, pentadecanoic acid, myristic acid (14:0), *trans*-palmitoleate, and galactonate appear to be promising candidate biomarkers of dairy intake. Other biomarkers are less specific to dairy, and therefore may be suboptimal.

### Biomarkers of meat intake

Most proposed meat intake biomarkers are in the following chemical classes: (1) peptides, (2) amino acids, and (3) amino acid derivatives. They are either present in meat or formed during digestion of meat in the gut.^29^ Examples include carnosine, acetylcarnitine, 4-hydroxyproline, 3-methylhistidine, and anserine. Trimethylamine N-oxide (TMAO), a compound that has been repeatedly associated with meat intake, is a metabolite of choline and phospholipids and a metabolite of l-carnitine. However, its use as a meat biomarker is limited in populations with fish consumption, as fish naturally contains high concentrations of TMAO.^58^^,^^59^ Other biomarkers may be specific for heated meat products, such as *N*-nitrosoproline formed during heating of cured meat products^60^ and heterocyclic amines (MeIQx, PhIP) that are formed when amino acids react with creatinine during thermal processing of meat and meat products.^61^ Syringol metabolites are products of wood pyrolysis present in smoke and in smoked-meat products.^62^ Piperine and piperettine are pepper alkaloids associated with processed-meat intake (eg, sausage and salami); however, their use as a meat biomarker may be limited in populations using high amounts of pepper via other food sources than meat.^63^

These biomarkers and biomarker precursors are measurable in meat and meat products, establishing their plausibility. Comprehensive meat-composition data are lacking in available food-composition tables and databases, making it difficult to assess specificity. The specificity of 3-methylhistidine and anserine (a dipeptide of 3-methylhistidine and alanine) has been more extensively examined than other candidate biomarkers for meat and meat product intake. Both compounds show high concentrations in chicken and low concentrations in other meats.^64–67^ However, some of these amino acids and peptides form in human tissues, which may limit their sensitivity as biomarkers. Robust correlations were observed between some biomarkers and meat intake, although some studies that identified acetylcarnitine, 4-hydroxyproline, and 3-methylhistidine as candidate biomarkers of total meat intake did not report correlation values.^58^^,^^68^ Correlations with the intake of specific meat products were also studied. 3-Methylhistidine was highly correlated with intakes of poultry/chicken and may be a biomarker of such food intakes.^58^^,^^69^^,^^70^ Syringol sulfate and piperine increased significantly across low, moderate, and high levels of habitual intake of smoked meat and sausage, respectively.^62^^,^^63^ Reproducibility over time (ICC) was fair for several compounds in urine, including for 3-methylhistidine (0.42), anserine (0.40), and acetylcarnitine (0.48).^71^^,^^72^ In blood, ICC values were poor to good for 3-methylhistidine (0.07–0.66) and poor to fair for 4-hydroxyproline (0.17–0.51), acetylcarnitine (0.34–0.55), and piperine (0.55).^73–75^ There are correlated foods that could be evaluated as potential confounders, such as smoked fish for syringol sulfate or pepper for piperine. Overall, studies have proposed a variety of biomarkers of meat intake, but many lack comprehensive validation. Biomarkers with the greatest level of validation according to the criteria include acetylcarnitine and 4-hydroxyproline for total meat intake, 3-methylhistidine and anserine for chicken intake, syringol sulfate for smoked-meat intake, and piperine for sausage intake.

### Biomarkers of fish and seafood intake

Several molecules belonging to (1) furan acids, (2) fatty acids, and (3) amine oxides and their derivatives are proposed biomarkers of fish and seafood intake. 3-Carboxy-4-methyl-5-propyl-2-furanpropanoic acid (CMPF) is a metabolite formed in humans from dietary furan fatty acids, which are most abundant in fish.^76^^,^^77^ Dietary furan fatty acids have also been measured in very low concentrations in green plants, mushrooms, vegetable oils, and butter.^76^^,^^77^ However, those foods were not associated with CMPF concentrations in fasting plasma in a randomized controlled trial.^78^ In cross-sectional studies in diverse free-living populations, CMPF has been associated with intakes of fish (dark, oily, and total) and shellfish, but not other foods.^46^^,^^54^^,^^55^^,^^79^ The 3 most abundant omega-3 (n-3) polyunsaturated fatty acids (PUFAs) in fish oil are eicosapentaenoic acid (EPA; *cis*-20:5n-3), docosahexaenoic acid (DHA; *cis*-22:6n-3), and docosapentaenoic acid (DPA; *cis*-22:5n-3). Accordingly, each has been associated with seafood intake and, more specifically, fish intake in human metabolomics studies. Thus, these fatty acids may both reflect fish oil and fatty fish intake. A larger number of studies have identified circulating levels of EPA and DHA, as opposed to DPA, as a candidate biomarker of fish intake.^44^^,^^46^^,^^54^^,^^55^^,^^79^^,^^80^ Additionally, 1-docosahexaenoylglycerophosphocholine (a DHA lysophosphatidylcholine and a derivative of fish oils) measured in fasted serum/plasma and nonfasted serum has been associated with total and oily fish consumption.^46^^,^^79^ Another promising candidate biomarker of fish intake is TMAO, as it is abundant in fish and seafood. However, TMAO is not seafood specific, especially in populations with low seafood intake, since, as noted above, it is associated with meat intake. The gut microbiota also generates TMAO from choline, betaine, and carnitine. However, 3 separate controlled-feeding studies found that TMAO measured in 24-hour and spot urine increased with white and fatty fish intake, or fatty fish intake alone.^58^^,^^81^^,^^82^ One of these studies replicated the finding in a cross-sectional analysis in a subset of participants from the European Prospective Investigation into Cancer and Nutrition (EPIC) in urine and plasma.^58^ No information on half-lives was available for candidate seafood intake biomarkers. High (>0.6) ICC values have been observed for serum and plasma CMPF, DHA, EPA, and TMAO (ICCs ranging from 0.55 to 0.99 measured over 4 wk to 3 y).^52^^,^^73^^,^^83^^,^^84^

### Biomarkers of vegetable intake

There are 9 classes of biomarkers of vegetable intake: (1) carotenoids, (2) tocopherols, (3) phenolic acids and derivatives, (4) flavonoids, (5) isoflavonoids, (6) retinol, (7) ascorbic acid, (8) carboxylic acids and derivatives, and (9) lipids and lipid-like molecules. While, in general, most vegetable biomarkers are associated with multiple vegetable types, there are some candidate biomarkers with more specificity. For instance, sulforaphane and *S*-methylcysteine are primarily found in cruciferous vegetables.^52^ Garlic is a primary source of the sulfoxide alliin and *S*-allylcysteine, while ergothioneine is an amino acid constituent in mushrooms. However, these compounds are not unique to these specific vegetables.^52^^,^^54^  *N*-acetylalliin and *S*-allylcysteine may be more specific markers of allium vegetable intake.^85^ Vegetable-related metabolites are detectable in blood, 24-hour urine, or spot urine samples using high-performance LC-MS, time-resolved fluorescent immunoassay (TR-FIA), and capillary electrophoresis–time of flight MS (CE-TOF-MS). Using these methods, blood carotenoids have shown moderate to strong correlations with vegetable intake.^52^^,^^86–94^ Of the carotenoids, only α-carotene consistently had a moderate correlation with total vegetable intake (including cooked vegetables) as well as with carrot intake.^86–88^^,^^92–95^ Additionally, carotene diol had a moderate correlation with leafy green and cruciferous vegetable intake, and β-cryptoxanthin had a moderate correlation with cucumber intake.^52^^,^^92^ Retinol had moderate correlations with both onion and leafy green vegetable intakes, and vitamin C with total vegetable, leafy green vegetable, root vegetable, and onion intakes.^88^^,^^96^ Moreover, alliin and *S*-allylcysteine had moderate correlations with garlic intake, and weak to moderate correlations were observed between ergothioneine and mushroom intake, *N*-acetylalliin, and allium vegetable intake and *S*-methylcysteine and cruciferous vegetable intake.^52–54^^,^^97^ Studies that reported half-lives of these candidate vegetable intake biomarkers were not identified. Excellent ICCs over 6 to 12 months were observed for carotene diol (0.79–0.83), α-carotene (0.83), ergothioneine (0.86), and lutein (0.80) in 2 studies.^52^^,^^91^ Overall, the most validated dietary biomarker candidates for garlic intake are alliin and *S*-allylcysteine, while ergothioneine is a potential biomarker for mushroom intake. *S*-Methylcysteine is a potential candidate biomarker for cruciferous vegetable intake; however, its correlation is weak, and it is also a constituent of beans.^52^ While α-carotene has a moderate correlation with total vegetable intake, it correlates with total fruit intake, rendering it a nonspecific biomarker for vegetable intake.

### Biomarkers of legume (including pulses, seeds, and peanuts) and tree nut intake

Legumes (fabaceae or leguminosae) are a diverse family of flowering plants that are an important part of traditional diets worldwide. Examples of foods within the legume family include pulses (beans, lentils, and peas) and peanuts. Soy beans contain a high content of isoflavone components, including genistein and *O*-desmethylangolensin (O-DMA).^17^^,^^98^ Candidate biomarkers of soy intake include blood or urine genistein and daidzein.^99–101^ Although other beans like peanuts contain low concentrations of these compounds, specificity is otherwise high.^102^ Both isoflavones have a relatively short plasma half-life of approximately 6 hours for genistein and 5 hours for daidzein,^103^^,^^104^ which may limit their application as soy biomarkers to populations with a frequent consumption of soy products. For example, studies in Asian populations that regularly consume soy products have demonstrated high reproducibility of soy–biomarker associations over time.^105–108^ O-DMA is a microbial metabolite formed from daidzein by the gut microbiota. However, its production depends on human gut microbial composition, limiting its use.^109^ Furthermore, urinary excretion of O-DMA is weakly associated with soy food intake.^110^ Overall, genistein and daidzein provide robust estimates of soy intake in populations frequently consuming soy products. More limited information is available on other legumes such as peanut, different types of beans, and pulses.^79^^,^^80^^,^^105^ Guertin et al^46^ and Playdon et al^55^ found that the serum metabolites 4-vinylphenol sulfate and tryptophan betaine reflected peanut or nut consumption with weak correlation to habitual intake and excellent 1-year reproducibility for tryptophan betaine (ICC = 0.74). Tryptophan betaine was also associated with habitual nut intake in several other studies. 4-Vinylphenol sulfate and tryptophan betaine are both xenobiotics previously identified in roasted peanuts or legumes.^46^ 2-Isopropylmalic acid, asparaginyl valine, and *N*-carbamoyl-2-amino-2–(4-hydroxyphenyl) acetic acid were observed to increase in a dose-dependent manner with pea intake, but studies addressing correlations with habitual intakes in free-living individuals are lacking.^111^^,^^112^ Some authors have also looked at correlations of plasma lipid metabolites, such as sphingomyelins (C24:0 and C22:0) and ceramides (C24:0), and intakes of nuts and peanuts.^113^ However, more studies are required to identify the plausibility and specificity of this association with nut intake. Pipecolic acid is a promising serum and urine biomarker of dry bean intake based on findings from a 4-week dietary intervention study.^114^ However, studies in larger, free-living populations are required to further establish its specificity and reproducibility.^52^^,^^98^

### Biomarkers of fruit intake

Biomarkers of fruit intake fall into 7 general classes as follows: (1) proline and derivatives, (2) flavonoids, (3) carotenoids, (4) threitol (a xylose metabolite), (5) ascorbic acid, (6) inositol isomers, and (7) dopamine sulfate. While specificity has not been determined for most of these compounds, proline betaine is specific to citrus fruit, phloretin is specific to apples, and dopamine sulfate is specific to bananas.^115–118^ Moreover, grapefruit is a source of flavanones, especially naringenin.^119^ The color of fruit depends on the type of carotenoid pigment. Notably, lycopene, which gives fruits and vegetables a red color, is a metabolite of tomatoes, and β-cryptoxanthin, which provides a yellow-orange color, is specific to certain fruits, including orange, tangerine, and papaya.^120^^,^^121^ These fruit metabolites have been measured in blood and urine, primarily using LC-MS. Moderate to strong correlations have been observed for proline derivatives with habitual intake of citrus fruits.^46^^,^^52–54^^,^^97^ Citrus fruit intake has also been observed to have a moderate to strong correlation with β-cryptoxanthin, and lycopene has a moderate to strong correlation with tomato intake among free-living individuals in population studies.^86^^,^^88^^,^^95^^,^^122^ Additionally, other carotenoids, including zeaxanthin, lutein, α-carotene, and β-carotene, measured in blood have moderate to high correlations with total fruit intake.^87^^,^^92^^,^^123^^,^^124^ Strong correlations have been observed for citrus flavonoids with citrus fruit intake, weak to moderate correlations have been reported for phloretin with apple intake, and moderate to strong correlations have been observed in both urine and plasma for dopamine sulfate and banana intake.^54^^,^^86^^,^^95^^,^^125^ Few studies have quantified the half-lives of metabolites of fruit intake. Of those assessed, flavanones and dopamine sulfate have very short half-lives (ie, <2 h).^125–127^ Fair to good ICC values have been observed for proline betaine (0.35–0.50), and good to excellent reliability was observed for various carotenoids (0.58–0.84), methyl glucopyranoside (0.62), 4-hydroxychlorothalonil (0.85), and γ-tocopherol/β-tocopherol (0.69) over 6 months to 1 year.^46^^,^^52^^,^^91^ Using data from controlled-feeding or intervention studies, proline betaine and flavanones showed a linear dose response to citrus intake, as did xylose with apple intake and dopamine sulfate with banana intake.^126^^,^^128–130^ Overall, proline betaine, β-cryptoxanthin, and flavanones appear to be more robust candidate biomarkers of citrus fruit intake. The strongest candidate dietary biomarker for apple intake is phloretin, whereas lycopene is a strong candidate biomarker for tomato intake. While dopamine sulfate is a banana derivative with a robust correlation with banana intake, it is also an endogenous molecule, which may limit its use.

### Biomarkers of cereal food intake

Candidate biomarkers of cereal food intake include molecules associated with intakes of whole-grain wheat and rye and their bran (total alkylresorcinols [ARs], homologues C17:0–C25:0, C17:0 to C21:0 ratio, and AR metabolites 3,5-dihydroxybenzoic acid [-DHBA], 3,5-dihydroxyphenyl propanoic acid [-DHPPA], 3,5-dihydroxyphenyl pentanoic acid [-DHPPTA]), whole-grain oats (avenacoside [AVE] A and B, avenanthramides [AVAs], and their metabolites), and of sourdough fermented rye (2-hydroxy-*N*-[2-hydroxyphenyl]acetamide [HHPAA] and *N*-[2-hydroxyphenyl]acetamide [HPAA]). Moreover, metabolomics analyses have revealed several biomarker (>18 compounds) candidates including AR metabolites (DHPPA and DHPPTA), benzoxazinoid compounds or derived metabolites (2-aminophenol-sulphate, HHPAA, HMBOA, HPAA, HPPA), and microbial products of phenolic compounds (eg, hydroxybenzoic acid glucuronide, dihydroferulic acid sulfate, and enterolactone conjugates) in urine samples associated with habitual bread consumption (both whole-grain and refined-grain breads of different types) in free-living individuals.^131^ A set of the highest ranked candidate biomarkers were combined into a panel that predicted whole-grain bread intake with low to moderate prediction performance,^131^ but many of the compounds had limited or unclear specificity with whole grains from different sources vs refined grains, which may limit their usefulness until more research has been conducted. In addition, betainized compounds have been shown to increase in plasma after the consumption of whole grains (rye and wheat) under controlled conditions, and pipecolic acid betaine increased after both whole-grain wheat and rye consumption.^132^ ARs have been measured in plasma, serum, erythrocyte membranes, or adipose tissue, whereas their metabolites are measured in plasma or urine. AVAs are analyzed in plasma and AVE is analyzed in urine samples. Benzoxazinoids and their metabolites (2-aminophenol sulfate, HHPAA, and HPAA) have been analyzed in plasma and urine.^97^^,^^133^ Odd-chain ARs are mainly found in the bran of wheat and rye, but also to a lesser extent in barley and sifted rye, with trace amounts in refined flour of wheat from contamination.^26^^,^^134^ They are not found in other food sources and are therefore specific for whole grain/bran of wheat, rye, and barley.^135^ Even-numbered AR homologues are specific to quinoa intake. ARs are stable during food processing and are not degraded, as recently suggested,^136^ but will form strong interactions with the matrix and need hot extraction to be released in hydrothermally produced foods such as bread.^137^ Several studies compared concentrations of intact ARs in plasma with estimated intakes derived from 24-HRs, FRs, and FFQs in European and American populations. The correlation coefficients are moderate to strong depending on the population and method used to estimate intake. Plasma ARs show a linear dose–response relationship at a wide intake range.^138^ A few studies have also investigated the correlation of ARs in adipose tissue biopsies with self-reported habitual intake, with similar correlations. This suggests that ARs in adipose tissue also reflect mainly short- to medium-term intake, most likely due to a rapid, dynamic turnover rate. Plasma and urine AR metabolites (3,5-DHBA, 3,5-DHPPA, 3,5-DHPPTA) in free or conjugated forms are specific to whole-grain/bran wheat and rye intake, but have also been detected after the consumption of peanuts, wort, and beer (3,5-DHBA), and after the consumption of sinapic acid and some flavonoids (3,5-DHPPA). However, the contribution of these sources is minor, and it should also be noted that some methods have wrongly identified the more common 3,4 configuration as 3,5 (3,5-DHBA and 3,5-DHPPA). Moreover, AR metabolites from spot urine and 24-hour urine show weak to moderate correlations with estimated whole-grain intake. The apparent half-life of total AR ranges from approximately 4 to 7 hours. Corresponding half-lives for AR metabolites are estimated to be 10–12 hours for 3,5-DHBA and 3,5-DHPPA and 10–16 hours for 3,5-DHBA-glycine and 3,5-DHPPTA based on plasma and urine data.^139^^,^^140^ For plasma ARs, the reproducibility over time has been shown to be fair to good over periods of 2 months to 3 years, but higher for women than for men, both for intact ARs and metabolites in plasma.^141–143^ The reproducibility of AR metabolites in plasma was similar to intact ARs, despite the longer apparent half-life.^141–143^ This may be due to unknown factors affecting the stability of AR metabolite concentrations.^144^^,^^145^

AVAs only exist in oats and therefore have excellent specificity. They are converted by the gut microbiota into their dihydro forms,^146^^,^^147^ thus differentiating different AVA metabotypes. AVE A and B are also highly specific to oats. Documented half-lives of AVAs range from 2.2 to 4.6 hours.^148^ No published studies have reported the reproducibility over time of AVAs or AVEs, plasma or urine correlations with oat intake, or dose response under controlled conditions.

Sourdough fermentation in rye generates some benzoxazinoid metabolites, but the specificity of compounds related to specific food processes remains to be elucidated. The correlation of benzoxazinoid metabolites in plasma with habitual grain intake has not been reported in population studies, but recent human intervention studies have shown large differences in their concentrations at different whole-grain (rye) intake levels, suggesting a plausible dose response.^133^ The benzoxazinoid metabolites HHPAA and HPAA in spot urine and 24-hour urine were correlated with whole-grain rye intake in the range of *r* = 0.32–0.52. One study found that a panel of biomarkers analyzed in 24-hour urine samples was associated with whole-grain rye intake.^149^ Data on half-lives and reproducibility for benzoxazinoid and their metabolites are lacking.

In summary, ARs in plasma have been validated as biomarkers of whole-grain wheat and rye intakes and are used as such in epidemiological studies. They are highly specific, increase with increased intake in a plausible dose–response manner, and are robustly correlated with estimated whole-grain intake. What primarily limits their use is the short-half life, which makes them unsuitable as biomarkers in populations with an irregular and infrequent whole-grain intake. However, in populations with a frequent intake, such as in Scandinavian countries, ARs in plasma are feasible biomarkers of whole-grain wheat and rye intake. AR metabolites in plasma and urine have an approximately similar performance as intact ARs in plasma, despite a longer apparent half-life. Twenty-four-hour urine metabolite concentrations may be strongly correlated with estimated intake, but the feasibility of 24-hour urine collection may limit their use. AVAs and AVEs in plasma and urine appear promising as biomarkers of oat intake, but further studies to establish their pharmacokinetics and dose response under controlled intake conditions in humans as well as in observational studies are highly warranted. Similar studies are also warranted to judge the feasibility of benzoxazinoid derivatives and betainized compounds as individual biomarkers of whole-grain intake and of combinations of the most promising individual markers into biomarker panels.

### Biomarkers of food component intake: sugar, alcohol, fats, and oils

#### Sugar

Biomarkers of sugar intake (sucrose) include (1) fructose and sucrose and (2) the isotopic signature δ^13^C, which does not belong to a specific chemical class. Sucrose originates directly from dietary sucrose, whereas fructose originates directly from dietary fructose and is one of the monosaccharides in sucrose. With regard to δ^13^C, photosynthetic plants discriminate carbon isotopes when fixing carbon dioxide into organic molecules. This discrimination varies depending on photosynthetic pathways. C_4_ plants (eg, corn, sugar cane) fix more of the heavy isotope ^13^C than C_3_ plants (most plant species). This is reflected in the ^13^C/^12^C ratio in sugars produced by these plants. The ^13^C abundance (^13^C/^12^C ratio; δ^13^C) is changed in human biofluids and tissues upon ingestion of sugars produced by C_4_ plants. A high consumption of added sugar (eg, sucrose from sugar cane or high-fructose syrup from corn) will influence δ^13^C. Sucrose and fructose are natural constituents of many foods and food products, and the urinary concentration of these compounds will reflect both sugars naturally present in the foods as well as added sugar, and therefore not the intake of any specific food or food group. δ^13^C reflects the intake of sugars produced by C_4_ plants and has been used in the United States as a proxy for the intake of added sugars from corn and sugar cane, hence may not be applicable for other sources of sugars, like sugar beet or natural fruit sugars. Fructose and sucrose are only measured in urine, typically using GC-MS^150^ or LC-MS,^151^ whereas δ^13^C is measured in whole blood, red blood cells, hair, breath, and plasma by isotope-ratio MS technology. Measured δ^13^C in hair samples and breath was significantly associated with dietary carbohydrate intake, particularly with sweetened beverages,^152^ and the δ^13^C of specific amino acids, particularly alanine in serum, was moderately correlated with added-sugar intake.^153^^,^^154^ Urinary fructose and sucrose have generally shown poor to modest correlations (*r* range: 0.03–0.43) with habitual sugar intake estimated by FRs and somewhat weaker when using morning spot urine samples (*r* range: 0.20–0.30).^155^ Despite modest correlations, the performance of urinary sucrose and fructose as biomarkers of habitual sucrose intake was comparable to urinary nitrogen as an established protein intake biomarker in a free-living Dutch adult population.^156^ Correlations of δ^13^C with total added-sugar and sugar-sweetened beverage intake measured by FFQs or FRs stem from studies measuring the biomarker in whole-blood samples; correlation coefficients ranged from *r* = 0.28 to 0.35 depending on the exposure and dietary instrument used.^157^ The apparent elimination half-life of sucrose is approximately 3 hours^158^ and is 39 minutes for fructose.^159^ The 50% turnover of δ^13^C was reported to be 2.5 weeks in plasma and 5.9 weeks in red blood cells,^160^ underscoring its potential to reflect long-term sugar intake. Despite the short half-life, urinary sucrose and fructose showed a modest reproducibility (ICC: 0.38–0.47) over a period of 3 years.^156^ In summary, none of the sugar biomarkers have been fully validated, but the currently available data suggest that sucrose and fructose in 24-hour urine, and possibly in morning spot urine, are promising biomarkers of total and extrinsic sugar intake. δ^13^C is also a promising biomarker of habitual added sugars from C_4_ plants in US populations where they are widely consumed, but this requires further validation.

#### Alcohol

Biomarkers of alcohol consumption are important in forensic contexts. In clinical medicine, they can verify alcohol abstinence or toxicity. Correlations between FFQ self-reported alcohol intake and ethyl glucuronide concentrations in plasma or urine are moderate to strong (*r* = 0.26–0.36 in serum; *r* = 0.20–0.60 in urine). Stronger correlations have been observed for phosphatidylethanol (PEth) measured in whole blood (*r* = 0.26–0.79). Despite a short half-life of ethyl glucuronide (ie, 2.5 h), the 6-month to 1-year reproducibility over time was reported as moderate (ICC = 0.27^46^ and ICC= 0.57^52^, respectively). PEth has a longer half-life of 2–9 days depending on specific compounds evaluated, but data on its reproducibility over time are currently lacking. Other compounds may be useful biomarkers for specific alcoholic beverages. Compounds such as humulinone, isoxanthohumol, and 2,3-dihydroxy-3-methylvaleric acid have been suggested as candidate biomarkers of beer intake,^161^^,^^162^ and a combination of 7 biomarkers originating from the various raw materials used in beer production was also proposed as a biomarker of beer intake.^163^ Observational studies associating self-reported beer intake with putative biomarkers of beer intake are lacking, as are data on biomarker half-lives and reproducibility over time. One study estimated the half-life of isoxanthohumol to be 20–28 hours.^164^ Suggested biomarkers of wine intake include compounds produced during wine fermentation, small organic acids originating from the wine, and wine polyphenols and their metabolites including compounds such as resveratrol, resveratrol glucuronide and sulfates, hydroxytyrosol, tartaric acid, 2,3-butanediol, gallic acid, and gallic acid ethyl ester.^163^^,^^165–172^ The correlations between habitual self-reported intake of wine and biomarkers measured in plasma, such as 4-*O*-methylgallic acid and gallic acid ethyl ester sulfate, range from *r* = 0.30 to 0.44, depending on the dietary instrument used. Correlations of urinary biomarkers with wine intake range from *r* = 0.22 to 0.69. Half-lives are typically short and range between 1 and 9 hours.^173–175^ Many clinically used alcohol-exposure biomarkers have a short half-life, which limits their use in epidemiological investigations of habitual alcohol intake if consumption is sporadic.^176^ Molecules suggested to reflect total alcohol intake include ethyl glucuronide, ethyl sulfate, 2-phenylethanol glucuronide, PEth,^161^^,^^177^^,^^178^ and more recently, 2-hydroxy-3-methylbutyric acid.^20^ The reproducibility has been estimated to be ICC = 0.42–0.67 for different compounds in plasma and urine.^52^^,^^83^ Most of the suggested biomarkers have been measured by LC-MS/MS and a few by GC-MS and/or NMR.

In summary, ethyl glucuronide and PEth are promising biomarkers of habitual total alcohol intake, but studies on the correlations with habitual self-reported intake in free-living populations and reproducibility over time are still lacking for PEth. Putative biomarkers of specific alcohol beverages, such as isoxanthohumol for beer intake and tartaric acid, 4-*O*-methylgallic acid, and gallic acid ethyl ester sulfate for wine intake, are promising; however, studies on the magnitude of correlation with reported intake, reproducibility, and specificity are needed. For example, 4-*O*-methylgallic acid (a human metabolite of gallic acid abundant in wine but also in tea) will not be specific enough in populations consuming tea. On the other hand, gallic acid ethyl ester, a metabolite formed in wine by esterification of ethanol (abundant in wine and absent in tea) with gallic acid, is more specific to wine intake.

#### Fats and oils

Biomarkers of fat and oil intake include several chemical classes: (1) unsaturated fatty acids, (2) saturated fatty acids, and (3) amino acid derivatives. Pentadecanoic acid or pentadecylic acid (15:0), heptadecanoic acid or margaric acid (17:0), and myristic acid (14:0) are long-chain fatty acids typically associated with butter consumption.^43^^,^^46^^,^^51^^,^^56^^,^^79^^,^^179^^,^^180^ Palmitelaidic acid or *trans*-16:1n-7 is also a long-chain fatty acid typically found in butter and margarine. Very-long-chain fatty acids include EPA (*cis*-20:5n-3), DHA (*cis*-22:6n-3), and DPA (*cis*-22:5n-3) and other n-3 PUFAs. EPA and DHA are the 2 most abundant n-3 fatty acids in fish oil and in marine mammal fat,^181^ and DHA is the third most abundant long-chain n-3 fatty acid in fish oil.^181^ These fatty acids are all detectable in blood. Other biomarkers associated with fat/oil intake, such as creatine, N-acetylglutamine, and N-acetyltyrosine, have been measured in overnight urine samples and have been associated with different types of fats and oils, including butter, margarine, meat fat, mayonnaise, salad dressing, oil used for cooking, and shortening.^55^ Pentadecanoic acid (15:0) and heptadecanoic acid (17:0) are synthesized by bacterial flora in ruminants and are not produced in humans, whereas very-long-chain fatty acids (eg, EPA, DHA, DPA, and other n-3 PUFAs) are found in fish oil. Correlations of fat/oil intake with EPA, DHA, and DPA have been observed in serum and plasma (fasted and nonfasted).

Correlations of fat and oil biomarkers with habitual dietary intake ranged up to 0.19 for 17:1, 0.40 for heptadecanoic acid (17:0), 0.47 for pentadecanoic acid (15:0), and 0.22 for *trans*-palmitoleic acid (trans-16:1n-7). Reproducibility (ICC) over time was moderate for 17:0 (0.50), 16:1 (0.60), and 15:0 (0.70). Most of these biomarkers are constituents of related foods, like fish and fish products for the very-long-chain fatty acids and other dairy products for 15:0 and 17:0.

Overall, a variety of blood and urine biomarkers for the intake of fats and oils have been proposed, although few have been fully validated. Some of the biomarkers of fats and oils also reflect specific food intakes such as marine foods (long-chain n-3 PUFAs) and dairy foods (C15:0 and C17:0). Urine creatine, N-acetylglutamine, and N-acetyltyrosine are promising fat and oil biomarkers. Blood DHA (*cis*-22:6n-3), DPA (*cis*-22:5n-3), EPA (*cis*-20:5n-3), n-3 PUFAs, margaric acid (17:0), methyl palmitic acid (C17H34O2), and pentadecylic acid (15:0) are also promising.

### Biomarkers of tea intake

Candidate biomarkers of tea intake include the following: (1) gallic acid and its derivatives, (2) catechins and catechin metabolites, (3) carboxylic acid and its derivatives, and (4) flavonoids. With regard to the plausibility of these biomarkers, catechins, gallic acid, and flavonols are phenolic compounds found in tea leaves, with the amount depending on the variety of tea.^182^ The amino acid theanine is also a constituent in tea.^54^ Catechins, such as epigallocatechin and epicatechin, are particularly abundant in tea. Catechins are also present in other foods, however, such as fruits, chocolate, some vegetables, and nuts. Similarly, chocolate and wine contain gallic acid, which makes the biomarker specific only for some populations depending on intakes of these foods.^183^^,^^184^ Theanine, while primarily found in tea, is also found in mushrooms. Tea-associated metabolites have been detected in blood and 24-hour urine by LC-MS and CE-TOF-MS, and in spot urine with analysis by LC-MS. Moderate to high correlations with habitual tea intake have been observed for urine gallic acid metabolites 4-*O*-methylgallic acid and methylgallic acid sulfate.^184^^,^^185^ Additionally, theanine measured in blood had moderate correlations with tea intake, and the correlations tended to be higher in caffeinated or nonherbal teas.^52–54^ Although epigallocatechin, epicatechin, and other catechin metabolites have shown dose–response relationships to tea intake in interventional studies, they show low to moderate correlations with habitual tea intake measured by FFQs in population studies.^95^^,^^184^ Kaempferol was the only flavonoid showing a moderate correlation with habitual tea intake.^86^^,^^95^ Moderate ICC values were observed for 3-methoxycatechol sulfate and theanine.^52^ Overall, 4-*O*-methylgallic acid, methylgallic acid sulfate, and theanine appear to be the most promising candidate biomarkers for tea intake, despite some limitations in their specificity.

### Biomarkers of coffee intake

Coffee intake biomarkers derive from several chemical classes: (1) caffeine and its metabolites, (2) phenolic acids, (3) organic acids, (3) niacin derivatives, and (4) roasting compounds, among others. Coffee beans and coffee brews contain a number of characteristic compounds, including caffeine, 5-caffeoylquinic acid (chlorogenic acid), feruloylquinic acid, and trigonelline, which is derived from the metabolism of niacin (vitamin B_3_). These compounds are eventually transformed by the gut microbiota or human tissues into a number of metabolites such as theophylline and theobromine (from caffeine), caffeic acid, dihydrocaffeic acid and quinic acid (from chlorogenic acid), or nicotinic acid (from trigonelline). In addition to these compounds naturally present in coffee beans, other compounds like diketopiperazines and *N*-(2-furoyl)glycine are formed during roasting of beans and show increased levels in highly roasted coffee beans.^186^ All of these compounds can be absorbed in the gut and are found in blood and urine after coffee consumption. Some of these compounds can also be found in other dietary sources (eg, caffeine in tea). However, for most compounds, their concentrations in other foods or beverages are much lower in comparison with coffee, resulting in good specificity as biomarkers of coffee intake. Most biomarker studies have focused on biomarkers of intake of generic coffee (ie, any coffee type and processing method). However, combinations of biomarkers could be used to study intakes of particular types of coffee, such as the caffeine and trigonelline ratio for caffeinated over decaffeinated coffee.^187^^,^^188^

Coffee biomarkers are detectable in both blood (plasma or serum) and urine. Caffeine and its metabolites have low to moderate correlations with habitual coffee intake. Trigonelline and quinic acid have been most strongly correlated with coffee intake (*r* values up to 0.61 and 0.77, respectively).^187^ Among European populations, biomarkers showing the highest correlations with coffee consumption were found to vary, possibly reflecting the different types of coffee brews consumed in each country.^188^ Most coffee biomarkers have short half-lives (maximum of 5 h). However, trigonelline and quinic acid have high mean ICC values over time (0.66 and 0.81 over 6–12 mo, respectively), likely due to the frequency of coffee consumption.^52^^,^^189^

Overall, trigonelline and quinic acid appear to be better qualified biomarkers of coffee intake than other candidates. Combinations of these biomarkers with compounds such as caffeine, diketopiperazines, and *N*-(2-furoyl)glycine should be tested in future studies as they may provide information on the type of coffee consumed.

### Summary of the extent of candidate dietary biomarker validation

The extent of validation of selected dietary biomarkers that appear to be the most promising candidates based on the validation criteria is summarized in Table 2. All top candidate biomarkers have been studied in either serum or plasma and, in some cases, in urine. The meat biomarker acetylcarnitine and the tea biomarker 4-*O*-methylgallic acid were studied in urine samples only. The top legume, fish/seafood, whole-grain, and coffee biomarkers are specific to those food groups, whereas others may be associated with 1 or more other dietary exposures. The reproducibility over time has been investigated for most of the top dietary biomarkers, being moderate for the top vegetable, legume, fish/seafood, and coffee biomarkers. For other biomarkers, the reproducibility over time ranged from weak to moderate. As expected, the magnitude of correlation of the top dietary biomarkers with short-term food intake (ie, measured by 24-HRs or FRs) tended to be stronger than the correlation with long-term food intake (ie, measured by FFQs). The strongest correlations with habitual food intake per biomarker ranged from 0.28 (ergothioneine) to 0.62 (genistein). None of the top dietary biomarker candidates have recognized nondietary determinants, such as major confounding factors or effect modifiers, except for ARs, which were shown to have higher and more variable plasma concentrations for men than for women, and stronger ICCs over time for women than for men.^143^ All top dietary biomarkers can be measured by both LC-MS and GC-MS; acetylcarnitine and 3-methylhistidine can also be captured by NMR. Finally, few of the top biomarkers have been evaluated for dose response with the food source. Steps to fully validate this panel of dietary biomarkers include further studies on reproducibility over time and dose–response feeding studies.

**Table 2:** Candidate dietary biomarkers with the highest level of validation

Food group and most validated candidate biomarkers	Biospecimen	Specificity	Parent compound	Reproducibility over time (ICC)	Correlation (*r*) with intake measured by 24-HR or FR	Correlation (*r*) with intake measured by FFQ	Nondietary determinants	Analytical platform	Dose response
Vegetable									
Ergothioneine (mushroom)	Serum/plasma	No (organ meats, beans)	No	0.86	0.26	0.19–0.28	No	LC/GC	NA
α-Carotene (total vegetable)	Serum/plasma	No (fruit)	Yes	0.83	0.04–0.52	0.17–0.50	No	LC/GC	NA
Fruit									
Proline betaine	Serum/plasma	No (some cheese, alfalfa, artichoke, seafood)	No	0.35–0.50	0.25–0.77	0.15–0.55	No	LC/GC	NA
Proline betaine	Urine	No (some cheese, alfalfa, artichoke, seafood)	No	NA	0.48–0.80	0.43–0.50	No	LC/GC	Yes
β-Cryptoxanthin	Serum/plasma	No (total fruit)	Yes	0.50–0.77	0.04–0.49	0.11–0.57	No	LC/GC	NA
Legumes									
Genistein	Serum/plasma	Yes	Yes	0.22–0.93	0.51	0.33–0.62	No	LC/GC	NA
Genistein	Urine	Yes	Yes	0.33	0.53	NA	No	LC/GC	NA
Daidzein	Serum/plasma	Yes	Yes	0.11–0.92	0.49	0.09-0.49	No	LC/GC	NA
Daidzein	Urine	Yes	Yes	0.17	0.44	NA	No	LC/GC	NA
Dairy									
Pentadecanoic acid	Serum/plasma	No (meat)	Yes	0.72	0.17–0.54	0.03–0.36	No	LC/GC	NA
Pentadecanoic acid	RBCs	No (meat)	Yes	NA	NA	0.06–0.30	No	LC/GC	NA
Pentadecanoic acid	Adipose tissue	No (meat)	Yes	NA	0.16–0.72	0.09–0.39	No	LC/GC	NA
*trans*-9-Hexadecenoic acid	Serum/plasma	No (partially hydrogenated vegetable oil, red meat)	Yes	0.57	NA	0.06–0.39	No	LC/GC	NA
*trans*-9-Hexadecenoic acid	RBCs	No (partially hydrogenated vegetable oil, red meat)	Yes	NA	NA	0.07–0.32	No	LC/GC	NA
Meat									
Acetylcarnitine	Urine	NA	Yes	0.48	NA	0.26–0.32	No	LC/GC/NMR	Yes
4-Hydroxyproline	Serum/plasma	No (fish)	Yes	NA	NA	0.075–0.36	No	LC/GC	NA
Chicken									
3-Methylhistidine	Serum/plasma	NA	NA	0.07–0.66	NA	0.40–0.54	No	LC/GC/NMR	NA
Fish/shellfish									
CMPF	Serum/plasma	Yes	No	0.33–0.99	0.47	0.24–0.47	No	LC/GC	NA
CMPF	Urine	Yes	No	NA	NA	0.26–0.27	No	LC/GC	NA
DHA	Serum/plasma	Yes	Yes	0.55–0.95	NA	0.26–0.37	No	LC/GC	NA
DHA	Adipose tissue	Yes	Yes	NA	NA	0.15	No	Other	NA
Whole-grain wheat and rye (tabulated information for biomarker candidates of other cereals are missing)									
Alkylresorcinols	Serum/plasma, adipose tissue	Yes	Yes	0.42–0.64	0.40–0.55	0.17–0.39	Sex	LC/GC	Yes
DHBA	Plasma/urine	Yes	No	0.17–0.55	0.35–0.57	0.10–0.43	No	LC/GC	Yes
DHPPA	Plasma/urine	Yes	No	0.22–0.45	0.26–0.57	0.23–0.46	No	LC/GC	Yes
DHPPTA	Plasma/urine	Yes	No	0.63	0.17–0.52	NA	No	LC/GC	NA
Tea									
4-*O*-Methylgallic acid	Urine	No (wine, nuts, some berries)	No	NA	0.14–0.62	0.41–0.50	No	LC/GC	NA
Theanine	Serum/plasma	No (mushroom)	Yes	0.60	0.28–0.51	0.23–0.50	No	LC/GC	NA
Coffee									
Trigonelline	Serum/plasma	Yes	Yes	0.66–0.84	NA	0.12–0.66	No	LC/GC	NA
Quinic acid	Serum/plasma	Yes	No	0.81	0.74	0.16–0.77	No	LC/GC	NA
Fats and oils									
EPA (*cis*-20:5n-3)	Serum/plasma	Yes	Yes	NA	NA	0.29–0.44	No	LC/GC	NA
DPA (*cis*-22:5n-3)	Serum/plasma	Yes	Yes	NA	NA	0.24–0.38	No	LC/GC	NA
DHA (*cis*-22:6n-3)	Serum/plasma	Yes	Yes	NA	NA	0.25–0.36	No	LC/GC	NA
Alcohol									
PEth	Whole blood	Yes (total alcohol)	Yes	NA	NA	0.26–0.79	No	LC	NA
Ethyl glucuronide	Urine	Yes (total alcohol)	No	0.27–0.57	NA	0.20–0.60	No	LC	NA
Sugar									
Sucrose	Urine	Yes (sucrose)	Yes	0.38–0.47	0.20–0.30	NA	No	LC/GC	Yes
δ^13^C	Blood	Yes (C4 plant-derived sugars)	Yes	NA	NA	0.28–0.35	No	GC/LC	NA

*Abbreviations*: CMPF, 3-carboxy-4-methyl-5-propyl-2-furanpropanoate; DHA, docosahexaenoic acid (22:6n-3); DHBA, dihydroxybenzoic acid; DHPPA, dihydroxyphenyl propanoic acid; DHPPTA, dihydroxyphenyl pentanoic acid; DPA, docosapentaenoic acid; EPA, eicosapentaenoic acid; FR, food record; GC, gas chromatography; ICC, intraclass correlation coefficient; LC, liquid chromatography; NA, not available; NMR, nuclear magnetic resonance spectroscopy, PEth, phosphatidylethanol; RBC, red blood cell; 24-HR, 24-hour dietary recall.

## CONCLUSION 

Many biomarkers of the foods and food components outlined in the current review have been suggested over the years, but very few have been fully validated. The most promising biomarkers of each food category assessed are listed in Table 3, which notes critical gaps needed to be addressed to be considered validated according to criteria outlined by the FoodBAll consortium, which were modified to consider criteria specific for epidemiological studies.^25^

**Table 3:** Dietary biomarkers and validation criteria yet to be addressed

Dietary exposure	Dietary biomarker(s)	Information lacking for the biomarkers
Dairy	Pentadecanoic acid (15:0), myristic acid (14:0), *trans*-palmitoleate (*trans*-16:1n-7), and galactonate	Additional studies are needed to confirm myristic acid as a positive biomarker for dairy intake, and data on *trans*-palmitoleate’s correlation with habitual intake is lacking. Dose response has also not been evaluated for any of these biomarkers.
Meat	Acetylcarnitine and 4-hydroxyproline for total meat intake, 3-methylhistidine and anserine for chicken intake, syringol sulfate for smoked meat, and piperine for sausage	Limited correlation values with habitual meat intake have been published so far and replication in different populations is needed. More data on specificity are also needed.
Fish and seafood	CMPF for lean and total fish, EPA and DHA for fatty fish	Reproducibility over time is lacking for fish intake biomarkers and also correlation of intake with different types of fish across different populations for CMPF.
Vegetables	Alliin and *S*-allylcysteine in blood for garlic; *N*-acetylalliin in blood for allium vegetables and ergothioneine in blood for mushrooms; α-carotene in blood for total vegetable intake	Dose response has not been assessed for any of these 5 biomarkers. Reproducibility data are also lacking for alliin.
Legumes	Genistein and daidzein in blood and urine for soy and soy product intake in frequent consumers	More studies needed on pipecolic acid as a candidate biomarker for dry bean intake.
Fruits	Proline betaine in blood and urine as well as flavanones in urine for citrus fruit intake and β-cryptoxanthin in blood for tropical fruits; phloretin in urine for apples, lycopene in blood for tomato, and dopamine sulfate in blood, and urine for bananas; inositol in blood and urine is a promising biomarker for total fruit intake	Dose response has not been demonstrated for phloretin, proline betaine in blood, or carotenoids. No data are available for reproducibility of proline betaine in urine.
Cereals	Alkylresorcinols and their main metabolites DHPPA, DHPPTA in plasma and urine for whole-grain wheat and rye intake, and AVAs and AVEs for oat intake	Estimation of correlations between habitual whole-grain intake and DHPPTA, AVAs, and AVEs in plasma and urine as well as estimation of their reproducibility are lacking. Moreover, estimations of half-lives of AVEs are also lacking.
Sugar	Fructose and sucrose in 24-h urine collections as well as δ^13^C in whole blood	Evaluation of the correlations of fructose and sucrose in morning or spot urine samples with self-reported intake is warranted. Estimations of reproducibility are scarce for all 3 candidate biomarkers in all different matrices.
Alcohol	Ethyl glucuronide and PEth for total alcohol, isoxanthohumol for beer intake, and tartaric acid and gallic acid ethyl ester sulfate for wine	Studies on the correlations between ethyl glucuronide and PEth with self-reported intake and estimations of reproducibility are currently lacking and are warranted. Putative biomarkers of specific alcohol beverages such as isoxanthohumol for beer intake and tartaric acid and gallic acid ethyl ester sulfate for wine intake require further evaluation with regard to estimation of their sensitivity and specificity in free-living subjects. Their reproducibility also needs to be assessed.
Tea	4-*O*-Methylgallic acid and methylgallic acid sulfate in urine, and theanine in blood	Dose response has not been assessed for any of these 3 promising tea biomarkers. Only theanine has data on reproducibility. For gallic acid metabolites, possible confounding with wine intake needs to be assessed.
Coffee	Trigonelline and quinic acid in blood and urine.	Combinations with other coffee biomarkers may provide details on the type of coffee beverage consumed.
Fats and oils	Blood/plasma fatty acids, particularly long-chain polyunsaturated fatty acids, are relatively good biomarkers for the consumption of plant-based oils and fats. Very-long-chain fatty acids are promising biomarkers for oils and fats derived from seafood (including fish and marine mammals)	Dose response has not been assessed for any of these biomarkers.

*Abbreviations*: AVA, avenanthramide; AVE, avenacoside; CMPF, 3-carboxy-4-methyl-5-propyl-2-furanpropanoate; DHA, docosahexaenoic acid (22:6n-3); DHPPA, dihydroxyphenyl propanoic acid; DHPPTA, dihydroxyphenyl pentanoic acid; EPA, eicosapentaenoic acid; PEth, phosphatidylethanol.

Many of the most promising dietary biomarkers discussed have short to medium half-lives, ranging from 4 hours to several days. Despite this, some showed modest to good reproducibility. This is likely due to frequent the intake of the reference foods, which compensates for the short half-life in providing a stable concentration in biospecimens like blood or urine. Averaging biomarker concentrations from repeated biospecimen sampling could attenuate fluctuations in a dietary biomarker concentration that results from having a short half-life and modest reproducibility. The development of simple sampling techniques that can be performed at home, such as dried blood and urine spots, would enhance feasibility to accomplish repeated sampling on a large scale.^190^ Moreover, the small sample volumes collected with these techniques may require the development of new analytical methods. The development of novel quantitative methods to measure more comprehensive biomarker panels will also be required. Such methods could provide more efficient use of sample volumes and be more cost-effective.

Only a few candidate dietary intake biomarkers have long half-lives and could represent long-term intake in the absence of regular consumption. Biomarkers with modest to excellent reproducibility over time (measured as ICCs) were identified, suggesting a single measurement could be used to reflect long-term intake (Tables  2 and 3). Moreover, analysis of biomarker candidates in other matrices than blood compartments may reflect more long-term intake. For example, analysis of odd-chain fatty acids and carotenoids in adipose tissue biopsy samples has been shown to correlate well with long-term dairy intake^31^ and fruit intake,^191^ respectively. Other biospecimens such as hair may reflect long-term food intake as molecules tend to be retained in hair, but the development of dietary biomarkers from hair samples has rarely been attempted^192^ and may be limited for use in certain populations (eg, non-bald, no use of hair products containing chemicals like dye, etc).^193^ The formation of adducts with blood proteins or DNA may be another option for long-term reflection of dietary intake, since the half-life of DNA adducts are generally longer than for circulating compounds.^194^ However, the number of food-specific adducts is limited and more exploratory studies would be needed for biomarker discovery. Recent studies have shown that microRNAs from plant-based foods are absorbed in humans to some degree and are detectable in blood samples. They have been discussed as food intake biomarkers, but it is unlikely that they will reflect long-term intake.^195^

Although several promising dietary biomarkers have been characterized, there is still substantial work needed both to discover new biomarkers for critical foods, such as sugar-sweetened beverages, and to provide complete validation. Dietary intervention studies are needed to address that lack of dose–response data available. Many biomarker candidates are metabolites formed in the body from parent food compounds, and characterization of the factors influencing their formation is needed. Moreover, some biomarkers may reflect several food groups, such as biomarkers of fruits may also reflect the intake of fruit juices. Estimates of biomarker reproducibility in free-living populations are often missing. The field would benefit from characterization of biomarker variability and factors affecting such variability. This information will be essential to evaluate the size of populations and the number of repeated biospecimen collections needed to study their associations with health and disease outcomes in cohort studies.^73^ In some cases, fundamental data on biomarker correlation with self-reported dietary intake are also missing. The most comprehensively evaluated biomarkers include proline betaine (a biomarker of citrus intake) and ARs and their metabolites (biomarkers of whole-grain wheat and rye intake), and this is reflected by their more routine use in nutritional epidemiology. Yet, major gaps exist in the validation of other promising dietary biomarker candidates.

In most cases, dietary exposures have been reflected by single biomarkers. Although it may be practical to analyze fewer biomarkers, single molecules may lack specificity for the exposure of interest. Biomarker panels that jointly reflect individual foods, food groups, or dietary patterns have the potential to mitigate this issue.^196^ Combinations of diet-derived molecules with varying proportions in different food sources could also increase biomarker specificity. Comparing several biomarkers simultaneously could shed light on the specificity of multiple dietary biomarker profiles. Several blood metabolite signatures have been associated with adherence to specific dietary patterns.^197^ However, it is yet unclear to what extent such signatures reflect the food components of the dietary pattern per se, or if they are derived from interactions with other environmental exposures, individual or lifestyle factors, human endogenous metabolism, or gut microbiota. The field could benefit from a framework for the validation of biomarkers of dietary patterns and their interpretation.^197^

Although dietary biomarkers are promising to objectively assess dietary intake, methodological limitations, such as potential nondietary determinants; poor reproducibility due to random error associated with episodic consumption; sample instability due to collection, processing, or storage method; and analytical drift of the response of the mass spectrometer along the analysis of large series of samples that induces measurement error. Such limitations thus render the biomarkers suitable as a complement to traditional self-reported dietary assessment, rather than as an alternative. Methods to combine biomarker measurements with traditional dietary assessments can improve the precision in the ranking of intake of specific foods in observational studies, and can be used to calibrate self-reported data.^196^ To date, dietary data calibration has leveraged doubly-labeled water and urinary nitrogen as recovery biomarkers for energy and protein intakes, respectively. However, non-recovery biomarkers—namely, concentration biomarkers such as carotenoids, tocopherols, folate, vitamin B_12_, and phospholipid fatty acids—have more recently been shown to be useful to correct for systematic measurement error in self-reported nutrient intake when assessing diet and disease associations.^23^^,^^198^ This has opened the door for biomarkers beyond recovery biomarkers; thus, concentration dietary biomarkers described in the present review have the potential to correct measurement errors by calibration and to improve subject ranking of estimated food intake. For example, proline betaine recently corrected measurement errors in self-reported dietary data using a calibration approach,^198^ and plasma ARs were successfully used in combination with whole-grain intake data from FFQs to improve precision in the ranking of whole-grain intake in relation to colorectal cancer incidence.^199^ It has been posited that biomarker measures in approximately 30% of large-study populations could be adequate to generate calibration equations.

Analyses of single biomarkers as well as panels have often been conducted with a wide variety of analytical methods, which makes interpretations more difficult due to differences in results. There is a need for comprehensive, simple, and robust assays for analysis of dietary biomarkers that can be widely adopted.

In summary, efforts have been made to discover, and to a lesser extent, validate dietary biomarkers during the last 10 years. Separate comprehensive review articles on candidate biomarkers of specific food intakes have been published recently by the FoodBAll consortium and by other authors, but to our knowledge, this review provides the first comprehensive assessment of the emerged biomarker candidates according to established validation criteria adapted for epidemiological studies. This review identified specific gaps related to the validation of specific biomarkers as well as general developments needed to take the application of dietary biomarkers further in the field of nutritional epidemiology. Future studies that emphasize the validation of individual biomarkers, biomarker panels, and the development of analytical methods that capture many dietary biomarkers in a single analysis are warranted. Moreover, evaluation of their use together with other dietary assessment methods should also be further studied. There is also a need to better understand the impact of fasting status and timing of sampling for the validity and reproducibility of biomarker measurements; this is as yet unknown for most biomarkers, although it may to some degree be predictable from kinetics data. Another area for future research is to find specific biomarkers of food preparation and processing, since they may have health implications. Finally, data-driven or predefined panels of biomarkers that reflect specific dietary patterns and whole diets would be useful for future epidemiological investigations and there are promising developments in this area under way.^197^ With further developments, the field of nutritional epidemiology is therefore poised to benefit dramatically from improved dietary intake assessment, which will serve to strengthen the validity of studies on diet, health, and disease.

## Supplementary Material

nuad119_Supplementary_Data

## Supporting Information

The following Supporting Information is available through the online version of this article at the publisher’s website.

*Table S1*  **A summary of candidate dietary biomarkers identified per food category, assessment of them according to validation criteria, and their references**.

*Text S1* **Standardized summary sheets of the most promising candidate biomarkers per food category and appraisal of them according to validation criteria along with key references**.
