# Supplementary material for: Dietary biomarkers—an update on their validity and applicability in epidemiological studies
Source: Nutr Rev. 2023 Oct 3;82(9):1260–80. doi: 10.1093/nutrit/nuad119 (PMC11317775; doi:10.1093/nutrit/nuad119)
Supplement: nuad119_Supplementary_Data [file nuad119_supplementary_data.zip › nuad119_Supplementary_Data/Supplemental text 1 FINAL FINAL.docx]

**Biomarkers of alcohol intake**

A total of 119 unique compounds and 12 different panels of compounds, measured in blood or urine and showing an association to total alcohol intake and/or a specific type of alcohol, were found distributed among the various types of alcoholic beverages as described below:

- Total alcohol: 35 individual compounds and 7 compound panels.
- Beer: 9 individual compounds of which 3 merely reflect alcohol intake and one compound panel.
- Liquor: 4 individual compounds, 3 reflecting alcohol intake and one of unknown origin.
- Wine: 90 individual compounds of which at least 5 merely reflect alcohol intake and 4 compound panels.

Due to the very high number of proposed biomarkers for total alcohol and wine intake, only biomarkers reflecting these groups identified in more than one study and/or with a reported significant correlation with habitual intake ≥ 0.7 have been included in this summary.

**1. Nature of alcohol biomarkers and their specificity**

1. Biomarkers of habitual alcohol intake belong to different chemical classes:

| **Biomarker** | **Class** | **Precursor/origin** |
| --- | --- | --- |
| Alcohol | | |
| Ethyl glucuronide | O-glucuronide | Ethanol metabolism ^1^ |
| Phosphatidylethanols (Peth 16:0/16:0; Peth 18:1/18:1; Peth 16:0/18:2; Peth 16:0/18:1; Peth 18:1/16:0) | Phospholipids | Ethanol metabolism. Formed only in the presence of ethanol via the action of [phospholipase D](https://en.wikipedia.org/wiki/Phospholipase_D) (PLD). When ethanol is present, PLD substitutes ethanol for water and covalently attaching the alcohol as the head group of the phospholipid. ^2^ |
| 5-hydroxytryptophol and 5-hydroxyindole-3-acetic acid | Hydroxyindole and indole-3-acetic acid derivative | Both compounds are minor metabolites of serotonin. Alcohol intake causes a shift in the metabolism of serotonin from 5-hydroxyindole -3-acetic acid toward increased formation of 5-hydroxytryptophol due to the inhibition of aldehyde dehydrogenase by ethanol-derived acetaldehyde. Urinary excretion of 5-hydroxytryptophol has also been shown to be markedly increased for several hours following intake of foods rich in serotonin, such as bananas^3^. |
| Beer | | |
| Humulinone | Monocyclic monoterpene | Hops ^4^ |
| Isoxanthohumol | Prenylflavonoid | Hops ^5^ |
| 2,3-dihydroxy-3-methylvaleric acid | Hydroxy fatty acid  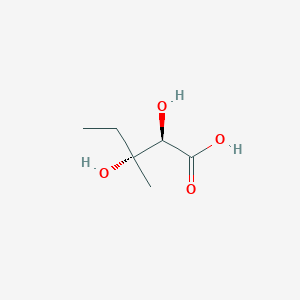 | Beer constituent; biomarker formed during the beer fermentation process ^4^. Microbial catabolism of proteins, especially branched amino acids ^6^. |
| 2-Hydroxy-3-methylbutyric acid | 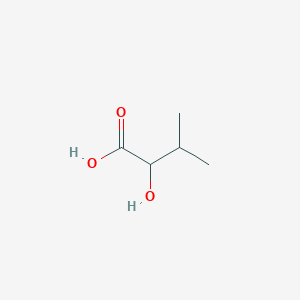 | Serotonin metabolite.^3^ |
| N-methyl tyramine sulfate (NMT sulfate) | Phenylsulfate | Compound present in barley used for beer production ^7^ |
| pyro-glutamyl proline (pGlu-pro) | Dipeptide | Produced during malting and present in beer ^7^ |
| Wine | | |
| 2-ethyl malate | Organic acid | Compound formed during fermentation of grapes ^7^ |
| iso-cohumulone and iso-ad/humulones + their major degradation products: tricyclocohumol and tricyclohumol. | Phloroglucinol derivatives (tertiary alcohol) | Compounds present in hops and their human degradation products ^7^ |
| 2-phenylethanol glucuronide | O-glucuronide | Ethanol metabolism ^4^ |
| Ethyl sulfate | Sulfuric acid monoester | Ethanol metabolism ^1^ |
| 16-Hydroxypalmitate | Long chain fatty acid | Unknown, likely endogenous response compound. |
| 2,3-butanediol | Diol | Wine compound. Formed during wine fermentation and aging ^8-10^ |
| 4-hydroxyphenylacetic acid | Phenolic acid/phenolic ester | Gut microbiota ^11,12^ |
| Gallic acid | Phenolic acid | Wine compound ^13,14^ |
| 4-O-Methylgallic acid | O-methylated phenolic acid | Human metabolite of gallic acid ^13^ |
| Gallic acid ethyl ester | Phenolic ester | Wine compound ^14^ |
| Resveratrol | Stilbenoid | Wine compound ^14^ |
| Resveratrol glucuronides | Stilbenoid O-glucuronides | Human metabolism of resveratrol ^15,16^ |
| Resveratrol sulfates | Stilbenoid sulfates | Human metabolism of resveratrol ^15^ |
| Tartaric acid | Organic acid/organic ester | Wine compound ^17^ |
| Hydroxytyrosol | Polyphenol (tyrosol) | Wine compound ^14^ |
| Ethyl-α-glucopyranoside | O-glucoside | Found in Japanese rice wine ^18^. Cannot find reports of occurrence in other types of alcoholic beverages. |

1. Plausibility:
2. Plausibility has been established for most of the compounds included in the above table except for 16-hydroxypalmitate, 2-hydroxyphytanate, and theophylline.
3. The listed biomarkers are either a) known constituents of various types of alcohol or metabolites thereof, b) originate from human metabolism of alcoholic beverages or c) represent endogenous compounds with a known response to alcohol intake.
4. Biospecimens for biomarker measurements:
5. Ethyl glucuronide has been measured in various biospecimens including urine, serum, hair, and dental tissue, whereas
6. other biomarkers related to ethanol intake have only been measured in urine (ethanol, ethyl sulfate and 2-phenylethanol glucuronide) and whole blood (phosphatidylethanols).
7. Biomarkers representing endogenous compounds have been measured in urine (5-hydroxytryptophol and 5-hydroxyindole-3-acetic acid) and serum (16-hydroxypalmitate) samples.
8. The biomarkers representing compounds originating directly from constituents of the various types of alcoholic beverages have been measured mainly in urine samples.
9. Specificity:
10. Biomarkers related to ethanol intake and metabolism are specific for alcohol intake because these compounds are only formed after ethanol intake.
11. Biomarkers of alcohol intake reflecting endogenous compounds may lack specificity. 5-hydroxytryphol and 5-hydroxyindole-3-acetic acid are for instance related to the metabolism of serotonin, but it is well established that the ratio between these two compounds change following alcohol intake although other factors may also influence serotonin metabolism. However, without any alcohol consumption the ratio between 5-hydroxytryptol and 5-hydroxyindole-3-acetic acid is usually very low (<0.01) ^19^ and considered abnormal as a result of regular ethanol intake when the ratio is above 15 pmol/nmol ^20^.
12. Considerations concerning biomarkers reflecting various types of alcohol and their specificity are included in section 1.f. below.
13. Analytical methods:
14. Most of the biomarkers have been measured by LC-MS/MS.
15. A few have only been measured by GC-MS and/or NMR (ethanol; 2,3-butanediol; 5-hydroxytryptophol).
16. Biomarkers of various types of alcohol:
17. Liquor: Biomarkers with an association to liquor consumption identified so far predominantly represent ethanol intake and thus cannot be considered specific for liquor. Wang et al. 2020 ^21^ found an association between 2-hydroxyphytanate and liquor intake but this putative biomarker is of unknown origin thus it is not possible to assess its specificity. More studies are needed to identify biomarkers of various types of liquor.
18. Beer: Relatively few studies have focused on discovering biomarkers of beer intake. The proposed biomarkers represent either compounds originating from the fermentation process or beer constituents such as hops. Although plausibility is established for most compounds the proposed biomarkers may lack sensitivity or specificity. Hops metabolites are not very stable compounds and variations in metabolite products between individuals are known ^7^. Biomarkers originating from wort or the fermentation process also lack specificity since these compounds have also been identified at baseline in intervention studies but concentrations increased following beer consumption ^7^. The biomarker, 2,3-dihydroxy-3-methylvaleric acid, formed during beer fermentation, has also been associated with wine intake ^6^ and this compound may also be formed during wine fermentation. Hitherto, the most promising biomarkers for beer are more likely combinations of seven biomarkers originating from the various raw materials used in beer production ^7^. Theophylline has also been associated with beer intake but cannot be considered specific to beer consumption because this compound is present in high levels in tea and cocoa ^22^.
19. Wine: Biomarkers of wine intake mainly reflects compounds produced during wine fermentation, small organic acids and polyphenols originating from the wine, and metabolites thereof. Various polyphenols have been associated with wine intake (gallic acid ethyl ester, 4-O-methylgallic acid, gallic acid, resveratrol, hydroxytyrosol). Some of them may lack specificity because they are also present in a variety of other foods ^14^. Gallic acid and and its metabolite 4-O-methylgallic acid have been significantly correlated with tea intake. Hydroxytyrosol is present in much higher concentrations in olives than in wine and display comparable concentrations in wine and olive oil ^14^. Gallic acid ethyl ester appears to be the most specific biomarker since it has only been reported in red wine and to a lesser extent in vinegar according to Phenol Explorer ^14^. Several organic acids were also proposed as biomarkers of wine intake. Tartaric acid is one of the main organic acids in wine but tartaric acid is also an endogenous compound present in living organisms and in various foods including grapes, citrus, tamarind, and coffee products ([www.foodb.ca](http://www.foodb.ca)). Tartaric acid is also used as an acidulant in many processed foods e.g. to impar a sour taste or control pH ^23^ and has also been proposed as a biomarker of dysbiosis ^24^. Thus, tartaric acid may lack specificity as a wine intake biomarker. 4-Hydroxyphenylacetic acid is a product of gut microbial metabolism of dietary flavonoids ^25,26^ and has also been proposed as a biomarker of dairy consumption ^27^ and is therefore not specific for wine intake.

**2. Correlations with habitual alcohol intake from FFQ studies**

| **Intake** | **Biomarker** | **Biospecimens** | **Correlation range** |
| --- | --- | --- | --- |
| Alcohol | Ethyl glucuronide | Urine, serum, hair, dental tissue | Urine: 0.2-0.6  Serum: 0.26-0.36  Hair: 0.11-0.22  Dental tissue: 0.91 |
| Alcohol | Phosphatidylethanols (PEth 16:0/16:0; PEth 18:1/18:1; PEth 16:0/18:2; PEth 16:0/18:1; PEth 18:1/16:0) | Whole blood | PEth 16:0/16:0: 0.78  PEth 18:1/18:1: 0.78  PEth 16:0/18:2: 0.71  PEth 16:0/18:1: 0.26-0.79 |
| Alcohol | 5-hydroxytryptophol and 5-hydroxyindole-3-acetic acid | Measured as the ratio between the two biomarkers in urine | No FFQ studies (24h diet recalls: 0.92) |
| Alcohol | Ethyl-α-glucopyranoside | Plasma | 0.33-0.52 |
| Beer | Humulinone | Urine | No data |
| Beer | Isoxanthohumol | Urine | No FFQ studies (intervention: 0.83) |
| Beer | 2,3-dihydroxy-3-methylvaleric acid | Urine | No data |
| Beer | N-methyl tyramine sulfate (NMT sulfate) | Urine | No data |
| Beer | pyro-glutamyl proline (pGlu-pro) | Urine | No data |
| Wine | 2-ethyl malate | Urine | No data |
| Wine | iso-cohumulone and iso-ad/humulones + their major degradation products: tricyclocohumol and tricyclohumol. | Urine | No data |
| Wine | 2-phenylethanol glucuronide | Urine | No data |
| Wine | Ethyl sulfate | Urine | No data |
| Wine | 16-Hydroxypalmitate | Serum | 0.22-0.24 |
| Wine | 2,3-butanediol | Urine | 0.22 |
| Wine | 4-hydroxyphenylacetic acid | Urine | No FFQ studies (24h diet recall: 0.19) |
| Wine | Gallic acid | Urine | FFQ: 0.48 (2-day diet records: 0.45-0.7) |
| Wine | 4-O-methylgallic acid | Urine | FFQ: 0.41 (24h-2-day diet records: 0.37-0.52) |
| Wine | Gallic acid ethyl ester | Urine | No FFQ studies (24h diet recalls: 0.51-0.69) |
| Wine | Resveratrol | Urine | 0.56-0.61 (24h diet recalls: 0.59-0.61) |
| Wine | Resveratrol glucuronides | Measured in urine as the sum of trans- and cis-resveratrol-3-O-glucuronide | 0.65 |
| Wine | Resveratrol sulfates and glucuronides | Measured in urine as the sum of trans-resveratrol-3-O-glucuronide, cis-resveratrol-4′-Oglucuronide, cis-resveratrol-3-O-glucuronide, trans-resveratrol-4′-O-sulfate, trans-resveratrol-3-O-sulfate, cis-resveratrol-4′-O-sufate, and cis-resveratrol-3-O-glucuronide | 0.9 |
| Wine | Tartaric acid | Urine | No FFQ studies (intervention: 0.92) |
| Wine | Hydroxytyrosol | Urine | No FFQ studies (24h diet recalls: 0.43) |
| Wine | Ethanol | Urine | No data |
| Wine | Theophylline | Plasma and blood, unspecified | Plasma: 0.29 |
| Wine | 2,3-dihydroxyisovalerate | Plasma and urine | Urine: 0.31  Plasma: 0.37-0.44 |

**3. Summary of studies that reported multiple markers as part of a single study (ranking)**

No studies have aimed specifically at the comparison of various biomarkers of alcohol intake. Most studies focus on discovery. The following papers report multiple biomarkers of various types of alcohol in the same study and have included data that allow for the comparison of individual biomarkers:

1. *Edmands et al.^28^:* This study reports both 4-O-methylgallic acid and gallic acid ethyl ester sulfate as biomarkers of red wine. Both biomarkers were measured in 24-hr urine. The correlation coefficients with intake are comparable for both the 24HDR (acute intake; 0.45 and 0.48 respectively) and FFQ (habitual intake; 0.41 and 0.36 respectively). The correlations are however non-significant for the FFQ study for both biomarkers (P > 0.05). The ROC AUC was higher for the 24HDR study than the FFQ study. For both studies the ROC AUC was higher for gallic acid ethyl ester sulfate than 4-O-methylgallic acid (91.9% and 84.7%).
2. *Mennen et al.^13^:* This study reports both gallic acid and 4-O-methylgallic acid as biomarkers of wine intake. Both biomarkers were measured in urine and the correlation to habitual intake (2-day dietary records) was strongest for 24h urine samples compared to spot urine samples. The correlation between habitual wine intake and gallic acid was stronger than the correlation to 4-O-methylgallic acid (24h urine: 0.7 and 0.52; spot urine: 0.45 and 0.37).
3. *Playdon et al.^29^:* This study reports both ethyl glucuronide and 2,3-butanediol as biomarkers of wine intake. Since plausibility has not been established for 2,3-butanediol, this compound is left out from further discussion. Only minor differences in the correlation to habitual intake of these two markers were reported (0.24 and 0.22). The proposed biomarker displaying the strongest association with wine intake in this study was 2,3-dihydroxyisovalerate (r = 0.31). This biomarker is not included in this summary because it does not meet the selection criteria mentioned above, i.e. not found in more than one study or too low correlation with estimated intake. A few additional biomarkers of wine intake were also mentioned in the Playdon et al. 2016 paper but the correlation coefficients were comparable to coefficients reported for ethyl glucuronide and 2,3-butanediol (0.21-0.26) and these biomarkers also did not meet the criteria for inclusion in this summary.
4. *Vázquez-Fresno et al.^30^:* This study reports both 2,3-butanediol, ethanol, ethyl glucuronide, and tartrate as possible biomarkers of wine intake although some of these compounds cannot be considered specific for wine intake. Models combining ethyl glucuronide and tartrate were also evaluated. Among the individual biomarkers, ethyl glucuronide and tartrate displayed higher AUC percentages (86.3 and 85.7% respectively) than 2,3-butanediol and ethanol in an intervention study. A combined biomarker model with both ethyl glucuronide and tartrate increased the AUC to more than 90% (intervention study: 90.7%; FFQ study: 92.4%).
5. *Zamora-Ros et al.^12^:* This study focused on polyphenols as potential biomarkers of acute wine intake and reports gallic acid, gallic acid ethyl ester, hydroxytyrosol, and resveratrol as potential biomarkers of wine consumption. Correlations to wine consumption of the previous day were strongest for gallic acid ethyl ester and resveratrol (0.69 and 0.59 respectively; 24HDR) whereas gallic acid and hydroxytyrosol had correlation coefficients between 0.4 and 0.5. A few additional polyphenols are also mentioned in this paper, but they all had correlations with habitual wine intake below 0.4 (none are included in this summary).
6. *Quifer-Rada et al.^4^:* This study uses a PLS-DA modelling approach to find potential biomarkers of habitual beer intake. Based on the VIP scores they propose two biomarkers: humulinone (VIP 4.2) and 2,3-dihydroxy-3-methylvaleric acid (VIP 2.76). Since humulinone has the highest VIP score, this biomarker is more influential in the PLS-DA model separation of samples collected during beer intake and at baseline in this intervention study.
7. *Gürdeniz et al.^7^:* This study concluded that no single biomarker was sufficient to reflect habitual beer intake and instead proposed a combined biomarker model based on: N-methyl tyramine sulfate (NMT sulfate), pGlu-pro, and 2-ethyl malate and the sum of iso-cohumulone and iso-ad/humulones + their major degradation products: tricyclocohumol and tricyclohumol.

**4. Reproducibility**

| **Biomarker** | **Half-life** | **ICC** |
| --- | --- | --- |
| Ethyl glucuronide | ~2.5 hours ^31-34^ | Serum: 0.27 ^35^  Plasma: 0.57 ^21^ |
| Phosphatidylethanols (PEth 16:0/16:0; PEth 18:1/18:1; PEth 16:0/18:2; PEth 16:0/18:1; PEth 18:1/16:0) | Mean elimination half-life values: 3.5–9.8 days for total PEth, 3.7–10.4 days for PEth 16:0/18:1, 2.7–9.5 days for PEth 16:0/18:2 and 2.3–8.4 days for PEth 16:0/20:4. ^36-39^ | No data |
| 5-hydroxytryptophol and 5-hydroxyindole-3-acetic acid | Ratio can be used to detect moderate alcohol consumption in the previous 24 hours ^20^. | No data |
| Humulinone | No data | No data |
| Isoxanthohumol | 19.9-27.5 hours ^40^ | No data |
| 2,3-dihydroxy-3-methylvaleric acid | No data | No data |
| N-methyl tyramine sulfate (NMT sulfate) | No data | No data |
| pyro-glutamyl proline (pGlu-pro) | No data | No data |
| 2-ethyl malate | No data | No data |
| iso-cohumulone and iso-ad/humulones + their major degradation products: tricyclocohumol and tricyclohumol. | Hop-derived iso-alpha-acids have a half-life in humans of about 30min ^41^. | No data |
| 2-phenylethanol glucuronide | No data | No data |
| Ethyl sulfate | Half-life, blood: 2.7-5.4 hours ^42^  92% excreted in urine after 12 hours, 100% eliminated within 36 hours ^43^  Urine T Max: 3.5-6.2 hours ^44^ | No data |
| 16-Hydroxypalmitate | No data | Serum: 0.42 ^35^ |
| 4-hydroxyphenylacetic acid | No data | Serum: 0.69 ^45^ |
| Gallic acid | 1.1-8 hour ^46,47^ | No data |
| 4-O-methylgallic acid | 1.3-4.3 hour ^46,47^ | No data |
| Gallic acid ethyl ester | No data | No data |
| Resveratrol | 9.2 hours ^48^ | No data |
| Resveratrol glucuronides | No data | No data |
| Resveratrol sulfates | No data | No data |
| Tartaric acid | No data | Serum: 0.33 ^45^ |
| Hydroxytyrosol | Plasma: 2.43 hours ^49^ | No data |
| Ethanol | 15-20min ^50^ | No data |
| Ethyl-α-glucopyranoside | No data | 0.52 ^21^ |
| 2,3-dihydroxyisovalerate | No data | 0.46 ^21^ |

**6. Conclusion:** most validated/promesing dietary biomarkers

| **Beverages** | **Biomarker** | **Biospecimen** | **Status** |
| --- | --- | --- | --- |
| Total alcohol | Ethyl glucuronide | Urine and plasma | Positive |
| Total alcohol | Peth | Plasma | Positive |
| Red wine | tartaric acid, 4-*O*-methylgallic acid, and gallic acid ethyl ester sulfate | Plasma and urine | Promising |
| Beer | Isoxanthohumol | Plasma and urine | Promising |

**Biomarkers of cereal food intake**

1. **Nature of biomarkers and their specificity**
   1. Biomarkers for cereal food intake (whole grain wheat and rye, whole grain oats, bran from wheat and rye and makers of sourdough fermented rye) belong to different chemical classes:
      1. Alkylresorcinols, ARs (total), odd chain AR homologues (C17:0, C19:0, C21:0, C23:0 and C25:0) reflecting total whole grain wheat and rye intake and/or bran intake of these cereals ^51^. The ratio C17:0/C21:0 is used to reflect the whole grain rye/whole grain wheat and rye ratio^52^.
      2. Even numbered AR homologues reflect quinoa intake ^53^.
      3. AR metabolites DHBA, DHPPA, DHPPTA reflect whole grain wheat and rye intake ^54^.
      4. Avenacoside A and B reflect whole grain oat/bran oat intake ^55^.
      5. Avenanthramides and their main metabolites, their respective dihydro-forms reflect intake of oats^56,57^.
      6. Benzoxaxinoids and some of their metabolites reflect whole grain wheat, rye and sourdough rye (2-hydroxy-N(2-hydroxyphenyl)acetaminde (HHPAA) and N-(2-hydroxyphenyl)acetamide (HPAA) ^6,58^.
   2. Plausibility (including microbial metabolites) established for most compounds:
      1. Alklresorcinols have been identified in plasma and in erythrocyte membranes and shorter homologues (C17:0 and C21:0) in urine ^52^. Intact AR homlogues have also been measured in adipose tissue ^59^. AR homologoues are specific to cereal and quinoa intake and only trace levels are from refined grains due to contamination. AR homologues also exist in low amounts in barley have been quanitifed at low levels in beer, but the by far dominating source is through consumption of whole grain/bran wheat, rye and quino ^60^.
      2. AR meabolites have been analyzed in plasma and in urine ^61,62^. They are to greatest extent specific to whole grain/bran wheat and rye intake but have also been detected after consumption of peanut, worth and beer (3,5-DHBA), and after consumption of sinapic acid and some flavonoids (3,5-DHPPA). However, the contribution of these sources are minor and it should also be noted that some methods have wrongly identified the more common 3,4 -configuration as 3,5-.
      3. Aventanthamides only exist in oats and have therefore excellent specificity ^60^.
      4. Avenacoside A and B are highly specific to oats ^60^.

Due to the lack of extensive food composition data regarding the compounds above, it cannot be excluded that they may be found to some extent in other foods.

- 1. Biospecimens for biomarker measurements and analytical methods
     1. Alkylresorcinols are measured in plasma, erythrocyte memberanes or adipose tissue by GC-MS or LC-MS ^62,63^.
     2. Alkylresorcinol metabolites are measured in plasma or in urine by UHPLC-Coularray or by LC-MS/MS^61^. Some metabolites have also been analyzed by a novel ELISA method ^64^.
     3. Avenanathramides are analyzed in plasma with UHPLC-Coularray or by LC-MS^55,56^. Avenocosides are analyzed in urine samples by LC-MS.
     4. Benzoxaxinoids and their metabolites are analyzed by LC-MS in plasma and urine ^65^.
  2. Biomarkers of cereal type and effects of processing/preparation/microbioal biotransformation
     1. Alkylresorcinols are not affected by processing or microbioal transformation ^51^.
     2. Sang et al. and Wang et al (2021) reported that gut microbiota is responsible for the conversion of avananthramides ^66^ into their dihydroforms. In this way, different AVA-metabotypes could be differentiated ^55^.

- - 1. Beckmann et al. ^67^found that some of the benzoxaxinoid metabolites were generated in the sourdough fermentation in rye. This was confirmed by Hanhineva et al. ^68^

1. **Correlations with habitual food intake (FFQ)**
   1. Alkylresorcinols
      1. In blood plasma: 0.29-0,55 (FFQ or weighed food records in plasma or serum)
      2. In urine (SPOT/OVERNIGHT/24H): Not measured in urine
   2. Alkylresorcinol metabolites
      1. In blood plasma: 0.29-0.40 for DHBA, DHPPA and DHPPTA in plasma (FFQ or weighed food records) ^51,69^.
      2. In urine (SPOT/OVERNIGHT/24H): 0.1-0.49 for DHBA, DHBA-glycine DHPPA, DHPPTA in spot urine or overnight urine and 0.20-0.71 for DHBA, DHPPA and DHPPTA for 24h urine collections ^51,69^.
   3. Avenanthramides or dehydro-avenanthramides
      1. In blood plasma: No established correlations under free-living conditions
      2. In urine: No established correlations under free-living conditions
   4. Avenacoside A and B
      1. In blood plasma: No established correlations under free-living conditions
      2. In urine: No established correlations under free-living conditions
   5. Benzoxaxinoid metabolites HHPAA and HPAA
      1. In blood plasma: No established correlations under free-living conditions
      2. In urine: 0.32-0.52 ^68^.

**Summary of whole grain intake biomarkers**

| **Precursor biomarkers** | **Biospecimens** | **Correlation range** |
| --- | --- | --- |
| Alkylresorcinols | Plasma | 0.29-0.55 |
| DHBA, DHPPA, DHPPTA | Plasma | 0.29-0.41 |
| DHBA, DHPPA, DHPPTA | Spot/overnight urine | 0.1-0.49 |
| DHBA, DHBA-glycine DHPPA, DHPPTA | 24h urine | 0.2-0.71 |
| HHPAA, HPAA | 24h urine | 0.32-0.52 |

1. **Reproducibility**
   1. Half-LIFE/LIVES (if available from intervention studies)
      1. Alkylresorcinols (45-6.6 h) ^70^
      2. DHBA and DHPPA (10-12 h) ^71,72^
      3. DHBA-glycine and DHPPTA (10-16 h) ^73^
   2. ICC (shorter-term variability (e.g., 1-week to 1-year)
      1. Alkylresorcinols in plasma (ICC=0.35-0.55, 2-3 months to 3 years) ^51^
      2. DHBA and DHPPA in plasma (ICC=0.30-0.49 2-3 months to 3 years) ^51^
      3. DHBA, DHBA-glycine, DHPPA, DHPPTA (ICC=0.29-0.73 2 weeks- 2-3 months) ^51^
2. **Dose-response (NOTE from controlled feeding/intervention studies)**
   1. AR in plasma show excellent linear dose-response ^74^
   2. AR metabolites in plasma and urine show excellent dose response ^51^
3. **Conclusion:**
   1. Alkylresorcinols in plasma have been validated and are useful biomarkers in complement to traditional dietary assessment methods.
   2. Alkylresorcinol metabolites in plasma and urine has been validated and are useful biomarkers in complement to traditioanl dietary assessment methods.
   3. Avenanathramides/dehydroavenanthramides in plasma remain to be validated but are promesing biomarkers.
   4. Avenocoside A and B in urine remain to be validated but are promesing biomarkers.

**Biomarkers of dairy intake**

1. **Nature of biomarkers and their specificity**
   1. Biomarkers for dairy intake belong to different chemical classes:
      1. **Long chain fatty acids**: pentadecanoic acid (15:0), heptadecanoic acid (17:0), Heptadecenoic acid (17:1), myristic acid (14:0), trans-9-hexadecenoic acid (trans 16:1n–7, trans palmitoleate) tetracecenoic (myristoleic) acid (14:1), methyl palmitate, N,N,N-trimethyl-5-aminovalerate, heptenedioate
      2. **Medium chain fatty acids:** capric acid (10:0), caprylate (8:0), 10-Undecenoate (11:1n-1)
      3. **Phosphatidylcholines (PCs), Lysophosphatidylcholines (LysoPCs), and cholesterol esters (CEs) incorporating long chain fatty acids**: lysophosphatidylcholine 15:0 and 17:0, cholesterol ester pentacecanoic acid (C15:0), Acyl-alkyl-phosphatidylcholines
      4. **Sugars**: lactose, galactose, galactonate
      5. **Quinolone derivatives:** 2,8-quinolinediol sulfate
      6. **Sphingomyelins:** sphingomyelin (d17:1/16:0, d18:1/15:0, d16:1/17:0), sphingomyelin (d18:1/25:0, d19:0/24:1, d20:1/23:0, d19:1/24:0), sphingomyelin (d17:2/16:0, d18:2/15:0), myristoyl sphingomyelin
      7. **Other**: phytanic acid, 4-hydroxyphenylpyruvate, picolinate, 3-hydroxyoctanoate, homostachydrine, hydantoin-5-Propionic acid, orotate, phenylacetylgylcine, 3-bromo-5-chloro-2,6-dihydroxybenzoic, quinate, equol
   2. Plausibility (including microbial metabolites):
      1. The “odd” length long chain fatty acids like pentadecanoic acid (15:0) and heptadecanoic acid (17:0) are synthesized by bacterial flora in ruminants and not produced in humans. The content of heptadecanoic acid in human subcutaneous adipose tissue is a marker of long-term milk fat intake in free-living individuals in populations with high consumption of dairy products.^75^
      2. Trans 16:1n–7 is a constituent of dairy fat.^76^
      3. Caprate, a medium-chain fatty acid, is a component of animal fat.
      4. Lactose and galactose and their metabolites (e.g., galactonate) are derived from milk. Galactose has been detected in a feeding study.^77^
      5. Quinolone derivatives, such as 2,8-quinolinediol sulfate are used as antibiotics and have been detected in milk.^78,79^
   3. Biospecimens for biomarker measurements
      1. The long chain fatty acid biomarkers have been detected in serum, plasma, dried blood spots, and adipose tissue in addition to being incorporated in erythrocytes. Caprate (10:0), PCs, and LysoPCs incorporating C15:0 and C17:0 have been detected in serum. Milk sugars have been detected in both serum and urine. Finally, quinoline derivatives have been detected in serum.
   4. Specificity:
      1. Pentadecanoic acid and heptadecanoic acid are ruminant origin metabolites that appear to primarily be biomarkers for dairy fat consumption. However, these long chain fatty acids are also found in the adipose tissue of meat. Trans 16:1n–7 is also found in dairy fat, but is a constituent of partially hydrogenated oil as well.^76^
      2. Although capric acid is found in animal fats like dairy fat, it is also widespread in plant oils and as glycerides in seed oils.
      3. Galactose is formed by hydrolysis of lactose (the major sugar in milk) during digestion. It is also found in free form in fermented milk products like yogurt and cheese, and it can also be found in some fruits and vegetables, like avocados and melons. Galactonate is a product of hepatic galactose metabolism.^80^
      4. Quinolinediols are antibiotic breakdown products, thus not specific to dairy.
   5. Analytical methods:
      1. Long chain and trans fatty acid biomarkers have been detected by GC-MS. Caprate is detected by LC-MS and GC-MS. The PC, LysoPC and CE biomarkers have been detected by LC-MS and GC-MS. Lactose, galactose and galactonate are detectable by LC-MS, GC-MS and NMR, and quinolines by LC-MS.
   6. Biomarkers of dairy type, processing, or preparation, or microbial transformation:
      1. The majority of biomarkers identified were not specific to processing methods; they have been associated with milk, butter, yogurt, cheese and ice cream.
      2. Some compounds tended to have higher correlations with higher fat dairy products, such as cheese and butter, than with other dairy products. These included, 10-undecenoate, capric acid, myristic acid (14:0), myristoleic acid (14:1), and biomarkers of the class phosphatidylcholines (PCs), Lysophosphatidylcholines (LysoPCs), and cholesterol esters (CEs) incorporating long chain fatty acids
2. **Correlations with habitual food intake (FFQ)**
   1. Long chain fatty acids and trans fatty acids
      1. Pentadecanoic acid (15:0), heptadecanoic acid (17:0), and myristoleic acid (14:1) measured in adipose had low to moderate correlations with habitual dairy intake.^81-83^ These markers as well as trans 16:1n–7 also had low to moderate correlations in blood.^76,81,83-88^
      2. While myristic acid had low correlations with dairy intake using blood samples, it had moderate correlations with dairy when measured in adipose.^76,81^
      3. Heptadecenoic acid (17:1) consistently had low correlations with dairy intake when measured in adipose and blood.^81^
      4. Methyl palmitate, N,N,N-trimethyl-5-aminovalerate, and heptenedioate had moderate correlations with habitual dairy intake measured in blood.^21,84^
      5. Pentadecanoic acid, heptadecanoic acid, and trans 16:1n–7 were also measured in red blood cells. Using this biospecimen, low to moderate correlations were observed. ^85,87^
   2. Medium chain fatty acids
      1. In serum, capric acid (10:0) and caprylate (8:0) had low to moderate correlations up to 0.26 based on FFQ data.^89,90^
   3. Phosphatidylcholines (PCs), Lysophosphatidylcholines (LysoPCs), cholesterol esters (CE)
      1. In serum, cholesterol ester incorporating C15:0 have correlated with dairy products up to ~0.46 based on 7-day food record data. PC and LysoPC correlations are unavailable (presented as beta coefficients).^91^
   4. Sugars
      1. Serum galactonate correlations range up to 0.33, and lactose correlation ranges up to 0.15 using FFQ data.^89,90,92^
   5. Quinolone derivatives
      1. 2,8-quinolinediol sulfate had a moderate correlation (0.27) with habitual dairy intake when measured in serum.^90^
   6. Sphingomyelins
      1. In blood, sphingomyelins had low to moderate correlations, ranging up to 0.25.^21,89,90,92^
   7. Other
      1. Other notable correlations include that 10-undecenoate (11:1n-1) had low to moderate correlations ranging up to 0.24, and phytanic acid measured in serum had a high correlation (0.68) with habitual intake of dairy fat.^84,89,90,93^ Additionally, in blood, phenylacetylgylcine had low to moderate correlations and quinate had a moderate correlation.^21,89,90^

| **Summary of dairy biomarkers, biospecimen sources, and range of correlation with dairy intake.** | | |
| --- | --- | --- |
| **Precursor biomarkers** | **Biospecimens** | **Correlation range** |
| Long chain fatty acids and trans fatty acids | blood | 0.00-0.39 |
| Long chain fatty acids and trans fatty acids | adipose | 0.00-0.39 |
| Long chain fatty acids and trans fatty acids | erythrocyte | 0.04-0.32 |
| Medium chain fatty acids | blood | 0.15-0.26 |
| Sugars (lactose and galactonate) | blood | 0.15-0.33 |
| 2,8-quinolinediol | blood | 0.27 |
| Sphingomyelins | blood | 0.19-0.25 |

1. **Summary of studies that reported multiple markers as part of single study (ranking)**
   1. Adipose tissue biomarker study among Costa Rican men: heptadecanoic acid (17:0) and pentadecanoic acid (15:0) had the same moderate correlation (0.31) with 2% milk, dairy, and cheese.^82^
   2. Case control study in Norway: evaluated dairy intake (dairy fat, cheese, milk, and butter) in relation to serum and adipose tissue long chain fatty acids. In adipose, the study observed pentadecanoic acid (15:0) had the strongest correlation with dairy fat (0.39), followed by myristic acid (14:0) (0.37). Also, in adipose, the researchers found moderate correlations between cheese and pentadecanoic acid (0.30), full-fat milk and heptadecanoic acid (0.27), and both myristic acid and pentadecanoic acid had moderate correlations with butter (0.21-0.23). In general, weaker correlations were observed in serum as the only correlation that was not low, was a moderate correlation (0.29) between cheese and myristoleic acid.^81^
   3. Dairy biomarker study among healthy men from Oerland military flight station: Analysis of dairy intake by both FFQ and weighed food records in relation to serum and adipose tissue 15:0 and 17:0. In adipose, pentadecanoic acid had higher correlations than heptadecanoic acid with all 3 categories (milk, cheese, and total dairy). Pentadecanoic acid had moderate correlations with cheese and total dairy (0.25-0.28), but low a correlation with milk (0.09). Moreover, the same findings were found in serum with pentadecanoic acid consistently having higher correlations with the dairy subtypes and total dairy intake. Again, a low correlation (0.09) was observed for pentadecanoic acid with milk, and moderate correlations (0.27-0.28) were observed for this compound with cheese and total dairy. Similar trends were observed when intake was measured using weighed food records, though correlations tended to be stronger. Notably, contrary to other studies, it was observed that serum 17:0 was inversely associated with milk intake.^83^
   4. PLCO cohort: Two nested studies within the Prostate, Lung, Colorectal, and Ovarian (PLCO) Cancer Screening Trial measured dairy intake measured by FFQ in relation to serum metabolites. Both studies observed correlations between 10-undecenoate (0.18-0.23).^84,89^ Guertin et al. also observed moderate correlations between butter and methyl palmitate and pentadecanoic acid.^84^ Additionally, Playdon et al. observed correlations with additional serum metabolites and ice cream, milk, and cheese, but all observed correlations were low (<0.20).^89^
   5. ATBC cohort: In In a nested case-control study within the Alpha-Tocopherol, Beta-Carotene Cancer Prevention (ATBC) study, both serum galactonate and myristoyl sphingomyelin had moderate correlations with total dairy intake assessed by FFQ.^92^
   6. MESA cohort: myristic acid (14:0), pentadecanoic acid (15:0) and trans 16:1n−7 all had low correlations (0.10-0.15) with whole fat dairy products based on FFQ.^76^
   7. CPS-II cohort: Serum 10-undecenoate (11:1n-1), 2,8-quinolinediol sulfate, caprate (10:0), and galactonate were associated with milk and butter. Two sphingomyelins and caprylate (8:0) were also associated with butter. Of these metabolites, caprate had the strongest correlation with butter (0.26) and galactonate had the highest correlation with milk intake (0.33), though all correlations observed were similar in strength.^90^
   8. HPFS cohort: The Health Professionals Follow-up Study and Nurse’s Health Studies conducted a targeted analysis of plasma and RBC 15:0, 17:0 and trans 16:1n-7 in relation to dairy fat, then associated these biomarkers with disease endpoints. The first study, published in 2014, observed that dairy fat intake (measured as percent of total fat) had the strongest correlation with pentadecanoic acid (0.22). Low correlations were observed for both heptadecanoic acid and trans 16:1n-7.^86^ The second study found both pentadecanoic acid (15:0) and trans 16:1n-7 had moderate correlations with habitual whole fat dairy intake.^87^ Moreover, in a study by Sun et al., also using data from the Nurses’ Health Study, pentadecanoic acid and trans 16:1n-7 had the strongest correlations with both habitual dairy fat intake (0.30-0.36) and dairy product intake (0.18-0.29) in both serum and erythrocytes.^85^
   9. CPS-3 cohort: In a sub-study from the Cancer Prevention Study-3 (CPS-3) cohort, heptenedioate had the highest correlation with total cheese intake (0.30). 3-bromo-5-chloro-2,6-dihydroxybenzoic and N,N,N-trimethyl-5-aminovalerate both had moderate correlations with milk intake (0.27-0.28).^21^
2. **Reproducibility**
   1. Half-life
      1. Half-life data were not collected.
   2. ICC over time (e.g., 1-week to 1-year)
      1. Mean ICC data were available for 17:0 (0.52), trans 16:1n-7 (0.57), 15:0 (0.72) and observed over 2-3 years.^94^
3. **Dose-Response (from controllled feeding/intervention studies)**
   1. No data identified
4. **Conclusion: most validated/promising dietary biomarkers**

| **Diet Exposure** | **Biomarker** | **Biospecimen** | **Status** |
| --- | --- | --- | --- |
| Total dairy | Pentadecanoic acid (15:0) | blood, adipose | positive |
| Total dairy | Trans-9-hexadecenoic acid | blood, RBC | positive |
| Total dairy | Galactonate | blood | positive |
| High fat dairy | Myristic acid (14:0) | adipose | promising |

1. **Conclusion: least validated/promising dietary biomarkers**
   1. Other observed dairy biomarkers are not recommended due to lack of specificity. For instance, caprate is not specific to dairy fat (found in animal fat) and 2,8-quinolinediol sulfate is derived from antibiotics.

**Biomarkers of meat intake**

1. **Nature of biomarkers and their specificity**
   1. Biomarkers for meat intake belong to different chemical classes:
      1. Amino acids and peptides. 3-Methylhistidine and anserine (a dipeptide of 3-methylhistidine and alanine); carnosine (a dipeptide; β-alanylhistidine); guanidinoacetate (= *N-*amidinoglycine) formed from arginine and glycine through glycine amidinotransferase; creatine (*N*-amidinosarcosine) formed by methylation of guanidinoacetate through guanidinoacetate *N*-methyltransferase; 3-dehydrocarnitine formed by oxidation of carnitine and acetylcarnitine formed by acetylation of carnitine; 4-Hydroxyproline, an amino acid abundant in collagen.
      2. Amino acid derivatives formed during heating of meat products. *N-*Nitrosoproline ^95^; MeIQx and PhIP, two heterocyclic amines formed from amino acids and creatine.
      3. Phenolic compounds formed during wood pyrolysis. Syringol sulfate, 4 methylsyringol sulfate, 4-ethylsyringol sulfate, 4-allylsyringol sulfate.

Pepper alkaloids. Piperine and piperidine are components of pepper used as an ingredient in processed meat.

- 1. Plausibility (including microbial metabolites):
     1. Most compounds are amino acids, peptides and amino acids derivatives, either present in meat, or formed during digestion of meat in the gut. All compounds have been measured in meat and meat products and plausibility is therefore established.
  2. Biospecimens for biomarker measurements:
     1. Most meat biomarkers were measured in urine. Amino acids and dipeptides have also been measured in serum or plasma, but limited information exists on correlations of concentrations in blood with food intake.
  3. Specificity
     1. Specificity is not easily assessed as food composition tables are lacking for all these compounds. All amino acids and peptides proposed as biomarkers are also found in human tissues, and more particularly muscles, and can be released in blood during tissue turnover. However, their contents in muscles may vary between species, and some of these biomarkers may be specific of the intake of a particular meat. Examples include 3-methylhistidine and anserine (a dipeptide of 3-methylhistidine and alanine). Both compounds show high concentrations in chicken and low concentrations in other meats ^96-99^. They may be good biomarkers for chicken intake. However, some particular fish species (trout, salmon) also contain these two compounds (traces have been reported in other fish species) ^100^, which may confound these associations.
     2. Creatine has also been associated with shellfish and salmon intake ^29^
     3. Specificity for some fatty acids with odd number of carbons [pentadecylic acid (15:0), margaric acid (17:0)], phytanic acid (a chlorophyll metabolite formed in the rumen) that have been associated with intake of meat from ruminants is not established. They may also arise from dairy foods ^101^. For this reason, they are not included here.
     4. Similarly, TMAO is a metabolite of choline, phospholipids, and L-carnitine, all abundant in meat and other animal products, form by the microbiota in the colon ^102,103^. However, its use as biomarker of meat intake is largely confounded by fish which naturally contains high concentrations of TMAO ^104,105^.
  4. Analytical methods:
     1. Biomarkers were analyzed by LC-MS, GC-MS or ion exchange chromatography.
     2. Some biomarkers present at low concentrations may require large sample volumes (e.g. to analyze *N*-nitrosoproline ^106^) or enrichment procedures (e.g. for PhiP purified by immunoaffinity chromatography before mass spectrometry analysis ^107^).
  5. Biomarkers of meat type, processing, or preparation, or microbial transformation:
     1. Nitroso compounds are formed by reaction of nitrite (used for coloring and preservation in processed meat) with secondary amines such as proline. They are particularly abundant in cured/heated meat ^108-110^. *N*-Nitrosoproline showed an increased level in urine after intake of processed meat ^108^. Its use as biomarker of processed meat intake has not been validated in an observational study, and specificity has not been evaluated.
     2. MeIQx and PhIP are heterocyclic aromatic amines formed by reaction of amino acids with monosaccharides.
     3. Several syringol metabolites were recently identified as biomarkers of smoked meat intake ^111^. Syringol is formed by pyrolysis of lignins when burning wood. It deposits on meat during the process of smoking. Other smoked foods like smoked fish are possible confounders.
     4. Piperine and piperettine are pepper alkaloids associated with processed meat intake (sausage, salami) ^112^. Their use as biomarkers may be confounded by other unidentified sources of pepper (e.g. table pepper).

1. **Correlations with habitual food intake (FFQ)**

Specific biomarkers:

- 1. Carnosine
     1. Carnosine in urine was associated with intake of all sorts of meat (red meat, chicken, processed meat) in free-living subjects ^104^ and in several dietary intervention studies with red meat, chicken and processed meat ^96,99,104^.
     2. Carnosine in plasma was associated with intake of red meat, beef/pork and total meat ^113^.
  2. Carnitine and carnitine derivatives
     1. Significant correlations were observed between acetylcarnitine or carnitine in urine with either red meat (r = 0.32, 0.24 resp.) or processed meat (r = 0.26, 0.22 resp.) ^114^. Acetylcarnitine was also significantly increased after consumption of all types of meat (red, processed meat, chicken) ^104^. Urinary 3-dehydrocarnitine was associated with red meat intake ^29^.
  3. 4-Hydroxyproline
     1. 4-Hydroxyproline was associated with beef burger intake in 3559 subjects from the twin UK cohort ^115^ and with total meat intake in 7012 subjects from the Tsuruoka Metabolome Cohort Study ^116^.
  4. Creatine and guanidinoacetate
     1. Evidence for association of creatine and its precursor guanidinoacetate in urine with meat intake is scant. Guanidinoacetate increased with chicken intake in both an intervention and cross-sectional study ^117^ but its specificity for chicken has not been assessed.
  5. 3-Methylhistidine
     1. Associations of 3-methylhistidine with total meat intake or red meat intake have been reported in some studies ^113,114^. However, some other publications suggest that it is a marker specific for poultry intake ^104^.

Biomarkers for specific types of meat:

- 1. Poultry
     1. High correlations were observed for 3-methylhistidine in urine and intake of poultry/chicken (r = 0.48-0.61) ^104,114,118^. Correlations with 1-methylhistidine were lower (r = 0.20-0.21). High correlations between urinary 3-methylhistidine and red meat have also been reported ^114^. 3-Methylhistidine in plasma was associated with chicken, red meat and total meat intake in German subjects whereas anserine was associated with turkey, beef/pork, and total meat intake ^113^. However, these observations are not compatible with the low content of these two compounds (3-methylhistidine, anserine) in red meat and their high content in chicken, neither with a more recent study showing a low excretion of 3-methylhistidine in urine after red or processed meat intake and high excretion after chicken intake ^104^.
  2. Cured meat
     1. *N*-Nitrosoproline level in urine is high after intake of various processed meat products and low after intake of non-processed meat products and various plant foods in an intervention study ^108^. Its use as biomarker has not been tested in free-living subjects.
  3. Smoked meat
     1. Four syringol metabolites showed higher urinary excretion in habitual consumers of smoked meat ^111^. Syringol sulfate gave the most intense signal.
  4. Processed meat
     1. Piperine and other pepper alkaloids were associated with habitual intake of sausage with moderate prediction capacity (ROC AUC 0.66) likely explained by the existence of other dietary sources ^112^.
  5. Cooked meat
     1. MeIQx concentration was high in urine from American male consumers of various processed meat products and low in urine from consumers of beef, poultry and fish ^119^. PhiP measured in urine was not correlated with intake of cooked meat (either beef, pork, chicken, bacon or sausage) from a Japanese population ^107^. However, PhiP measured in hair was strongly correlated with intake of grilled/stir fried beef, chicken and pork (r = 0.34-0.68) ^120^ suggesting that reproducibility of PhiP in blood is too low for using it as biomarker of intake.

| **Summary of meat biomarkers, biospecimen sources, and range of correlation with meat intake.** | | | |
| --- | --- | --- | --- |
| **Precursor biomarkers** | **Diet Exposure** | **Biospecimens** | **Correlation range** |
| Carnosine | Total meat | Blood | Positive (no value) |
| Acetylcarnitine | Total meat | Urine | 0.26-0.32 |
| Carnitine | Total meat | Urine | 0.22-0.24 |
| 4-Hydroxyproline | Total meat | Blood | Positive (no value) |
| Creatine | Total meat | Urine | 0.24 |
| 3-Methylhistidine | Chicken | Urine | 0.48-0.68 |
| 3-Methylhistidine | Chicken | Blood | 0.40-0.54 |
| 3-Methylhistidine | Total meat | Urine | 0.43-0.69 |
| 3-Methylhistidine | Total meat | Blood | Positive |
| Syringol derivatives | Smoked meat | Urine | Positive |
| Piperine | Sausage | Blood | 0.23 |
| Piperettine | Sausage | Blood | 0.17 |
| PhIP | Grilled/stir fried meat | Hair | 0.34-0.68 |

1. **Summary of studies that reported multiple markers as part of single study (ranking)**
   1. Very few studies did compare correlations values with meat intake for different biomarkers.
   2. In the Navy Colon Adenoma Study, slightly higher correlation for processed meat intake was observed for acetylcarnitine in urine (r=0.26) when compared with carnitine (r=0.22) ^29^. Similarly slightly higher correlation for red meat was observed for acetylcarnitine in urine (r=0.32) when compared with carnitine (r=0.24).
   3. In the EPIC cohort, when comparing correlations with sausage intake, higher values were observed for piperine in serum (r=0.23) when compared with piperettine (r=0.17)
2. **Reproducibility**
   1. Half-lives
      1. Limited data exist on half-life of meat biomarkers. 3-Methylhistidine half-life was 12.6 hrs ^97,121^. No value for other meat biomarkers could be found, but similar values are expected for other amino acids or peptides. Tmax at the peak of absorption varied between 1.5 hrs and 5 hrs for the following three biomarkers: 3-methylhistidine, anserine, and carnosine ^95,104,117,121-123^.
   2. ICC over time (e.g., 1-week to 1-year)
      1. Reproducibility was estimated for several meat biomarkers. It was found to be moderate for several compounds in urine: 3-methylhistidine (ICC=0.42), anserine (ICC=0.40), acetylcarnitine (ICC=0.48), and low for carnitine (ICC=0.16) ^124,125^.
      2. In blood, ICC values were found to be low to high for 3-methylhistidine (ICC=0.07-0.66), low to moderate for 4-hydroxyproline (ICC=0.17-0.51), moderate for acetylcarnitine (ICC=0.34-0.55) and piperine (ICC=0.55), and moderate to high for carnitine (ICC=0.52-0.77) ^126-128^.
3. **Dose-response (from controlled feeding/intervention studies)**
   1. Dose-response has been shown in a limited number of publications for 3-methylhistidine, guanidinoacetate ^97,122^.
4. **Conclusion: most validated/promising dietary biomarkers**

| **Diet Exposure** | **Biomarker** | **Biospecimen** | **Status** |
| --- | --- | --- | --- |
| Total meat | Carnosine | Urine | Promising |
| Total meat | Carnosine | Plasma | Positive |
| Total meat | 4-Hydroxyproline | Plasma/serum | Promising |
| Total meat | Acetylcarnitine | Urine | Positive |
| Total meat | Carnitine | Urine | Positive |
| Poultry/chicken | 3-Methylhistidine | Urine | Promising |
| Poultry/chicken | Anserine | Urine | Promising |
| Poultry/chicken | 3-Methylhistidine | Plasma | Promising |
| Smoked meat | Syringol sulfate | Urine | Positive |
| Sausage | Piperine | Plasma | Positive |
| Cooked meat | PhIP | Hair | Positive |

- - 1. The table mentions biospecimens where biomarkers have been tested. The same biomarkers may be equally useful in other matrices but not have been tested yet.

1. **Conclusion: least validated/promising dietary biomarkers**
   - 1. More validation studies are needed to rank biomarkers according to magnitude of correlation with meat intake. Few authors have compared the different biomarkers in a same study. For this reason, several still appear as ‘promising’.
     2. More data is needed on the specificity of some of meat intake biomarkers, in particular for 3-methylhistidine considered by some authors as a marker for total meat intake rather than a marker for chicken intake. No biomarkers for red meat have yet been identified.
     3. For processed meat, data largely come from single studies. Studies to examine possible confounding of markers for smoked meats with consumption of other smoked foods will be needed.
     4. For cooked meat, promising results were obtained with PhIP measured in hair. Limited data exists on PhIP in urine and no data in blood, as samples more widely collected in population studies.

**Biomarkers of fish and seafood intake**

1. **Nature of biomarkers and their specificity**
   1. Biomarkers for fish and seafood intake belong to different chemical classes:
      1. **Furan fatty acids:** CMPF belongs to the furan FAs, and ﬁsh is their richest source in the human diet ^129^ . Although small concentrations of CMPF have been measured in green plants, mushrooms, vegetable oils and butter ^129,130^, those foods were not associated with fasting plasma in the RCT conducted by Hanhineva et al ^131^. In addition to strong evidence of specificity in controlled studies, CMPF has been cross-sectionally associated with fish (dark, oily, and total) and shellfish intake in geographically diverse, free-living populations ^84,90,92,115,132^.
      2. **Very long chain and polyunsaturated fatty acids**: EPA (cis-20:5n-3), DHA (cis-22:6n-3), and DPA (cis-22:5n-3), are very long-chain fatty acid and Polyunsaturated fatty acids (PUFA) and are found in fish oil. EPA (cis-20:5n-3) and DHA (cis-22:6n-3) are the two most abundant omega-3s in fish oil and DPA (cis-22:5n-3)is the third most abundant LC omega-3 in fish oil ^133^, but DPA was only reported as a candidate biomarker in 1 of the reviewed studies.
      3. **Trialkyl amine oxides**: Trimethylamine N-oxide (TMAO) is generated from choline, betaine, and carnitine by microbial metabolism in the gut. Three separate controlled feeding studies found that TMAO, measured in 24-h and spot urine, increased with fish/fatty fish intake ^134-136^. One of these studies replicated the finding in a cross-sectional analysis in a subset of EPIC participants in urine and plasma ^136^.
      4. **Lysophosphatidylcholines:** 1-Docosahexaenoyl Glycero-phosphocholine is a monoglycerophospholipid in which a phosphorylcholine moiety occupies a glycerol substitution site. Lysophosphatidylcholines can have different combinations of fatty acids of varying lengths and saturation attached at the C-1 (sn-1) position. Fatty acids containing 16, 18 and 20 carbons are the most common. LysoPC(22:6(4Z,7Z,10Z,13Z,16Z,19Z)), in particular, consists of one chain of docosahexaenoic acid at the C-1 position. The docosahexaenoic acid moiety is derived from fish oils.
   2. Plausibility (including microbial metabolites):
      1. Compounds (i, ii, iv) are found in fish/fish oil
      2. TMAO (iii) is a downstream microbial metabolite of compounds found in fish

- 1. Biospecimens for biomarker measurements:
     1. Strong correlations between CMPF and seafood/fish intake were observed for serum, plasma, and urine in both fasted and non-fasted samples.
     2. Correlations for EPA, DHA, and DPA have been observed in serum and plasma (fasted and non-fasted).
     3. TMAO has been identified as a biomarker of fish intake mainly in intervention studies using urine.
     4. 1-Docosahexaenoyl Glycero-phosphocholine has been associated with total and oily fish in fasted serum/plasma and non-fasted serum.
  2. Specificity:
     1. The strongest candidate biomarkers are compounds found in high abundance in seafood or metabolites of compounds (e.g., betaine) that are abundant in seafood.
     2. Specificity is, however, not absolute since there are other foods containing CMPF, albeit in exceedingly small amounts compared to seafood ^129^; EPA, DHA, and DPA are concentrated in fish oil supplements, and TMAO is derived from choline, betaine, and carnitine which are also abundant in eggs, meat, and some plants.
     3. 1-Docosahexaenoyl Glycero-phosphocholine appears to derive primarily from ingestion of fish oil from fish but is also used as a food additive and nutritional supplement.
  3. Analytical methods:
     1. CMPF has been measured in serum, urine, and plasma using LC-MS
     2. EPA, DHA, and DPA are mainly measured in serum and plasma via LC-MS
     3. TMAO has been measured in urine using LC-MS and GC-MS
     4. 1-Docosahexaenoyl Glycero-phosphocholine measured in serum and plasma using LC-MS
  4. Biomarkers of fish or seafood type, processing, or preparation, or microbial transformation:
     1. Intervention study by Lloyd et al ^135^ only evaluated potential biomarkers of smoked salmon; however, none of the reported biomarkers were suspected of reflecting the smoking process.
     2. One cross-sectional study by Pallister et al ^115^ looked at potential biomarkers of fried fish and found one Metabolon unknown (i.e., X-11372) was higher with higher intake of fried food, including fried fish but also chips, etc.) and thus may be a marker of frying preparation.

1. **Correlations with habitual food intake (FFQ)**
   1. Fish and seafood and its metabolites:
      1. CMPF is strongly and consistently correlated with fish and seafood intake, with correlations ranging from 0.24 to 0.47 in intervention and observational human studies; correlations of similar magnitude have been observed for the two most abundant omega-3 polyunsaturated fatty acids (PUFAs) in fish oil: eicosapentaenoic acid (EPA, cis-20:5n-3) and docosahexaenoic acid (DHA, cis-22:6n-3). Another promising candidate biomarker of fish intake is TMAO, which belongs to a class of organic compounds known as trialkyl amine oxides, abundant in fish and seafood. Three separate controlled feeding studies found that TMAO, measured in 24-h and spot urine, increased with fish/fatty fish intake, with discriminatory accuracy >90%.

| **Summary of fish/seafood biomarkers, biospecimen sources, and range of correlation with fish/seafood intake.** | | |
| --- | --- | --- |
| **Biomarkers** | **Biospecimens** | **Correlation range** |
| CMPF | Serum, plasma, and FMV urine/fasted and non-fasted | 0.24-.047 |
| DHA; 22:6n3 | Serum and plasma/fasted and non-fasted | 0.26-0.37; betas 0.1-0.2 |
| EPA; 20:5n3 | Serum and plasma/fasted and non-fasted | 0.24-0.36; betas: 0.1-0.2 |
| TMAO | 24-h urine, spot urine, plasma | All 3 studies reporting fish-TMAO associations were intervention studies and did not report r’s or betas. 2 reported discriminatory accuracy as AUCs >90% |
| 1-Docosahexaenoyl Glycero-phosphocholine | Serum and plasma/fasted and non-fasted | 2 studies; r=0.24 and betas>0.1 |

1. **Summary of key studies**
   1. Anderson (2013), Cheung (2017), Hanhineva (2015), and Lloyd (2011) each had an intervention component and explored urine and/or plasma metabolites [3,5-7]. Anderson, Cheung, and Llyod identified TMAO as a biomarker of fish intake, and Hanhineva identified CMPF.
   2. The Prostate, Lung, Colorectal, and Ovarian (PLCO) Cancer Screening Trial Study ^84,92^, the TwinsUK Study ^115,132^, and in the Cancer Prevention Study-3 (CPS-3) ^90^ were observational studies that explored associations of FFQ-based seafood/fish/shellfish intake with serum, plasma and or FMV urine metabolites. Each of these studies found significant associations with 2 or more of the following metabolites: CMPF, DHA, EPA, 1-Docosahexaenoyl Glycero-phosphocholine. Guertin et al ^84^ and Pallister et al ^115^ independently identified all four metabolites, and Zheng et al ^132^, and Wang et al ^90^ identified three out of four.

1. **Reproducibility**
   1. Half-LIFE/LIVES - data not in studies reviewed
   2. 1-year ICC for serum CMPF was estimated in a sub-study of individuals from PLCO = 0.33 ^84^; additionally, several methods studies, which were not included in the review of seafood associated metabolites, reported short and longer-term ICCs for CMPF, DHA, EPA, and TMAO:

| Study | Time between sample collections | Biospecimen type | Metabolite | | | | |
| --- | --- | --- | --- | --- | --- | --- | --- |
|  |  |  | Metabolite ICC | | | | |
|  |  |  | CMPF | DHA | EPA | TMAO | |
| Agueusop 2020 ^45^ | 4 weeks | Serum | 0.93 | 0.67 | 0.59 | 0.65 |  |
| Li-Gao 2019 ^137^ | 6 months | Plasma | -- | 0.75 | -- | -- |  |
|  | 3 years | Plasma | -- | 0.58 | -- | -- |  |
| Sampson 2013 ^127^ | 1 year | Serum | 0.99 | 0.95 | 0.87 | -- |  |
| Wang 2020 ^21^ | 6 months | Plasma | 0.86 | 0.55 | -- | -- |  |

1. **Dose-response (from controlled feeding/intervention studies)**
   1. TMAO – Cheung et al identified a dose response relationship for fish intake at increasing levels of intake and serum TMAO levels in 40 healthy subjects ^136^
2. **Conclusion: most validated/promising dietary biomarkers**

| **Diet exposure** | **Biomarker** | **Biospecimen** | **Status** |
| --- | --- | --- | --- |
| Seafood, fish/shellfish | CMPF | Blood, urine | Positive |
| Seafood, fish/shellfish | DHA | Blood, adipose tissue | Promising |

- 1. CMPF - CMPF is a metabolite formed in humans from dietary furan fatty acids, which are most abundant in ﬁsh. Dietary furan fatty acids have also been measured in very low concentrations in green plants, mushrooms, vegetable oils and butter, but those foods were not associated with CMPF concentrations in fasting plasma in the RCT conducted by Hanhineva et al. Complementing strong evidence of specificity in controlled studies, CMPF has been cross-sectionally associated with intakes of fish (dark, oily, and total) and shellfish, but not other foods, in diverse, free-living populations.
  2. DHA, EPA, and DHA - The three most abundant omega-3 polyunsaturated fatty acids (PUFAs) in fish oil are eicosapentaenoic acid (EPA, cis-20:5n-3), docosahexaenoic acid (DHA, cis-22:6n-3), and docosapentaenoic acid (DPA, cis-22:5n-3). Accordingly, each has been associated with seafood, and more specifically fish, intake in human metabolomics studies. A larger number of studies have identified circulating levels of EPA and DHA, as opposed to DPA, as a candidate biomarker of fish intake
  3. TMAO - although 3 intervention studies suggest that TMAO is specific to seafood/fish intake, TMAO derives from choline, betaine, and carnitine, which are found in other animal and plant protein sources.
  4. 1-Docosahexaenoyl Glycero-phosphocholine is related to DHA, and derived from fish oils, has also been associated with total and oily fish in fasted serum/plasma and non-fasted serum

1. **Conclusion: least validated/promising dietary biomarkers**
   1. Other metabolites [i.e., DPA, lysine, anserine, 2-hydroxybutyric acid, acetylcarnitine, 2-methylbutyrylcarnitine, 3-phenylproprionate, n-6 DPA, 1,5-anhydroglucitol (1,5-AG), scyllo-inositol, 1-eicosatrienoylglycerophosphocholine, 1-oleoylglycerophosphoethanolamine, 1-arachidonoylglycerophosphoethanolamine, Ciliatine, pyroglutamine, docosahexaenoylcholine, eicosapentaenoylcholine, 1- and 3-methylhistidine, 1-linoleoylglycerophosphoethanolamine, 3,4-dihydroxyphenylacetate sulfate, Phosphatidylcholine diacyl C38:6, Phosphatidylcholine diacyl C40:6, 1-docosahexaenoylglycerol (22:6), sphingomyelin (d18:2/18:1)] were associated with seafood/fish/dark fish/oily fish/shellfish in one of the reviewed cross-sectional studies.
   2. Metabolon unknown compounds (i.e., X-5 digit number) were also reported by at least one study, with x-02269 replicating across multiple studies.

**Biomarkers of Vegetable Intake**

1. **Nature of biomarkers and their specificity**
   1. Biomarkers for vegetable intake belong to different chemical classes:
      1. **Carotenoids:** α-carotene, β-carotene, β-cryptoxanthin, carotene diol, lutein, lycopene, zeaxanthin, phytofluene, phytoene
      2. **Vitamin E and α-tocopherol**
      3. **Phenolic acids and derivatives:** caffeic acid, chlorogenic acid, *m*-coumaric acid
      4. **Flavonoids:** hesperetin, naringenin, eriodictyol, quercetin, isorhamnetin, kaempferol, apigenin
      5. **Isoflavonoids:** daidzein, genistein
      6. **Retinol**
      7. **Ascorbic acid**
      8. **Carboxylic acids and derivatives:** N-acetylalliin, betaine, alliin, *S*-allylcysteine, sulforaphane-cysteine, sulforaphane-*N*-acetyl-cysteine, citrate, n-d-acetylornithine, oxalate, cystine, ergothioneine, lysine, *S*-methylcysteine, creatine
      9. **Lipid and lipid-like molecules:** docosahexaenoate, palmitoyl-oleoyl-glycerophosphoglycerol
      10. **Other compounds:** 4-*O*-methylgallic acid, threonate, enterolactone, piperine, gallic acid, enterodiol, dithiocarbamate, sulforaphane, *N*-methyltaurine, galactarate, hippuric acid
   2. Plausibility (including microbial metabolites):
      1. *S*-methylcysteine sulfoxide and its biological precursor, *S*-methylcysteine are constituents of cruciferous vegetables.^21,138,139^
      2. Ergothioneine is component of mushrooms.^138^
      3. S-allylcysteine, and alliin are derived from gamma-glutamylcysteine in garlic.^138^ *N*-acetylalliin is also a plausible biomarker derived from alliin.^140^
      4. Lycopene is a dietary constituent of tomatoes and is significantly correlated with tomato and tomato product intake.^141^
      5. Carotenoids, including beta-cryptoxanthin and alpha-carotene, are found in red, orange, yellow fruits and vegetables as well as green leafy vegetables.^142^ They have been proposed as plausible biomarkers primarily for fruit and vegetable intake.^143,144^
   3. Biospecimens for biomarker measurements:
      1. Majority of the vegetable biomarkers were measured via plasma/serum and 24-hour urine samples.
   4. Specificity:
      1. In general, most vegetable-related metabolites are associated with multiple vegetable types
      2. Mushrooms are a primary source of ergothioneine, but this biomarker has also been detected in organ meats, beans, and oat bran.^145^
      3. Alliin, *S*-allylcysteine, and *N*-acetylalliin are possible specific biomarkers for allium vegetables.^138^ Alliin has primarily been studied as a potential biomarker of garlic intake. This compound is also present in lesser amount in olives, rice, and some citrus fruits.^146^ Similarly, while *S*-allylcysteine is found in other allium vegetables, garlic is the primary source of this compound.^147^ *N*-acetylalliin has been identified as a plausible biomarker for garlic, onion, and total allium vegetable intake.^21^
      4. Sulforaphane and *S*-methylcysteine are cruciferous vegetable constituents. However, *S*-methylcysteine is also found in beans.^21^
      5. Carotenoids, especially alpha-carotene, are plausible biomarkers of total vegetable intake. However, carotenoids are also markers of fruit intake.^148^
   5. Analytical methods:
      1. Most compounds were measured by using HPLC, LC-MS, time-resolved fluoroimmunoassay, and CE-TOF-MS.
   6. Biomarkers of vegetable type, processing, or preparation, or microbial transformation:
      1. No data were identified for specific vegetable biomarkers by processing or preparation.
2. **Correlations with habitual food intake (FFQ)**
   1. Carotenoids
      1. Blood carotenoids have a low to moderate correlation with habitual vegetable intake measured via FFQ.^21,148-156^ Specifically, alpha-carotene consistently had a moderate correlation with total vegetable intake.^148-150,153,155-157^ Alpha-carotene also had a moderate association with carrots and cooked vegetables.^149^ Other carotenoids had low to moderate associations with total vegetable intake.^148-153,155,156^ Carotene diol had a moderate association with leafy green and cruciferous vegetables, and beta-cryptoxanthin had a moderate correlation with cucumber intake.^21,154^
   2. Vitamin E and α-Tocopherol
      1. Vitamin E had a low correlation with total vegetable intake using a block FFQ.^151^
   3. Polyphenols and Phenolic acids
      1. No correlations with habitual vegetable intake using a FFQ were available. However, using two-day food records, low correlations were observed for urinary caffeic acid with potatoes, chlorogenic acid with potatoes, and *m*-coumaric acid with onion.^158^
   4. Flavonoids
      1. Low correlations were observed for urine isorhamnetin and leafy green vegetable, tomato, and pepper intake, and for quercetin and total vegetable intake.^149^
   5. Retinol
      1. In plasma, retinol had a moderate correlation with onion, leafy green vegetable, root vegetable, and non-leafy vegetable intake.^159^
   6. Vitamin C
      1. In blood, vitamin C had moderate correlations with total vegetable intake as well as with leafy green vegetable, non-leafy green vegetable, root vegetable, and onion intake.^150,159^
   7. Carboxylic acids and derivatives
      1. In blood, carboxylic acids and derivatives had low to moderate correlations with habitual vegetable intake.^21,89,116,138,160^ Specifically, alliin was moderately correlated with garlic intake.^138^ Low or moderate correlations with ergothioneine were observed for allium vegetables, asparagus, garlic, mushrooms, and total vegetables.^21,138,160^ It has been suggested the correlations of ergothioneine with vegetables other than mushrooms may be due to correlated intakes.^138^ *N*-Acetylalliin had moderate correlations with garlic, onion, and total allium vegetable intake, and *S*-allylcysteine was moderately correlated with garlic intake as well.^21,138^ *S*-Methylcysteine had low to moderate correlations with cruciferous vegetable intake.^21,89,138^ Low correlations of carboxylic acids and derivatives have been observed with light vegetables and carotenoid-rich vegetables.^116^
   8. Lipid and lipid-like molecules
      1. In plasma, the lipid and lipid-like molecules, docosahexaenoate, palmitoyl-oleoyl-glycerophosphoglycerol, had low to moderate correlation with green leafy vegetable intake.^21,89^

| **Summary of vegetable biomarkers, biospecimen sources, and range of correlation with vegetable intake.** | | | |
| --- | --- | --- | --- |
| **Precursor biomarkers** | **Biospecimens** | **Correlation range** | **Diet Variables** |
| Carotenoids (alpha-carotene, beta-carotene, lutein) | blood | 0.16-0.50 | total vegetables, total fruits and vegetables, cruciferous vegetables, root vegetables, leafy vegetables, cooked vegetables, carrot, plantain, cucumber, onion, broccoli |
| Carotenoids (other) | blood | 0.04-0.32 | total vegetables, cruciferous vegetables, leafy green vegetables, broccoli, cucumber, iceberg lettuce |
| Vitamin E | blood | 0.12 | total vegetables |
| Flavonoids (quercetin and isorhamnetin) | urine | 0.03-0.04 | total vegetables except potatoes and leafy green vegetables, tomatoes, and peppers |
| Retinol | blood | 0.21-0.28 | leafy vegetables, root vegetables, onion |
| Vitamin C | blood | 0.26-0.39 | total vegetables, leafy vegetables, root vegetables, onion |
| Carboxylic acid and derivatives (alliin, N-acetylalliin, s-allylcysteine) | blood | 0.21-0.29 | allium vegetables, onion, garlic, garlic powder |
| Carboxylic acid and derivatives (ergothioneine and s-methylcysteine) | blood | 0.15-0.28 | total vegetables, allium vegetables, cruciferous vegetables, asparagus, garlic, mushroom, onion |
| Carboxylic acid and derivatives (citrate, n-d-acetylornithine, oxalate, cystine, lysine, creatine) | blood | 0.02-0.16 | total vegetables, carotenoid-rich vegetables (pumpkin, carrot, broccoli, green leafy vegetables), other vegetables (cabbage, japanese radish, dried radish, burdock, other light vegetables) |
| Lipid and lipid-like molecules (DHA and palmitoyl-oleoyl-glycerophosphoglycerol) | blood | 0.18-0.20 | leafy greens/green leafy vegetables |

1. **Summary of key studies**
   1. European Prospective Investigation into Cancer and Nutrition (EPIC) cohort: The highest correlations with total vegetable intake were seen for plasma lutein, zeaxanthin, beta-cryptoxanthin, and beta-carotene. These correlations ranged from 0.21-0.30. Alpha-carotene had the highest correlation with root vegetable (0.39) and carrot intake (0.38). Leafy green vegetables had the highest correlation with lutein, zeaxanthin, and beta-cryptoxanthin.^141^ Additionally, a second study in participants from the EPIC cohort observed 24-hour urine quercetin had the highest correlation with onion and garlic intakes. All other observed correlations were low.^161^
   2. Validation study in Norwegian men: Total vegetable intake had the highest correlation with plasma alpha-carotene (0.39). Smaller correlations were observed with lycopene, lutein, zeaxanthin, and beta-carotene.^156^
   3. Validation study in healthy women from the Netherlands: Plasma alpha-carotene was highly correlated with total vegetable intake (0.37), followed by vitamin C (0.26) and lutein (0.26). Smaller correlations were observed for beta-carotene and beta-cryptoxanthin. The smallest correlation with vegetable intake was observed for lycopene.^150^
   4. MoBa Cohort Study: Using urinary samples for flavonoid measures and plasma samples for carotenoid measures, alpha-carotene was correlated with carrot intake and lycopene was correlated with tomato intake. Cooked vegetables had the highest correlation with alpha-carotene, followed by enterolactone and lutein. Alpha-carotene was associated with total vegetable intake, excluding potatoes.^149^
   5. Cross-sectional study at University of Minnesota: Plasma alpha-carotene had the highest correlation with total vegetable intake, followed by lutein, beta-carotene and beta-cryptoxanthin.^148^
   6. SU.VI.MAX cohort: Serum beta-carotene was highly correlated with total vegetable, root vegetable, leafy green vegetable, green bean and pea, bulb and stem vegetable (asparagus, celery, chard, fennel, garlic, leek, palm hearts, rhubarb, shallot), and flowering vegetable (artichoke, broccoli, cauliflower) intakes. Vitamin C had the highest correlation with mushroom and fruiting vegetable (avocado, bell pepper, cherry tomato, cucumber, eggplant, pumpkin, tomato, zucchini) intake. Alpha-tocopherol had low correlations with other vegetable subgroups.^162^ Additionally, a smaller study also using SU.VI.MAX participants observed that using 24-hour urine enterolactone was most highly correlated with total vegetable intake (0.31), followed by kaempferol, which had a low correlation. In contrast, using spot urine samples, the enterolactone only had a low correlation with total vegetable intake, and a moderate correlation (0.23) between enterolactone and potato intake was observed.^158^
   7. Cross-sectional study among healthy adolescents in Costa Rica: Plasma beta-cryptoxanthin was moderately correlated with cucumber intake. Beta-carotene was most strongly correlated with tomato intake, though this correlation was only moderate and followed closely by beta-carotene. Beta-carotene and beta-cryptoxanthin had equally low to moderate correlations with broccoli and vegetable salad. All carotenoid correlations with plantain and iceberg lettuce were low.^154^
   8. The MORGEN-project: Plasma lutein was most strongly correlated with total vegetable intake, followed by alpha-carotene, beta-carotene, and zeaxanthin. Beta-cryptoxanthin had a low correlation with vegetable intake in male participants. Lycopene had a low correlation with vegetable intake in males and females.^152^
   9. Validation study among employees in Copenhagen with or without a free-fruit program intervention: Total vegetable intake was most highly correlated with naringenin (0.25). Moderate correlations were also observed for citrus flavonoids (calculated as a sum of hesperetin, naringenin, and eriodictyol) and total flavonoids. Eriodictyol, quercetin, and kaempferol, hesperetin, phloretin, genistein, and daidzein individually all had low correlations with total vegetable intake.^163^
   10. Inter99 cohort study: Using overnight urine and plasma samples, alpha-carotene was most highly correlated with carrot intake followed by beta-carotene. Alpha-carotene and lutein were most strongly correlated with cruciferous vegetable intake, with correlations of 0.38 and 0.36 respectively. Alpha-carotene also had the highest correlation with total vegetable intake (0.43). Carotenoids consistently had higher correlations than flavonoids and enterolactone with total vegetable and vegetable subgroup intakes.[18] Additionally, in a studying designed to validate the Inter99 study food frequency questionnaire, plasma alpha- and beta-carotene had the highest correlations with vegetable intake in men, followed by beta-cryptoxanthin. In women, alpha-carotene had the highest correlation with vegetable intake.^155^
   11. Polyp Prevention Trail: In men, vegetable intake was most highly correlated with alpha-carotene, followed by beta-carotene. The lowest correlation with vegetable intake was seen for vitamin E.^151^
   12. Validation study among Iranian men and women: Plasma vitamin C was most highly correlated with total vegetable intake, followed by beta-carotene. Beta-carotene also had the highest correlation with root vegetables, leafy green vegetables, and dry vegetables, regardless of season. Vitamin C had the highest correlation with non-leafy green vegetables in all seasons. Onions had the highest correlation with beta-carotene in fall/winter, but in spring/summer vitamin C had a slightly higher correlation.^159^
   13. Cross-sectional study among Japanese children: Serum beta-carotene had the highest correlations with green and yellow vegetable intake for children between the ages of 10-11 and for girls ages 13-14 years. Alpha-carotene had the highest correlation with green and yellow vegetable intake for girls 13-14 years old.^143^
   14. Alpha-tocopherol, Beta-Carotene Cancer Prevention Trial (ATBC): Serum ergothioneine was most strongly correlated with total vegetable intake, followed by oxalate. N-delta-acetylornithine and threonate were equally correlated with vegetable intake, although the correlations were small.^160^
   15. Women’s Health Initiative Nutrition and Physical Activity Assessment Study ancillary feeding study: Using plasma and 24-hour urine samples, N-acetylalliin was most strongly correlated with garlic intake, followed by alliin. Sulforaphane-N-acetyl-cysteine and sulforaphane had the highest correlations with broccoli consumption.
   16. Tsuruoka Metabolome Cohort Study: Plasma threonate was most strongly correlated with carotenoid-rich vegetables (i.e., pumpkin, carrot, broccoli, green leafy vegetables, etc.). Threonate and cystine were correlated with other vegetables. All observed correlations with vegetables in this study were small.^116^
   17. New York Women’s Health Study: Serum alpha-carotene and beta-cryptoxanthin were the most strongly correlated metabolites of vegetable intake, followed by lutein. Beta-carotene had a low correlation with vegetable intake.^153^
   18. CPS-2 Nutrition Cohort study: Serum N-methyltaurine had the highest correlation with allium vegetable intake, followed by piperine, N-acetylalliin, and ergothioneine. N-acetylalliin had the highest correlation with garlic intake. S-methylcysteine sulfoxide had a moderate correlation with cruciferous vegetable consumption, while ergothioneine had a moderate correlation with mushroom intake, and N-methyltaurine had a moderate correlation with onion intake.^138^
   19. Diet Assessment Sub-study (DAS) from CPS-3 Cohort: Plasma S-methylcysteine had the highest correlation with cruciferous vegetable intake, followed by carotene diol. N-acetylalliin and alliin had the highest correlations with garlic intake. Other notable correlations included carotene diol with leafy green vegetable intake, N-methyltaurine with onion intake, ergothioneine with asparagus, and N-methyltaurine with allium vegetable intakes.^21^
   20. Shanghai Women’s Healthy Study: Using spot urine samples, no correlations were observed for isoflavonoids or the lignans, enterodiol and enterolactone and vegetables intake.^164^
   21. EPIC-InterAct Study: Using plasma samples, lutein had the highest correlation with leafy green intake. Lycopene had a moderate correlation with consumption of fruiting vegetables, and beta-carotene and lutein had low correlations with vegetable intake. A high correlation was observed for alpha carotene and root vegetables, and a moderate correlation with beta-carotene. Lutein had the highest correlation with cabbage and other vegetables, though the correlation with other vegetables was low.^144^
2. **Reproducibility**
   1. Half-life
      1. No information on half-lives of vegetable biomarkers was identified.
   2. ICC over time (e.g., 1-week to 1-year)
      1. High mean ICC values were observed for carotene diol (0.79-0.83), alpha-carotene (0.83), beta-carotene (0.84), ergothioneine (0.86), zeaxanthin (0.63), lycopene (0.63), and lutein (0.80) over 6 months to 1 year.^21,153^
      2. More moderate values were observed for 4-ethylphenyl sulfate (0.52), beta-cryptoxanthin (0.58), d-CEHC (0.48), docosahexaenoate (0.55), pentose acid (0.56), *N*-acetylalliin (0.29), N-methyltaurine (0.32), *S*-allylcysteine (0.31), *S*-methylcysteine (0.36), and pipecolate (0.32). These ICC values were also measured in two studies either over six months or one year.^21,153^
3. **Dose Response (from controlled feeding/intervention studies)**
   1. No data identified
4. **Conclusion: most validated/promising dietary biomarkers**

| **Diet Exposure** | **Biomarker** | **Biospecimen** | **Status** |
| --- | --- | --- | --- |
| Garlic | Alliin | Blood | positive |
| Garlic | *S*-Allylcysteine | Blood | positive |
| Allium vegetables | *N*-Acetylalliin | Blood | positive |
| Mushroom | Ergothioneine | Blood | positive |
| Total vegetable intake | alpha-Carotene | Blood | positive |

1. **Conclusion: least validated/promising dietary biomarkers**
   1. Other observed biomarkers with high specificity for total vegetable or subgroup vegetable intakes are not recommended to include in a dietary biomarker panel due to low correlation with habitual intake, or lack of data on correlation with habitual intake.

**Biomarkers of Legume/Pulses Intake**

1. **Nature of Biomarkers and their specificity**
   1. Biomarkers for legumes/pulses/nuts belong to different chemical classes
      - Soy: Genistein, dihydrogenistein, dihydrodaidzein, daidzein, equol, *O*-desmethylangolensin, glycitein
        - Genistein and daidzein are isoflavones and phytoestrogens abundant in soy and soy-enriched products. In pharmacokinetic studies in both animals and humans, genistein and daidzein are extensively absorbed and metabolized to their conjugated forms. Their aglycones are therefore found in very low concentrations in matrices such as plasma^165-167^.

- Equol and *O*-Desmethylangolensin (*O*-DMA) are two microbial metabolites of daidzein formed by intestinal bacteria in humans. Equol also shows estrogenic properties. Both metabolites are produced in only a fraction of humans^168,169^
- Other beans
  - Pipecolic Acid*:* (piperidine-2-carboxylic acid) is a gut microbial metabolite formed from lysine. Found in several human matrices such as urine and serum. Levels have been detected in humans after consumption of black soybeans ^170,171^
  - *S*-Methylcysteine (2-amino-3-(methylthio)propanoic acid) is a S-methyl derivative of cysteine, and has been detected in plasma and serum after the consumption of dry beans, however its specificity has not been assessed in human studies ^170^.
  - *N*-Acetylornithine has also been proposed as a marker for pulses, but its specificity is unknown ^170^.
  - Other compounds, such as kaempferol, have been proposed as a marker for pulses, however its presence after the consumption of other food sources such as coffee, tea, fruits and vegetables makes it a less reliable marker for pulse consumption ^158,172^.

1. Plausibility (including microbial metabolites) established for some compounds
   - Genistein and daidzein are known components of soy derived foods ^173^ . Pulses (such as dry beans) are known to be rich in the amino acid lysine; Pipecolic acid is one of its gut microbial metabolites, and thus is plausible as one potential marker for pulse consumption.
2. Compounds Measured (Blood? Urine?)
   - Genistein and daidzein have been detected in GC-MS, LC-MS, ELISA in plasma, serum and urine samples in intervention and cross sectional studies ^174-179^. Pipecolic Acid has been detected by LC-MS and GC-MS in serum and urine samples in intervention studies.
3. Specificity
   - Genistein and daidzein are found in high-abundance in soybean. While their presence has been reported in lower concentrations in other beans, their higher concentrations in soybean makes them highly specific for soy consumption ^180^.
   - O-DMA excretion has been weakly associated with intake of soy-derived foods. Furthermore, its presence has also been associated with consumption of red clover supplementation, suggesting a low-specificity for soy product intake ^181^.
   - Pipecolic Acid has been found in increased levels after consumption of black soybeans, however its concentration was also found to increase after intake of whole grain diets. Its specificity is therefore not established ^170^.
4. **Correlations with habitual food intake (FFQ)**

| **Summary of legume biomarkers, biospecimen sources, and range of correlation with legume intake.** | | | |
| --- | --- | --- | --- |
| **Precursor biomarkers** | **Biospecimens** | **Correlation Range** | **Diet Variables** |
| Genistein | Urine | 0.40-0.93 | Total soy and soy-enriched products, legume-based vegetables |
| Genistein | Plasma/Serum | 0.53-0.78 | Total soy and soy-enriched products, legume-based vegetables |
| Daidzein | Urine | 0.34-0.84 | Total soy and soy-enriched products, legume-based vegetables |
| Daidzein | Plasma/Urine | 0.45-0.78 | Total soy and soy-enriched products, legume-based vegetables |
| *O*-DMA | Urine | =0.39 | Soy-derived foods, red-clover supplementation |
| Pipecolic acid | Serum | =0.30 | Total whole grains, black beans, soy beans. |

1. **Summary of key studies**
   1. Cross-sectional Studies at the University of Minnesota

Urinary excretion of genistein, O-DMA and daidzein was highly correlated with consumption of soy products (tofu, soymilk, soy nuts), fruits and vegetables ^173^.

- 1. Cross-sectional study in healthy women from the Family Risk Assessment Program (FRAP) at the Fox Chase Cancer Center

Soy foot intake demonstrated higher correlations with genistein and daidzein levels in 24-h urine samples than with other fruits and vegetable intake ^182^.

- 1. Cross-sectional studies in multiethnic women in the United States
     Study in mutli-ethnic population of women found significant correlations between urinary isoflavonoid excretion and intake of soy products. ^174^ Different ethnicities demonstrated similar correlations with intake of these food products.
  2. Women’s Health Inititative Study and follow up

Habitual intake of soy products in women from the United States demonstrated high correlations and dose-response in plasma and serum levels with daidzein and genistein. ^175,179^

- 1. European Prospective Investigation into Cancer and Nutrition (EPIC) cohort:

Found highest correlations in plasma levels of daidzein and genistein with habitual soy-product intake. ^183^

1. **Reproducibility**
   1. Half-life
      1. In general, genistein has been reported to have a longer half-life than daidzein. In plasma, following consumption of a soy-based meal, genistein showed a half-life of 5.7 ± 1.3 h, while daidzein had a half-life of = 4.7 ± 1.1 h. ^184^. In a follow up study, the genistein half-life was also found to be longer than that of daidzein ^185^.
      2. In serum, following consumption of soy nuts, genistein again had a longer half-life of 10.1 h compared to 8 h for daidzein ^186^.

- After ingestion of soy foods, the complete urinary recovery of genistein and daidzein on average is complete within 24-36 h ^187^
  1. ICC over time
     1. A range of moderate ICC values were observed for genistein (0.02-0.33) over a period of 6 months to 1 year in several cross-sectional studies ^188-190^.
     2. In the same studies, moderate to high ICC mean values were observed for daidzein (0.17-0.42).

1. **Biomarkers of Legumes TYPE/ Processing/Preparation**
   1. Soybeans are consumed primarily as processed soy-derived products, which has been proposed to influence the soy food matrix that may influence the isoflavone contents. While different processing conditions has been shown to influence the metabolism and bioavailability of soy isoflavones, genistein and daidzein are considered to be good biomarkers of intake of soy ^191^.
2. **Conclusion: biomarkers to INCLUDE in a dietary biomarker panel**

| **Dietary Exposure** | **Biomarker** | **Biospecimen** | **Status** |
| --- | --- | --- | --- |
| Legumes (soy) | Genistein | Urine/Blood | Positive |
| Legumes (soy) | Daidzein | Urine/Blood | positive |

1. **Conclusion: Biomarkers NOT TO INCLUDE in a dietary biomarker panel**
   1. O-DMA , weakly associated and not specific for legumes; only detected in a fraction of humans in studies
   2. Pipecolic acid has not been evaluated individually for its specificity towards pulse consumption
   3. *S*-Methylcysteine has not been evaluated individually for its specificity for pulses
   4. *N*-Acetylornithine has not been evaluated for its specificity for pulses

**Biomarkers of Fruit intake**

1. **Nature of fruit biomarkers and their specificity**
   1. Biomarkers of fruit intake belong to different chemical classes:
      1. **Amino acids and derivatives**
         1. **Proline derivatives:** Proline betaine (stachydrine), N-methyl proline, homostachydrine, betonicine (4-hydroxyproline betaine), 3-hydroxystachydrine
         2. **Other amino acids and metabolites**: N-methylglutamate, acetylomithine, 1-aminocyclopropanecarboxylic acid, S-carboxymethyl-L-cysteine, S-methylmethionine, lysine, cystine, 5-hydroxyindoleacetate
      2. **Polyphenols**
         1. **Flavonoids:** hesperetin, naringenin, quercetin, isorhamnetin, kaempferol, eriodictyol, methyl(epi)catechin sulfate (I)**,** (−)-epicatechin, (+ )-catechin**,** daidzein, genistein
         2. **Phenolic acids**: m-coumaric acid, caffeic acid, gallic acid, 3,4-dihydroxyphenylacetic acid, homovanillic acid, 4-O-methylgallic acid
         3. **Other phenolic compounds**: phloretin, enterolactone, dihydroxyphenyl-g-valerolactone sulfate, 4-allylphenol sulfate, catechol sulfate**,** dopamine sulfate, hippuric acid, tyrosol, 3-methoxytyramine sulfate
      3. **Carotenoids and derivatives:** α-carotene, β-carotene, β-cryptoxanthin, lutein, lycopene, zeaxanthin, phytofluene, phytoene, retinol
      4. **Sugars and derivatives:**
         1. Sugars: methyl glucopyranoside, xylose
         2. Polyols: scyllo-inositol, chiro-inositol, myo-inositol, threitol, glucoheptose, galactonate, xylonate, threonate, glycerate, galactarate, tartronate
         3. Sugar metabolites: 5-hydroxymethyl-2-furoic acid**,** 5-hydroxymethyl-2-furoic acid**,** 3-carboxy-4-methyl-5-propyl-2-furanpropanoate (CMPF), ascorbic acid
      5. **Other compounds:** pyridoxate, benzoylcarnitine, 4-hydroxychlorothalonil, 1-docosahexaenoylglycerophosphocholine, creatine, 6-oxopiperidine-2-carboxylate
   2. Plausibility (including microbial metabolites):
      1. A linear dose-response was seen between citrus fruit intake and urinary excretion of proline betaine.^192,193^ Plausibility is therefore well established.
      2. The plausibility of the association between citrus fruit intake and citrus flavonoids (hesperetin and naringenin) is well established.^194^
      3. A dose dependent association was observed between total flavonoids and fruit and vegetable intake.^172,195^
      4. Xylose and threitol, a xylose metabolite, have been proposed as possible biomarkers of apple intake.^196,197^. Xylose is one of the primary forms of sugar in apples. Though not at an individual level, xylose showed a dose-response relationship with apple consumption and is described as capable of ranking individuals into categories of apple intake.^197^
      5. Phloretin, an aglycone of phloridzin, is a dietary constituent of apple.^158^
      6. Plausibility was established between banana fruit intake and 5 different biomarkers, Methoxyeugenol glucuronide (MEUG-GLUC), dopamine sulfate (DOP-S), salsolinol sulfate, xanthurenic acid, and 6-hydroxy-1-methyl-1,2,3,4-tetrahydro-β-carboline sulfate.^198^
      7. Carotenoids, including beta-cryptoxanthin and alpha-carotene are plausible biomarkers primarily for orange, yellow, and red fruits and vegetables.^143,144^
   3. Biospecimens for biomarker measurements:
      1. Majority of the fruit biomarkers were measured via 24-hour urine samples and in plasma/serum.
   4. Specificity:
      1. Specificity is not easily assessed for many compounds.
      2. Proline betaine is shown to be specific to citrus fruits ^199^.
      3. Grapefruit is known as a major source of flavanones (specifically naringenin) ^200^. Phloretin is found to be specific to apples ^201,202^.
      4. The type of carotenoid pigment can be predicted by the color of fruit or vegetable. Lycopene is specific to tomatoes and β-cryptoxanthin is specific to fruits like oranges, tangerines, and papayas ^203^.
      5. Specificity of ascorbic acid to citrus fruits and other vegetables is well known.
      6. Specificity of dopamine in banana is established ^204^.
      7. While xylose is a primary sugar in apples, it is also found in other fruits and vegetables ^197^. Additionally, xylose is produced commercially for use as a sweetener. Thus, xylose and its metabolite, threitol, lack specificity.
   5. Analytical methods:
      1. Biomarkers were measured in blood or 24-hour urine mostly by LC-MS or HPLC. A minority of biomarkers, including citrus flavonoids, were also measured in spot urine by HPLC.
   6. Biomarkers of fruit type, processing, or preparation, or microbial transformation:
      1. No data was identified for differences in biomarkers of fruit intake by processing or preparation.
2. **Correlations with habitual food intake (FFQ)**
   1. Proline and derivatives:
      1. Moderate correlations have been observed for serum proline and derivatives (e.g., N-methyl proline, betonicine, and 3-hydroxystachydrine) with citrus fruit and juice intake (0.22-0.55) measured using FFQ. Similar correlations were observed for serum stachydrine alone (0.26-0.55).^21,84,89,90,92,116^ Moderate correlations have also been observed for urinary stachydrine, *N*-methylproline, and betonicine (0.25-0.50) with citrus fruit and juice intake again measured via FFQ.^92^ Lower correlations were observed with non-citrus fruits or fruit juices (0.15-0.30).^89,92^ Notably, in a 14-day feeding study, correlations of habitual intake of citrus fruit and fruit juice intake up to 0.77 and 0.80 were observed for serum and urinary stachydrine respectively.
   2. Citrus and other flavonoids:
      1. Low to moderate correlations were observed for citrus and other flavonoids measured in urine. Hesperetin and Naringenin had consistently higher correlations with citrus fruit than non-citrus fruit, measured using FFQ (0.18-0.44) and 24-hour recall (0.13-0.60).^149,157,158,161,205,206^ Non-citrus fruit correlations for these compounds ranged from 0.13-0.19.^163,206^ The highest correlation of 0.66 was observed between urinary hesperetin and citrus fruit intake measured by four-day weighed food diary.^149^
      2. Low to moderate correlations were also observed for urinary quercetin, isorhamnetin, kaempferol, and eriodictyol using either 24-hour recall or two-day food records (0.01-0.33).^158,161,163^ These correlations did not significantly differ for citrus and non-citrus fruits.
   3. Carotenoids:
      1. Low to moderate correlations were observed for majority of the serum carotenoids with total fruit intake and specific fruits whether measured by FFQ or another method. For instance, alpha-carotene and beta-carotene correlations with total fruit or fruit juice ranged from 0.04 to 0.58 using FFQ, and a similar range was observed when using non-FFQ methods.^143,144,148,150-153,155,156,207^ Of the specific fruits assessed for these biomarkers, alpha-carotene showed highest correlations with apple intake (0.16-0.43), and beta-carotene with papaya (0.25) and melon intake (0.06-0.35).^149,154,157,159^ Beta-cryptoxanthin showed strongest correlations with citrus fruit intake measured using FFQ (0.23-0.57) and non-FFQ methods (0.26-0.51).^21,90,141,144,149,150,157^ Beta-cryptoxanthin correlations with total fruit intake were more variable (0.11-0.56 with FFQ; 0.24-0.47 with other methods).^143,144,148,150,152,153,155,207^. It was also notable that lutein and lycopene correlations tended to be higher with tomato intake than with other fruits.^141,149,157,208^
   4. Ascorbic acid:

Low to moderate correlations (0.08-0.53) were reported for serum ascorbic acid with fruit intake, including total fruit, citrus, and melon intake measured using FFQ.^92,150,159^ Similar but slightly lower correlations were observed using non-FFQ methods to assess fruit intake (0.05-0.28).^144,162^

- 1. Threitol:

A moderate correlation (0.17) was reported between threitol and apple intake in blood.^89,92^

- 1. Inositol:

Moderate correlations were observed with fruit intake and inositol biomarkers, including scyllo-inositol, chiro-inositol, and myo-inositol. Scyllo-inostiol’s and chiro-inositol’s correlations tended to be higher for citrus fruit intake with correlations of 0.30-0.46 in plasma and 0.31-0.37 in urine when intake was measured by FFQ.^84,89,90,92^ Higher correlations of these biomarkers measured in serum and urine with habitual citrus intake were observed in a 14-day feeding study (0.52-0.57).

- 1. Dopamine sulfate:

Moderate correlations were observed for plasma dopamine sulfate with banana intake measured by FFQ (0.33-0.34).^90^ In a 14-day feeding study of habitual intake urinary dopamine sulfate had stronger correlations of 0.46-0.47 with banana intake.

- 1. Phloretin:

Low to moderate correlations were observed between urinary phloretin and apple intake measured by FFQ (0.11-0.25).^149,157,205^ Stronger correlations were found with apple intake using non-FFQ methods of assessing dietary intake (0.21-0.60).^149,158,161,163,205^ Correlations with fruits other than apple were low (0.04-0.14).^158^

| **Summary of fruit biomarkers, biospecimen sources, and range of correlation with fruit intake.** | | | |
| --- | --- | --- | --- |
| **Precursor biomarkers** | **Biospecimens** | **Correlation range** | **Dietary Variable(s)** |
| Proline and derivatives (3-hydroxystachydrine, N-methyl proline, betonicine) | Blood | 0.15-0.55 | total fruit, fruit juice, apple/pear, citrus fruit and juice |
| Proline and derivatives (N-methyl proline, betonicine) | Urine | 0.25-0.50 | fruit juice, citrus fruit, |
| Citrus flavonoids | Urine | 0.18-0.44 | citrus fruit |
| Carotenoid: α- and β-carotene | Blood | 0.04-0.58 | apple, papaya, banana, total fruit, fruit juice, pineapple, tomato, melon |
| Carotenoid: β-cryptoxanthin | Blood | 0.11-0.57 | total fruit, citrus fruits, tomato, banana, papaya, orange juice |
| Carotenoid: lutein, lycopene, zeaxanthin, phytoene, and phytofluene | Blood | 0.00-0.36 | citrus fruit, tomato, total fruit, fruit juice, papaya |
| Ascorbic acid | Blood | 0.08- 0.53 | total fruit, melon |
| Threitol | Blood | 0.17 | apples, pears |
| Inositol | Blood | 0.17-0.46 | citrus, fruit juice, total fruit, apple/pear |
| Inositol | Urine | 0.29-0.37 | citrus, fruit juice |
| Dopamine sulfate | Blood | 0.33-0.34 | banana |
| Phloretin | Urine | 0.11-0.25 | apple, pears |

1. **Summary of key studies**
   1. EPIC cohort: The highest correlations were found with citrus intake and citrus flavanones (hesperetin and naringenin) and a carotenoid (β-cryptoxanthin) with citrus intake, followed by apple intake and phloretin glucuronide and dihydroxyphenyl-g-valerolactone sulfate with apple intake. Similar correlations between tomato intake and lycopene, lutein, beta-cryptoxanthin, and zeaxanthin were also found ^141,144,161,205,206^. Additionally, one study in this same cohort showed high correlation between total fruit intake and beta-cryptoxanthin.^152^
   2. CPS-II and CPS-III cohorts: the highest correlations were found between citrus fruit intake and proline derivatives (stachydrine, 3-hydroxystachydrine and N-methylproline) and chiro-inositol. This was followed by correlations between citrus intake and β -cryptoxanthin and between banana intake and dopamine sulfate ^21,90^.
   3. PLCO cohort: using serum samples, highest correlations were found between citrus fruit intake and proline derivatives in serum samples (stachydrine and betonicine), followed by scyllo-inositol, chiro-inositol and methyl glucopyranoside ^84,89^.
   4. Women's Health Initiative Nutrition and Physical Activity Assessment Study: In an ancillary feeding study replicating habitual diet, the highest correlations were found between citrus fruit intake and urinary 6-oxopiperidine-2-carboxylate and *N*-methylglutamate. High correlations between citrus intake and N-methylproline, scyllo-inositol and chiro-inositol, naringenin 7-glucuronide and β -cryptoxanthin were also reported. A high correlation between banana intake and dopamine sulfate and banana intake was also reported.
   5. Navy Adenoma Study: using both serum and urine samples, the highest correlations were observed between citrus fruit intake and proline derivatives (stachydrine and methyl proline), followed by scyllo-inositol and chiro-inositol. Moderate correlation with betonicine was also reported ^92^.
   6. MoBa (Norwegian Mother and Child Cohort Study): highest correlations between citrus intake and hesperetin (0.44) followed by β -cryptoxanthin and zeaxanthin. Correlation of phloretin with apple intake was also reported.^149^
   7. Isfahan Healthy Heart Program (IHHP): using plasma samples highest correlations were found between citrus fruit intake and vitamin C.^159^
   8. Validation study conducted in the Netherlands: b-cryptoxanthin had the strongest correlation with citrus fruit intake (0.57) and with total fruit intake (0.42). ^150^
   9. Cross-sectional study of volunteers from the University of Minnesota, alpha-carotene and beta-cryptoxanthin had the strongest correlations with total fruit intake (0.56-0.58) followed by beta-carotene.^148^
   10. Supplementation with Antioxidant Vitamins and Minerals (SU.VI.MAX) cohort: of the biomarkers observed, vitamin C had the highest correlation with fruits and fruit juices (0.34), as well as with the subcategories, pome fruits, citrus fruits, and tropical fruits.^162^ In a separate study, kaempferol had the strongest correlation with apple intake (0.45), followed by phloretin, *m*-coumaric acid, and isorhamnetin. Enterolactone had the strongest correlation with peach intake (0.24), kaempferol with red fruit (0.19), naringenin with grapefruit (0.20), and hesperetin with orange (0.35) and citrus fruit intake (0.46). Naringenin had the highest correlation with total fruit and fruit juice intake (0.35) followed by phloretin (0.30).^158^
   11. Cross-sectional study of Costa Rican men: beta-cryptoxanthin had the highest correlation with pineapple (0.24), papaya (0.41), and banana (0.23). Tomato intake had the strongest correlation with beta-carotene (0.22) followed by beta-cryptoxanthin. No significant correlations were observed for melon with any of the carotenoids assessed.^154^
   12. Fruit intervention validation study: hesperetin had the highest correlation with total fruit (0.29) and citrus fruit and juice (0.27) intake. Phloretin had the highest correlation with apple intake (0.22).^163^
   13. Inter99 cohort: alpha-carotene had the strongest correlation with apple intake (0.43) and beta-cryptoxanthin with citrus fruit (0.43). Phytoen had the highest correlation with tomatoes (0.24).^157^ In a second study of this cohort, alpha-carotene and beta-cryptoxanthin had the highest correlation with fruit intake (0.30-0.39).^155^
   14. Nurses’ Health Study (NHS) and Health Professionals Follow-up Study (HPFS): beta-carotene was observed to have the highest correlation with total fruit intake among both men and women.^207^
   15. Cross-sectional study among Japanese children: beta-cryptoxanthin had the highest correlation with total fruit intake.^143^
   16. Alpha-Tocopherol, Beta-Carotene Cancer Prevention (ATBC) Study: methyl-beta-glucopyranoside had the highest correlation with total fruit intake (0.26) followed by oxalate (0.24).^160^
   17. Tsuruoka Metabolome Cohort Study: proline betaine had the strongest correlation with fruit intake (0.27). Other observed correlations were low.^116^
   18. New York Women’s Health Study: beta-cryptoxanthin had the strongest correlation with fruit intake (0.39), followed by alpha- and beta-carotene (0.28).^153^
2. **Reproducibility**
   1. Half-Life
      1. Limited data available. Flavanones and dopamine sulfate have very short half-lives (max 2 hrs.).^194,198,209^
   2. ICC over time (e.g., 1-week to 1-year)
      1. Moderate mean ICC values were observed for proline betaine (0.35-0.50) in two studies that measured ICC over six-month or one-year periods.^21,84^ Similarly, high ICC values were observed for carotenoids (0.58-0.84) in two studies over six-month and one-year periods.^21,153^
3. **Dose-response (from controlled feeding/intervention studies)**
   1. Linear dose-response for proline betaine, flavanones and citrus fruit intake was shown ^172,193^.
   2. A linear response was also shown between xylose and apple intake ^197^.
   3. Linear dose-response was also reported between banana intake and biomarkers, methoxyeugenol glucuronide (MEUG-GLUC), dopamine sulfate (DOP-S), salsolinol sulfate, xanthurenic acid, and 6-hydroxy-1-methyl-1,2,3,4-tetrahydro-β-carboline sulfate ^198^.
4. **Conclusion: most validated/promising dietary biomarkers**

| **Diet exposure** | **Biomarker** | **Biospecimen** | **Status** |
| --- | --- | --- | --- |
| Citrus fruits | Proline betaine | blood, urine | Positive |
| Citrus fruits | Flavanones (hesperetin and naringenin) | urine | Positive |
| Citrus fruits | β-Cryptoxanthin | blood | Positive |
| Apple | Phloretin | urine | Positive |
| Tomato | Lycopene | blood | Positive |
| Banana | Dopamine sulfate | blood, urine | Positive |
| Total fruits | Inositol | blood, urine | Promising |

1. **Conclusion: least validated/promising dietary biomarkers**
   1. Other biomarkers lack specificity or did not have strong correlations with fruit intake. For instance, threitol exhibit low correlations with apple intake.

**Biomarkers of sugar intake**

**1. Nature of biomarkers and their specificity**

- 1. Biomarkers of sugar intake belong to the following chemical classes:
     1. Mono- and disaccharides (fructose and sucrose)
     2. δ ^13^C, which does not belong to a specific chemical class
  2. Plausibility (including microbial metabolites) established for most compounds:
     1. Sucrose originates directly from dietary sucrose
     2. Fructose originates directly from dietary fructose and is also a metabolite of sucrose
     3. The sum of sucrose and fructose (sucrose+fructose) is also used as a biomarker of sugar consumption instead of each compound individually
     4. δ ^13^C: Photosynthetic plants discriminate carbon isotopes when fixing carbon dioxide into organic molecules. This discrimination varies depending on photosynthetic pathways. C_4_ plants (e.g. corn, sugar cane) fix more of the heavy isotope ^13^C than C_3_ plants (most plant species). This is reflected in the ^13^C/^12^C ratio in sugars produced by these plants. The ^13^C abundance (^13^C/^12^C ratio; δ ^13^C) is changed in human biofluids and tissues upon ingestion of sugars produced by C_4_ plants. A high consumption of added sugar (e.g. sucrose from sugar cane or high fructose sirups from corn) will influence δ ^13^C.
     5. Glucose δ ^13^C: Only one diet intervention study has been performed.
     6. Alanine δ ^13^C was derived from the conversion of C13 glucose in glucose-alanine pathway. Its abundance in red blood cells or hair could indicate long-term habitual consumption of sugar.

Some metabolites have been correlated with sugar intake, but since they may reflect correlated behaviors or yet undiscovered determinants associated with sugar intake, they are not included for further scrutiny. This includes for example the association between trimethylamine N-oxide (TMAO, r = 0.23) and added sugar intake reported by Playdon et al. ^210^ originates from intestinal microbial metabolism of L-carnitine into trimethylamine which is eventually metabolized to TMAO in the liver ^211^. It has previously been suggested that sugar intake can alter the gut microbiome and the authors ascribed the association between TMAO and added sugar intake to an indirect sugar effect on TMAO due to changes in the gut microbiome ^212,213^.

In a study by Beckmann et al. ^214^ the plasma and urine metabolome response to sucrose intake in an intervention study (90 females consuming either 0, 50 or 100g sucrose in 500ml of water) and found several metabolites to be positively associated with sucrose intake. The statistical analysis is not transparent, and no p-values/correlation values are provided in the manuscript. Thus, data from this study is not included in the master table. However, the authors found clear associations between sucrose intake and sucrose and fructose concentrations in urine but not in plasma. Among the novel metabolite associations reported erythronic acid is highlighted by the authors as a potential promising biomarker of sucrose intake. Positive correlations were reported between sucrose intake and erythronic acid in both urine and plasma.

- 1. Compounds measured (SIMILARLY/DIFFERENTLY) in (BLOOD/URINE).
     1. Fructose and sucrose are only measured in urine.
     2. δ ^13^C is measured in whole blood, red blood cells, hair, and plasma. Correlation values have been reported for whole blood samples, hair, breath ^215^, and low correlation in serum ^216^.
     3. Glucose δ ^13^C has only been measured in whole blood in an intervention study.
     4. δ ^13^C alanine has been measured in red blood cells, hair, and serum ^217^.
     5. δ ^13^C proline has been measured in red blood cells ^217^.
  2. Compounds analyzed by (LC-MS/GC-MS/NMR/OTHER).
     1. Sucrose and fructose are mainly analyzed using an enzymatic assay in combination with spectrophotometric detection. UPLC-MS/MS method has also been applied ^218^ as well as GC-MS ^219^.
     2. δ ^13^C is measured using stable isotope mass spectrometry.
     3. Glucose δ ^13^C is measured using GC stable isotope mass spectrometry.
     4. δ ^13^C alanine and δ ^13^C proline are measured using GC/combustion/isotope ratio MS ^217^.
  3. Specificity (IS/IS NOT) easily assessed for the following reasons:

Sucrose and fructose are natural constituents of many foods and food products and the urinary concentration of these compounds will reflect both sugars naturally present in the foods as well as added sugar, and therefore not the intake of any specific food or food group. δ ^13^C reflects intake of sugars produced by C_4_ plants and has been used in the US as a proxy for intake of added sugars from corn and sugar cane, hence may not be applicable for other sources of sugars, e.g., sugar beet, or naturally present sugars in fruits.

- - 1. δ ^13^C alanine was associated with total sugar, added sugar, and sugar-sweetened beverages but not with intakes of corn, a source of carbon isotope that is mostly used to make corn syrup. Associations with fish, meat, dairy, or animal protein were inconsistent ^216,220^.
    2. Some metabolites (formate, isocitrate, taurine, citrulline) have also been associated with sugar intake ^221^ but as they can be produced endogenously in the body and may come from other food items, they may not be specific enough as biomarkers of sugar intake.
  1. Biomarkers of DF TYPE/PROCESSING/PREPARATION/MICROBIAL BIOTRANSFORMATION (if applicable)

Not relevant for sugar biomarkers.

**2. Correlations with habitual food intake (FFQ)**

Correlations to various types of sugar intakes have been evaluated: Intrinsic sugar (found naturally in some foods), extrinsic sugar (free sugars, usually added to processed foods and, less often, naturally occurring as free, thus rapidly available for metabolism. Sugar in milk are included here), total sugar, free sugar, sugar sweetened beverages, dietary sucrose, and dietary fructose. The correlation values do not seem to be significantly affected by the specific type of sugar to which the biomarker response is correlated.

Generally, there are very few observational studies. Only three observational studies have been performed to evaluate each of the biomarkers of sugar intake except for Glucose δ ^13^C which has not been evaluated in observational studies.

Analyses of correlation values:

Sucrose, fructose and sucrose+fructose in urine (24h urine or morning urine):

- Only one FFQ study has been conducted and did not report correlation values.
- Correlation values reported in other observational studies (mainly 24HDR) generally show poor to modest correlations with habitual sugar intake (r range 0.03-0.43). Two modest to good correlations were reported between urinary fructose and total dietary sugar intake (r=0.43) as well as between urinary sucrose+fructose and short-term intrinsic sugar consumption (r=0.32).
- The performance of urinary sucrose and fructose to indicate daily sucrose intake was reported to be comparable as urinary nitrogen to indicate protein intake in a free-living Dutch adult population ^222^.

δ 13C:

- Correlations with habitual sugar intake have only been assessed in whole blood samples.
- Only one study reporting sugar by FFQ has been conducted and reports poor to modest correlations with total added sugar and sugar sweetened beverages (r=0.28 and 0.29 respectively). For soft drinks the correlation is modest (r=0.35).
- Two other observational studies (24HDR) report correlation values. One study reports similar findings to the FFQ study with an improved modest correlation with sugar sweetened beverages (r=0.35) compared with added sugar (r=0.23) ^223^. The other study reports very poor to modest correlations for both food groups, but also finds an improved correlation with sugar sweetened beverages (r=0.21) compared with added sugar (r=0.09)^224^.

δ 13C alanine:

- may refer to isotopic pattern of glucose after conversion to alanine in glucose-alanine cycle

was checked in hair and red blood cells with long shelf-life, hence may be a promising marker to reflect a long-term habitual consumption of sugar ^217^ Its performance in serum was not as good as in the red blood cells or hair ^216^.

**SUMMARY TABLE**

| **Precursor biomarkers** | **Biospecimens** | **Correlation range*** |
| --- | --- | --- |
| Fructose | Urine | 0.10-0.43 |
| Sucrose | Urine | 0.03-0.23 |
| Sum of sucrose and fructose | Urine | 0.09-0.29 |
| δ ^13^C | Blood (fingertip) | 0.09-0.35 |
| δ ^13^C | Red blood cells | No correlation values reported |
| δ ^13^C | Hair | No correlation values reported |
| δ ^13^C | Plasma | No correlation values reported |
| δ ^13^C Glucose | Plasma | No observational studies |
| δ ^13^C Alanine | Red blood cells, hair, serum | 0.32-0.65 |

*Observational studies only, correlation range is across different definitions of dietary sugar intake, see beginning of section 2 above.

1. **Summary of studies that reported multiple markers as part of single study (ranking)**

- No studies have compared δ ^13^C and sucrose/fructose as biomarkers of sugar intake
- Two observational studies have compared sucrose and fructose in the same study and populations:

1. Intemann et al. ^225^:

- Medium term sugar intake: correlations to free (the difference between total sugar and intrinsic sugars from food groups and dairy based foods and beverages) and total sugar intake were stronger for fructose (r range 0.217-0.263) and sucrose+fructose (r range 0.252-0.276) compared with sucrose (r range 0.132-0.189). None of the biomarkers displayed significant correlations with medium term intrinsic sugar intake (P > 0.05).
- Short term sugar intake: correlations to free and total sugar intake were comparable for fructose, sucrose and sucrose+fructose. Short term sugar intake correlations to intrinsic sugar were strongest for sucrose+fructose (r=0.32) in comparison with fructose (r=0.103) and sucrose (r=0.083) alone.

1. Bingham et al. ^226^:

In this observational study no correlation values were presented, and the authors used regression coefficients to assess correlations between biomarker response and dietary consumption of sucrose and fructose. No significant or very weak associations between sugar consumption and sucrose, fructose or the urinary fructose-to-sucrose-ratio were found in obese individuals (BMI > 30). For normal-weight individuals, there were highly significant associations between dietary sugar intake and urinary sucrose (β = 0.232; P=0.001) as well as the sucrose-to-fructose ratio (β = 0.123; P< 0.001).

1. **Reproducibility**
   1. Half-LIFE/LIVES (if available from intervention studies)
      1. Sucrose: ~ 3h ^214^
      2. Fructose: 39.3±2.2min ^227^. Half-life value of fructose was determined after intake of a sucrose sweetened beverage.
      3. δ ^13^C: 50% turnover was reported to be 2.5 weeks in plasma and 5.9 weeks in red blood cells (^228^
      4. δ ^13^C alanine: no kinetics data
   2. ICC (shorter-term variability (e.g., 1-week to 1-year)
      1. Urinary sucrose and fructose: fair reproducibility (ICC 0.47 (95% 0.36-0.58) for sucrose, ICC 0.38 (95% 0.26-0.51) for fructose, ICC 0.41 (95% 0.29-0.53) for sugar). Higher within-individual variance than between-individual variance was observed ^222^.
2. **Dose-response (NOTE from controlled feeding/intervention studies)**

Dose-response has been established for sucrose and fructose in urine.

1. **Conclusion: biomarkers to INCLUDE in a dietary biomarker panel (note VALIDATED/PROMISING)**

There are only few biomarkers of sugar intake and none of them are fully validated, but fructose and sucrose have been suggested as predictive biomarkers^229^ and current data supports that^230^. Thus, all the sugar biomarkers should be included in a biomarker panel to further evaluate their performance. Analyses of δ ^13^C requires access to stable isotope mass spectrometry whereas sucrose and fructose can be analysed by LC-MS/MS.

1. **Conclusion: biomarkers NOT TO INCLUDE in a dietary biomarker panel**
   1. BIOMARKER(S): reason
   2. BIOMARKER(S): reason

**Biomarkers of tea intake**

1. **Nature of biomarkers and their specificity**
   1. Biomarkers for tea intake belong to different chemical classes:
      1. **Gallic acid and derivatives**: gallic acid, 4-O-methyl gallic acid (4-OMGA), methylgallic acid sulfate
      2. **Catechins and catechin metabolites**: catechin, epigallocatechin (EGC), epicatechin (EC), methyl(epi)catechin sulfate, dihydroxyphenyl-g-valerolactone sulfate, 5-(3', 4', 5'-Trihydroxyphenyl)-γ-valerolactone (M4), 5-(3',4'-dihydroxyphenyl)- γ-valerolactone (M6), 4-Hydroxy-(3', 4'-dihydroxyphenyl) valeric acid, 5-(3', 4'-Dihydroxyphenyl)-valeric acid, 5-(3', 4'-Dihydroxyphenyl)-valeric acid glucuronide, Dihydroxyphenyl-g-valerolactone glucuronide, hydroxyphenylvaleric acid sulfate, 4′-O-methyl-epigallocatechin, hydroxyphenylvaleric acid glucuronide
      3. **Carboxylic acids and derivatives:** theanine, 2-aminobutyrate, creatine, cysteine S-sulfate, cystine, proline betaine
      4. **Flavonoids**: quercetin, kaempferol and isorhamnetin
      5. **Other**: 2-hydroxybutyrate, galactarate, m-coumaric acid, pyrogallol glucuronide, pyrogallol sulfate, threonate, Trimethylamine-N-oxide, 3-methoxycatechol sulfate, 1,2,3-benzenetriol sulfate, catechol sulfate
   2. Plausibility (including microbial metabolites):
      1. A linear dose-response was reported between green tea intake and plasma of catechins, EGCG, ECG ^231^. Plausibility is therefore well established.
      2. The amino acid theanine is a constituent in tea.^90^
      3. Catechins and gallic acid are found in tea. Gallic acid is a known metabolite of tea. EGC is mainly formed by microbial hydrolysis of EGCG in the colon. The 4-O-methylated metabolites are common metabolites of gallic acid formed in the liver.
   3. Biospecimens for biomarker measurements:
      1. The tea biomarkers were measured in blood or 24-hour urine.
   4. Specificity:
      1. Epigallocatechin (EGC) and epicatechin (EC) appear to be derived primarily from tea ingestion but may also be obtained as supplements.
      2. Gallic acid is found in tea leaves, but like catechins, specificity is not absolute, since there are many other foods containing gallic acid, such as wine, nuts, and some berries.
      3. Theanine is an amino acid found primarily in tea, but it can also be found in some mushrooms.
   5. Analytical methods:
      1. 24-hour urine samples were analyzed mostly by LC-MS, MS or HPLC, and blood samples by capillary electrophoresis time-of-flight mass spectrometry (CE-TOF-MS) and UPLC-MS. Some biomarkers were also measured via spot urine and analyzed by HPLC-ESI-MS-MS.
   6. Biomarkers of tea type, processing, or preparation, or microbial transformation:
      1. Different biomarkers could be used to measure different types of tea. For instance, the nonprotein amino acid, theanine, was observed to have stronger correlations with caffeinated and non-herbal teas.^90^ Additionally, catechins, such as epicatechin and epigallocatechin, are found in higher concentrations in green tea and are likely better markers of green tea intake than black tea.^232^
2. **Correlations with habitual food intake (FFQ)**
   1. Gallic acid derivatives:
      1. Moderate correlations with habitual tea intake were observed for the gallic acid derivatives.^205,233^
   2. Catechins:
      1. Low to moderate correlations have been reported for epigallocatechin (EGC) and epicatechin (EC).^234^
      2. Low to moderate correlations were also observed for catechin derivatives, including methyl(epi)catechin sulfate, dihydroxyphenyl-g-valerolactone sulfate, M4, M6, 4-hydroxy-(3', 4'-dihydroxyphenyl) valeric acid, 5-(3', 4'-dihydroxyphenyl)-valeric acid, 5-(3', 4'-Dihydroxyphenyl)-valeric acid glucuronide, dihydroxyphenyl-g-valerolactone glucuronide, hydroxyphenylvaleric acid sulfate, 4′-O-methyl-epigallocatechin, and hydroxyphenylvaleric acid glucuronide.^205,234^
   3. Carboxylic acids and derivatives:
      1. Low correlations were observed with habitual tea intake for most carboxylic acids and derivatives, including 2-aminobutyrate, creatine, cysteine S-sulfate, cystine, and proline betaine.^89,116^ Only the amino acid theanine had moderate correlations.^21,89,90^ All biomarkers in the class were measured in blood.
   4. Flavonoids:
      1. A moderate correlation was observed for kaempferol and habitual tea intake in urine. Isorhamnetin and quercetin correlations were not measured using a food frequency question.^149,157^
   5. Other
      1. Moderate correlations were observed with habitual tea intake for pyrogallol glucuronide, pyrogallol sulfate, and catechol sulfate measured in urine. All other biomarkers in this class had low correlations.^21,89,116,205^

| **Summary of tea biomarkers, biospecimen sources, and range of correlation with tea intake.** | | |
| --- | --- | --- |
| **Precursor biomarkers** | **Biospecimens** | **Correlation range** |
| Gallic acid derivatives  (4-O-methyl gallic acid and methylgallic acid sulfate) | Urine | 0.38-0.50 |
| Catechins (EGC and EC) | Urine | 0.12-0.22 |
| Catechin metabolites | Urine | 0.17-0.39 |
| Theanine | Blood | 0.23-0.50 |
| Carboxylic acid and derivatives (excluding theanine) | Blood | 0.05-0.16 |
| Kaempferol | Urine | 0.41 |
| Other (pyrogallol sulfate, pyrogallol glucuronide, and catechol sulfate) | urine | 0.23-0.32 |

1. **Summary of key studies**
   1. EPIC cohort: In a study using subjects from the European Prospective Investigation on Cancer and Nutrition (EPIC) cohort, the gallic acid derivatives, methylgallic acid sulfate and 4-O-methylgallic acid had the strongest correlations with tea intake measured in urine (0.38-0.42). Catechins metabolites such as, dihydroxyphenyl-g-valerolactone sulfate, 5-(3', 4', 5'-Trihydroxyphenyl)-γ-valerolactone, and Hydroxyphenylvaleric acid glucuronide also had moderate correlations.^205^ Additionally, in a study measuring tea intake with a 24-recall, urinary gallic acid had the strongest correlation with total tea intake (0.38).^161^
   2. PLCO cohort: In a study of the Prostate, Lung, Colorectal, and Ovarian (PLCO) Cancer Screening Trial, theanine had the highest correlation with habitual tea intake (0.41).^89^
   3. Tsuruoka Metabolome Cohort Study: The strongest correlation with tea intake was observed for threonate; however, this correlation was only 0.11.^116^
   4. CPS-3 cohort: In a US Diet Validation Study using participants from the Diet Assessment Sub-study (DAS) from the Cancer Prevention Study-3 (CPS-3), theanine was observed to consistently have higher correlations with tea than 3-methoxycatechol sulfate.^21^
   5. Shanghai Cohort Study: 4′-O-methyl-epigallocatechin had the strongest correlation with green tea intake (0.31). Moderate correlations were also observed for epigallocatechin and M4 (0.21-0.22).^234^
   6. SU.VI.MAX cohort: 4-O-methylgallic acid and gallic acid had the strongest correlation with black tea intake (0.45-0.54). In contrast, m-coumaric acid had the highest correlation with herbal tea (0.21). Tea intake was assessed with a two-day dietary record.^158^
2. **Reproducibility**
   1. Half-life
      1. Half-life information was not readily available in the papers reviewed. However, half-lives of polyphenols have been summarized and range up to 28 hours.^235^
   2. ICC over time (e.g., 1-week to 1-year)
      1. Moderate to high ICC values were observed for 3-methoxycatechol sulfate (0.42) and theanine (0.60) a six months.^21^
3. **Dose-response (from controlled feeding/intervention studies)**
   1. Linear dose-response for EGCG and ECG and green tea intake has been observed ^231^.
4. **Conclusion: most validated/promising dietary biomarkers**

| **Diet Exposure** | **Biomarker** | **Biospecimen** | **Status** |
| --- | --- | --- | --- |
| Tea | 4-O-methylgallic acid | urine | positive |
| Tea | Methylgallic acid sulfate | urine | promising |
| Tea | Theanine | blood | positive |

1. **Conclusion: least validated/promising dietary biomarkers**
   1. Other biomarkers for tea intake are not recommended to include in a dietary panel due to very low correlations or lack of specificity for tea intake.

**Biomarkers of coffee intake**

1. **Nature of biomarkers and their specificity**
   1. Biomarkers for coffee intake belong to different chemical classes:
      1. **Caffeine and its metabolites** paraxanthine, theophylline, theobromine, 1-methylxanthine, 3-methylxanthine, 7-methylxanthine, 1,3,7-trimethyluric acid, 1,3-dimethyluric acid, 1,7-dimethyluric acid, 1-methyluric acid, 5-acetylamino-6-amino-3-methyluracil (AAMU).
      2. **Phenolic acids in their esterified or free form**. They include the following native esters: 5-caffeoylquinic acid (=chlorogenic acid), feruloylquinic acid and their hydrolyzed forms caffeic acid, ferulic acid and m-coumaric acid. All coffee phenolic acids can be further metabolized by the microbiota (dihydrocaffeic acid, dihydroferulic acid) and O-methylated (isoferulic acid) or conjugated to sulfate esters and glucuronides in the tissues.
      3. **Organic acids** (quinic acid and hippuric acid). Quinic acid is formed by hydrolysis of chlorogenic acid in the colon. It is further metabolized by the microbiota into benzoic acid or 4-hydroxybenzoic acid, which is conjugated to glycine in human tissues to form hippuric acid and 4-hydroxybenzoic acid.
      4. **Trigonelline and its metabolites** nicotinic acid and N-methylpyridinium.
      5. **Roasting compounds**: diketopiperazines [cyclo(isoleucyl-prolyl), cyclo(leucyl-prolyl), cyclo(prolyl-valyl)] level (formed by conjugation of 2-furoic acid with glycine), small phenols (catechol, guaiacol, ethylcatechol, 3-methylcatechol present as sulfate esters or glucuronides) and other pyrolysis products formed from proteins during coffee bean roasting.
      6. **Other compounds**: 3-hydroxypyridine, 2,3-dihydroxypyridine, atractyligenin, 7-hydroxy-4-(methoxymethyl)coumarin, ethyl 3-mercaptopropanoic acid, p-hydroxyphenyllactic acid.
   2. Plausibility (including microbial metabolites):
      1. All compounds are known constituents of coffee brews. Plausibility is therefore well established.

- 1. Biospecimens for biomarker measurements
     1. Most compounds were measured indifferently in blood or urine.
  2. Specificity:
     1. Specificity is not easily assessed because food composition tables are lacking for many coffee compounds with the exception of phenolic acids ^236^ and caffeine ^237^.
     2. No major dietary sources apart from coffee are known for trigonelline, chlorogenic acid, quinic acid and atractyligenin, which appear to be most specific for coffee.
  3. Analytical methods:
     1. Most compounds have been measured by LC-MS.
  4. Biomarkers of dairy type, processing, or preparation, or microbial transformation:
     1. Different biomarkers could be used in combination to measure exposure to different types of coffee, for example using the trigonelline to caffeine ratio to estimate intake of caffeinated and decaffeinated coffee. Concentrations in caffeine and its metabolites were much higher in caffeinated coffee consumers than in decaffeinated consumers (based on FFQ data), in contrast to other coffee biomarkers (catechol sulfate, N-(2-furoyl)glycine, quinic acid, trigonelline) which showed similar concentrations in both types of consumers. Expectedly, caffeine and caffeine metabolite concentrations were not correlated with intake of decaffeinated coffee, but were well correlated with caffeinated coffee or total coffee intake ^238^.
     2. Combinations of compounds formed during coffee roasting and compounds relatively stable during roasting (e.g. cyclo(isoleucylprolyl)/trigonelline or catechol/trigonelline) could be used to compare intake of coffee differing in their levels of roasting ^239^.
     3. Some compounds may also be of interest to evaluate exposure to different coffee varieties. N-Caffeoyltryptophan is characteristic of Robusta varieties ^240^, however its measurement in humans has not yet been reported.

1. **Correlations with habitual food intake (FFQ)**
   1. Caffeine and its metabolites:
      1. In blood, low to moderate correlations with habitual food intake were observed for caffeine and its metabolites. No difference in correlation values could be seen between caffeine and its metabolites.
      2. In urine (spot or overnight), measured correlations were in the same range when compared with those measured in blood ^160^.
   2. Phenolic acids and its metabolites:
      1. Only limited data is available with low (dihydroferulic acid 4-sulfate) to moderate (isoferulic acid) correlations with habitual coffee intake. Moderate to high correlations (r = 0.18-0.62) were observed between phenolic acid concentrations in urine and acute coffee intake.
   3. Organic acids (quinic acid and hippuric acid):
      1. For quinic acid, high correlations with habitual coffee intake have been reported (up to 0.65), often higher than for caffeine. Correlations with hippuric acid are low or very low.
   4. Trigonelline:
      1. Correlation values are similar to those of quinic acid in blood and urine.
   5. Roasting compounds:
      1. Correlation values are similar to those observed for phenolic acids or trigonelline.
      2. Only N-(2-furoyl)glycine has been measured in urine. It shows moderate correlation with coffee intake.

| **Summary of dairy biomarkers, biospecimen sources, and range of correlation with dairy intake.** | | |
| --- | --- | --- |
| **Precursor biomarkers** | **Biospecimens** | **Correlation range** |
| Caffeine and metabolites | Blood | 0.00-0.47 |
| Caffeine and metabolites | Urine | 0.25-0.42 |
| Phenolic acids and metabolites | Blood | 0.00-0.65 |
| Phenolic acids and metabolites | Urine | 0.26-0.43 |
| Trigonelline | Blood | 0.12-0.61 |
| Trigonelline | Urine | 0.38 |
| Roasting compounds | Blood | 0-0.57 |
| Roasting compounds | Urine | 0.28 |
| Quinic acid | Blood | 0.02-0.77 |
| Quinic acid | Urine | 0.43 |
| Hippuric acid | Blood | 0.00-0.13 |
| Hippuric acid | Urine | 0.28 |

1. **Summary of studies that reported multiple markers as part of single study (ranking)**
   - 1. PLCO cohort. In the Prostate, Lung, Colorectal and Ovarian Cancer (PLCO) cohort, using serum samples, highest correlation were observed for trigonelline and quinic acid ^238^. Same results were obtained for caffeinated and decaffeinated coffee.
     2. Navy Colon Adenoma Study. Using serum samples, highest correlations were observed for quinic acid followed by caffeine and caffeine metabolites ^160^. More modest correlations were observed for catechol sulfate and N-(2-furoyl)glycine. In urine, highest correlation were found for quinic acid, followed by caffeine and caffeine metabolites and trigonelline.
     3. EPIC cohort. Using serum samples, highest correlations were observed for trigonelline followed by caffeine metabolites, diketopiperazine, quinic acid and catechol sulfate ^239^. When countries were considered individually, top biomarkers were cyclo(isoleucyl-prolyl) and trigonelline in France, trigonelline and 5-acetylamino-6-amino-3-methyluracil (AAMU) in Germany, Cyclo(isoleucyl-prolyl) in Italy, and quinic acid and Cyclo(isoleucyl-prolyl) in Greece. These differences may reflect the different types of coffee brews consumed in these countries.
     4. VIP (Vasterbotten Intervention Programme) study. Using plasma samples, low correlations between trigonelline and intake of boiled coffee were found ^241^. Higher correlations were observed between quinic acid and caffeine and its metabolites with intake of filtered coffee.
     5. Tsuruoka Metabolome Cohort Study (TMCS). Quinic acid showed the highest correlation with coffee intake, when compared with trigonelline ^116^. Low correlation was observed with hippuric acid.
2. **Reproducibility**
   1. Half-life

Limited data available. Most coffee biomarkers have short half-lives (max 5 hrs).

- 1. ICC over time (e.g., 1-week to 1-year)
     1. High mean ICC values were observed for trigonelline (0.70), caffeine (0.60) and most caffeine metabolites (0.60-0.97). More moderate values were observed for phenolic compounds such as caffeic acid sulfate (0.38) and catechol sulfate (0.46), as well as hippuric acid (0.49) and N-(2-Furoyl)glycine (0.53). Good reproducibility values for most coffee compounds are explained by the frequent intake of coffee by coffee consumers.

1. **Dose-Response (from controlled feeding/intervention studies)**
   1. No data identified
2. **Conclusion: most validated/promising dietary biomarkers**

| **Diet Exposure** | **Biomarker** | **Biospecimen** | **Status** |
| --- | --- | --- | --- |
| Coffee | Trigonelline | Plasma, serum | Positive |
| Coffee | Trigonelline | Urine | Promising |
| Coffee | Quinic acid | Plasma, serum | Positive |
| Coffee | Quinic acid | Urine | Promising |
| Coffee | Caffeic acid sulfate | Urine | Positive |
| Caffeinated/decafeinated coffee | Caffeine/trigonelline | Plasma, serum, urine | Promising |
| Coffee roasting | Cyclo(isoleucylprolyl)/trigonelline | Plasma, serum, urine | Promising |
| Coffee roasting | N-(2-furoyl)glycine/trigonelline | Plasma, serum, urine | Promising |
| Coffee roasting | Nicotinic acid/trigonelline | Urine | Promising |

1. **Conclusion: least validated/promising dietary biomarkers**
   1. Other coffee compounds with high specificity for coffee are not easily measured because of limited gut absorption and low concentration (chlorogenic acid, feruloylquinic acid) or because of lack of commercial chemical standards (e.g. sulfate esters and glucuronides for phenolic compounds).

**Biomarkers of fats and oils intake**

1. **Nature of biomarkers and their specificity**
   1. Biomarkers for intakes of fats and oils belong to different chemical classes:
      1. **Long chain fatty acids** including pentadecanoic acid or Pentadecylic acid (15:0), heptadecanoic acid or Margaric acid (17:0), myristic acid (14:0) ^76,81,84,91,93,115,242^.
      2. **Very long chain fatty acids** like EPA (cis-20:5n-3), DHA (cis-22:6n-3), and DPA (cis-22:5n-3) and other n3-Polyunsaturated fatty acids (n3-PUFA). EPA (cis-20:5n-3) and DHA (cis-22:6n-3) are the two most abundant omega-3s in fish oil and DPA (cis-22:5n-3) is the third most abundant LC omega-3 in fish oil ^243^.
      3. **Trans fatty acids** such as Palmitelaidic acid (or trans 16:1n–7) which is the trans isomer of palmitoleic acid
      4. The amino acids Creatine, N-Acetylglutamine and N-Acetyltyrosine in overnight urine ^92^.
      5. **Total polyphenol compounds (including gallic acid and catechin)** in first morning urine ^244^.
   2. Plausibility (including microbial metabolites):
      1. Pentadecanoic acid (15:0) and heptadecanoic acid (17:0) are synthesized by bacterial flora in ruminants and not produced in humans. Very long chain fatty acids (e.g. EPA, DHA, DPA and other n3-Polyunsaturated fatty acids) are found in fish oil.
   3. Biospecimens for biomarker measurements:
      1. The long chain fatty acid biomarkers have been detected in serum, plasma, dried blood spots and adipose tissue in addition to being incorporated in erythrocytes. Correlations for EPA, DHA, and DPA have been observed in serum and plasma (fasted and non-fasted).
   4. Specificity:
      1. Pentadecanoic acid and heptadecanoic acid are ruminant origin metabolites specific to dairy fat consumption.
      2. The strongest candidate biomarkers are metabolites of compounds that are abundant in seafood oils.
      3. Specificity is, however, not absolute since there are other foods containing; EPA, DHA, and DPA are concentrated in fish oil supplements.
   5. Analytical methods:
      1. Long chain and trans fatty acid biomarkers have been detected by GC-MS and GC-Flame ionization detection (FID).
      2. Amino acids were determined by means of untargeted ultrahigh-performance liquid chromatography mass spectrometry, ultra-high performance liquid chromatography tandem mass spectrometry, and gas chromatography mass spectrometry.
      3. A rapid Folin–Ciocalteu method was used to determine Total Polyphenol Compounds in urine samples using Oasis® MAX 96-well plate cartridges for solid phase extraction.
   6. Biomarkers of fat and oil type, processing, or preparation, or microbial transformation:
      1. Fats and oils are produced by extracting the lipid fraction from foods such as animal products (e.g. fish, meat, dairy) and plants (e.g. vegetables, fruits and grains). Fats and oils are composed of molecules known as triglycerides, which are esters composed of three fatty acid units linked to glycerol. An increase in the percentage of shorter-chain fatty acids and/or unsaturated fatty acids lowers the melting point of a fat or oil. Double bonds present in unsaturated triglycerides can be hydrogenated to convert oils (liquid) into margarine (solid); as such, vegetable oils (canola, corn, soybean) are industrially transformed into margarine and cooking fats. In the preparation of margarine, for example, partially hydrogenated oils are mixed with water, salt, and nonfat dry milk, along with flavoring agents, coloring agents, and vitamins, which are added to approximate the look, taste, and nutrition of butter. During the hydrogenation of vegetable oils, an isomerization reaction occurs that produces trans fatty acids. These trans fatty acids are more typical for the processing rather than the oils itself.
      2. Fats and oils also contain other compounds such as polyphenols and/or amino acids; however, since fats and oils are extracted from animal and plant foods, most of the compounds found in fats and oils are also found in the foods that they are derived from and as such not specific for fats and oils.
2. **Correlations with habitual food intake (FFQ)**
   1. Medium-chain fatty acids (MCFA; aliphatic tails of 6 to 12 carbons)

A weak correlation of 0.23 was reported for the MCFA 10-Undecenoic acid (11:1n1) in relation to fats and oils.

- 1. Long chain fatty acids (aliphatic tails of 13 to 21 carbons) and trans fatty acids

Weak to moderate correlations up to ~0.19 (for 17:1), ~0.40 (for 17:0), ~0.47 (for 15:0) and 0.22 (for trans 16:1n-7) were reported for serum/plasma LCFA.

- 1. Very long chain fatty acids (VLCFA; aliphatic tails of 22 or more carbons)

Weak to moderate correlations, ranging from 0.24 (for DPA) to 0.44 (for EPA), were reported for serum/plasma VLCFA

| **Summary of fats and oils biomarkers, biospecimen sources, and range of correlation with fats/oils intake.** | | |
| --- | --- | --- |
| **Biomarker** | **Biospecimens** | **Correlation range** |
| 10-Undecenoic acid (11:1n1) | Blood | 0.23 |
| cis-Heptadecenoic acids (cis-17:1) | Blood | 0.11-0.19 |
| cis-Tetradecenoic acids (cis-14:1) | Blood | 0.03-0.12 |
| Creatine | Urine | 0.30 |
| DHA (cis-22:6n-3) | Blood | 0.25-0.36 |
| DPA (cis-22:5n-3) | Blood | 0.24-0.38 |
| EPA (cis-20:5n-3) | Blood | 0.29-0.44 |
| Fatty acids, n3-polyunsaturated (n3-PUFA) | Blood | 0.27-0.41 |
| Margaric acid (17:0) | Blood | 0.40 |
| Methyl palmitic acid (C17H34O2) | Blood | 0.26 |
| Myristic acid (14:0) | Blood | -0.92-0.13 |
| N-Acetylglutamine | Urine | 0.25 |
| N-Acetyltyrosine | Urine | 0.26 |
| Palmitelaidic acid (trans-16:1n-7) | Blood | 0.13-0.22 |
| Pentadecylic acid (15:0) | Blood | 0.0-0.47 |
| Phytanic acid (branched chain fatty acid C20H40O2) | Blood | 0.22 |
| Polyphenols | Urine | -0.09 |

1. **Summary of key studies**
   1. EPIC-Oxford cohort: Allen (2008) showed, using data from the cross-sectional EPIC-Oxford study that a diet high in fat from dairy products is associated with increased plasma phytanic acid concentration ^93^.
   2. Cohort of men living in the municipality of Uppsala, Sweden: Smedman (1999) suggested that 15:0 in serum can be used as a marker for intake of milk fat ^91^.
   3. MESA cohort: de Oliveira Otto (2013) also identified plasma phospholipid 15:0 as a biomarker of dairy fat in a multiethnic cohort of 2837 US adults ^242^.
   4. MESA cohort: (Mozaffarian 2013), myristic acid (14:0), pentadecanoic acid (15:0) and trans 16:1n−7 were associated with whole fat dairy products based on FFQ ^76^.
   5. TWINS UK cohort: Pallister (2016) evaluated dairy & fat associations with fasting serum and plasma metabolites. 10-Undecenoic acid (11:1n1), 14:0 and 15:0 were associated with butter intake ^115^.
   6. Case control study in Norway: evaluated dairy intake in relation to serum and adipose tissue long chain fatty acids, finding several associations with heptadecanoic, pentadecanoic, and myristic acids (Biong, 2006) ^81^
   7. The cross-sectional Nunavik Inuit adults Health Survey: Lucas (2010) indicates that RBC n-3 LC-PUFA levels are important biomarkers for marine mammal fat intake ^243^.
   8. PLCO cohort: In a nested study, dairy & fat intake by FFQ was evaluated in relation to serum metabolites. Observations that replicated included correlations with 10-Undecenoate (11:1n–1). Guertin (2014) ^84^
   9. PREDIMED trial: intake of oils was negatively correlated with intakes of total polyphenols excreted in spot urine samples. (Medina-Remón, 2009) ^244^
2. **Reproducibility**
   1. Half-life
      1. Half-life data were not identified
   2. ICC over time
      1. Reproducibility over time estimates not identified
3. **Dose-response (from controlled feeding/intervention studies)**
   1. No data identified
4. **Biomarkers of DF TYPE/PROCESSING/PREPARATION**
   1. The biomarkers identified were not specific to processing methods; they have been associated with plant, seafood oils and butter.
5. **Conclusion: most validated/promising dietary biomarkers**

| **Diet exposure** | **Biomarker** | **Biospecimen** | **Status** |
| --- | --- | --- | --- |
| Fats and oils: Butter, Margarine, Meat fat, Mayonnaise, Salad dressing, Oil used for cooking, Shortening | Creatine | Urine | Promising |
|  | N-Acetylglutamine | Urine | Promising |
|  | N-Acetyltyrosine | Urine | Promising |
| Oils from fish and marine mammals | DHA (cis-22:6n-3) | Blood | Positive |
| Oils from fish and marine mammals | DPA (cis-22:5n-3) | Blood | Positive |
| Oils from fish and marine mammals | EPA (cis-20:5n-3) | Blood | Positive |
| Oils from fish and marine mammals | Fatty acids, n3-polyunsaturated (n3-PUFA) | Blood | Positive |
| Butter | Margaric acid (17:0) | Blood | Promising |
| Butter | Methyl palmitic acid (C17H34O2) | Blood | Promising |
| Butter | Pentadecylic acid (15:0) | Blood | Promising |
| Margarine | Palmitelaidic acid (trans-16:1n-7) | Blood | Promising |

- 1. Blood/plasma fatty acids, particularly long chain poly-unsaturated fatty acids are relatively good biomarkers for the consumption of plant based oils and fats.

1. **Conclusion: least validated/promising dietary biomarkers**
   1. Polyphenols and the specific types are not specific to oils and butter and can be found in a large range of food items and are thus non-specific.

References

1. Walsham NE, Sherwood RA. Ethyl Glucuronide and Ethyl Sulfate. In: Makowski GS, ed. *Advances in Clinical Chemistry, Vol 67*. 2014:47-71. *Advances in Clinical Chemistry*.

2. Gnann H, Weinmann W, Thierauf A. Formation of Phosphatidylethanol and Its Subsequent Elimination During an Extensive Drinking Experiment Over 5 Days. *Alcoholism-Clinical and Experimental Research*. Sep 2012;36(9):1507-1511. doi:10.1111/j.1530-0277.2012.01768.x

3. Beck O, Helander A. 5-hydroxytryptophol as a marker for recent alcohol intake. *Addiction*. Dec 2003;98 Suppl 2:63-72. doi:10.1046/j.1359-6357.2003.00583.x

4. Quifer-Rada P, Chiva-Blanch G, Jauregui O, Estruch R, Lamuela-Raventos RM. A discovery-driven approach to elucidate urinary metabolome changes after a regular and moderate consumption of beer and nonalcoholic beer in subjects at high cardiovascular risk. *Molecular Nutrition & Food Research*. Oct 2017;61(10)1600980. doi:10.1002/mnfr.201600980

5. Quifer-Rada P, Martinez-Huelamo M, Chiva-Blanch G, Jauregui O, Estruch R, Lamuela-Raventos RM. Urinary Isoxanthohumol Is a Specific and Accurate Biomarker of Beer Consumption. *Journal of Nutrition*. Apr 2014;144(4):484-488. doi:10.3945/jn.113.185199

6. Esteban-Fernandez A, Ibanez C, Simo C, Bartolome B, Victoria Moreno-Arribas M. An Ultrahigh-Performance Liquid Chromatography-Time-of-Flight Mass Spectrometry Metabolomic Approach to Studying the Impact of Moderate Red-Wine Consumption on Urinary Metabolome. *Journal of Proteome Research*. Apr 2018;17(4):1624-1635. doi:10.1021/acs.jproteome.7b00904

7. Gurdeniz G, Jensen MG, Meier S, Bech L, Lund E, Dragsted LO. Detecting Beer Intake by Unique Metabolite Patterns. *Journal of Proteome Research*. Dec 2016;15(12):4544-4556. doi:10.1021/acs.jproteome.6b00635

8. Liu SQ. Malolactic fermentation in wine - beyond deacidification. *Journal of Applied Microbiology*. 2002 2002;92(4):589-601. doi:10.1046/j.1365-2672.2002.01589.x

9. Son H-S, Kim KM, Van den Berg F, et al. H-1 nuclear magnetic resonance-based metabolomic characterization of wines by grape varieties and production areas. *Journal of Agricultural and Food Chemistry*. Sep 10 2008;56(17):8007-8016. doi:10.1021/jf801424u

10. Son H-S, Hwang G-S, Kim KM, et al. Metabolomic Studies on Geographical Grapes and Their Wines Using H-1 NMR Analysis Coupled with Multivariate Statistics. *Journal of Agricultural and Food Chemistry*. Feb 25 2009;57(4):1481-1490. doi:10.1021/jf803388w

11. Vazquez-Fresno R, Llorach R, Alcaro F, et al. 1H-NMR-based metabolomic analysis of the effect of moderate wine consumption on subjects with cardiovascular risk factors. *Electrophoresis*. Aug 2012;33(15):2345-2354. doi:10.1002/elps.201100646

12. Zamora-Ros R, Achaintre D, Rothwell JA, et al. Urinary excretions of 34 dietary polyphenols and their associations with lifestyle factors in the EPIC cohort study. *Scientific Reports*. Jun 7 2016;626905. doi:10.1038/srep26905

13. Mennen LI, Sapinho D, Ito H, et al. Urinary flavonoids and phenolic acids as biomarkers of intake for polyphenol-rich foods. *British Journal of Nutrition*. Jul 2006;96(1):191-198. doi:10.1079/bjn20061808

14. Neveu V, Perez-Jimenez J, Vos F, et al. Phenol-Explorer: an online comprehensive database on polyphenol contents in foods. *Database-the Journal of Biological Databases and Curation*. 2010 2010;bap024. doi:10.1093/database/bap024

15. Zamora-Ros R, Urpi-Sarda M, Lamuela-Raventos RM, et al. Resveratrol metabolites in urine as a biomarker of wine intake in free-living subjects: The PREDIMED Study. *Free Radical Biology and Medicine*. Jun 15 2009;46(12):1562-1566. doi:10.1016/j.freeradbiomed.2008.12.023

16. Zamora-Ros R, Urpi-Sarda M, Lamuela-Raventos RM, et al. Diagnostic performance of urinary resveratrol metabolites as a biomarker of moderate wine consumption. *Clinical Chemistry*. Jul 2006;52(7):1373-1380. doi:10.1373/clinchem.2005.065870

17. Regueiro J, Vallverdu-Queralt A, Simal-Gandara J, Estruch R, Lamuela-Raventos R. Development of a LC-ESI-MS/MS Approach for the Rapid Quantification of Main Wine Organic Acids in Human Urine. *Journal of Agricultural and Food Chemistry*. Jul 10 2013;61(27):6763-6768. doi:10.1021/jf401839g

18. Bogaki T, Mitani K, Oura Y, Ozeki K. Effects of ethyl-alpha-d-glucoside on human dermal fibroblasts. *Bioscience Biotechnology and Biochemistry*. 2017 2017;81(9):1706-1711. doi:10.1080/09168451.2017.1353400

19. Bendtsen P, Jones AW, Helander A. Urinary excretion of methanol and 5-hydroxytryptophol as biochemical markers of recent drinking in the hangover state. *Alcohol and Alcoholism*. Jul-Aug 1998;33(4):431-438.

20. Helander A, Beck O, Jones AW. Laboratory testing for recent alcohol consumption: Comparison of ethanol, methanol, and 5-hydroxytryptophol. *Clinical Chemistry*. Apr 1996;42(4):618-624.

21. Wang Y, Hodge RA, Stevens VL, Hartman TJ, McCullough ML. Identification and Reproducibility of Plasma Metabolomic Biomarkers of Habitual Food Intake in a US Diet Validation Study. *Metabolites*. Sep 26 2020;10(10)doi:10.3390/metabo10100382

22. Wishart DS, Feunang YD, Marcu A, et al. HMDB 4.0: the human metabolome database for 2018. *Nucleic Acids Research*. Jan 4 2018;46(D1):D608-D617. doi:10.1093/nar/gkx1089

23. Berry SK. Role of acidulants in food industry. *Journal of Food Science and Technology-Mysore*. Mar-Apr 2001;38(2):93-104.

24. Paprotny L, Celejewska A, Frajberg M, Wianowska D. Development and validation of GC-MS/MS method useful in diagnosing intestinal dysbiosis. *Journal of Chromatography B-Analytical Technologies in the Biomedical and Life Sciences*. Nov 1 2019;1130121822. doi:10.1016/j.jchromb.2019.121822

25. Zabela V, Sampath C, Oufir M, Moradi-Afrapoli F, Butterweck V, Hamburger M. Pharmacokinetics of dietary kaempferol and its metabolite 4-hydroxyphenylacetic acid in rats. *Fitoterapia*. Dec 2016;115:189-197. doi:10.1016/j.fitote.2016.10.008

26. Gao K, Xu AL, Krul C, et al. Of the major phenolic acids formed during human microbial fermentation of tea, citrus, and soy flavonoid supplements, only 3,4-dihydroxyphenylacetic acid has antiproliferative activity. *Journal of Nutrition*. Jan 2006;136(1):52-57.

27. Hjerpsted JB, Ritz C, Schou SS, Tholstrup T, Dragsted LO. Effect of cheese and butter intake on metabolites in urine using an untargeted metabolomics approach. *Metabolomics*. Dec 2014;10(6):1176-1185. doi:10.1007/s11306-014-0657-7

28. Edmands WMB, Ferrari P, Rothwell JA, et al. Polyphenol metabolome in human urine and its association with intake of polyphenol-rich foods across European countries. *American Journal of Clinical Nutrition*. Oct 2015;102(4):905-913. doi:10.3945/ajcn.114.101881

29. Playdon MC, Sampson JN, Cross AJ, et al. Comparing metabolite profiles of habitual diet in serum and urine. *American Journal of Clinical Nutrition*. Sep 2016;104(3):776-789. doi:10.3945/ajcn.116.135301

30. Vazquez-Fresno R, Llorach R, Urpi-Sarda M, et al. An NMR metabolomics approach reveals a combined-biomarkers model in a wine interventional trial with validation in free-living individuals of the PREDIMED study. *Metabolomics*. Aug 2015;11(4):797-806. doi:10.1007/s11306-014-0735-x

31. Dahl H, Stephanson N, Beck O, Helander A. Comparison of urinary excretion characteristics of ethanol and ethyl glucuronide. *Journal of Analytical Toxicology*. May-Jun 2002;26(4):201-204. doi:10.1093/jat/26.4.201

32. Schmitt G, Droenner P, Skopp G, Aderjan R. Ethyl glucuronide concentration in serum of human volunteers, teetotalers, and suspected drinking drivers. *Journal of Forensic Sciences*. Nov 1997;42(6):1099-1102.

33. Stephanson N, Dahl H, Helander A, Beck O. Direct quantification of ethyl glucuronide in clinical urine samples by liquid chromatography-mass spectrometry. *Therapeutic Drug Monitoring*. Oct 2002;24(5):645-651. doi:10.1097/00007691-200210000-00011

34. Hoiseth G, Bernard JP, Karinen R, et al. A pharmacokinetic study of ethyl glucuronide in blood and urine: Applications to forensic toxicology. *Forensic Science International*. Oct 25 2007;172(2-3):119-124. doi:10.1016/j.forsciint.2007.01.005

35. Guertin KA, Moore SC, Sampson JN, et al. Metabolomics in nutritional epidemiology: identifying metabolites associated with diet and quantifying their potential to uncover diet-disease relations in populations. *American Journal of Clinical Nutrition*. Jul 2014;100(1):208-217. doi:10.3945/ajcn.113.078758

36. Helander A, Bottcher M, Dahmen N, Beck O. Elimination Characteristics of the Alcohol Biomarker Phosphatidylethanol (PEth) in Blood during Alcohol Detoxification. *Alcohol and Alcoholism*. May 2019;54(3):251-257. doi:10.1093/alcalc/agz027

37. Simon TW. Providing context for phosphatidylethanol as a biomarker of alcohol consumption with a pharmacokinetic model. *Regulatory Toxicology and Pharmacology*. Apr 2018;94:163-171. doi:10.1016/j.yrtph.2018.01.029

38. Lopez-Cruzan M, Roache JD, Hill-Kapturczak N, et al. Pharmacokinetics of Phosphatidylethanol 16:0/20:4 in Human Blood After Alcohol Intake. *Alcoholism-Clinical and Experimental Research*. Nov 2018;42(11):2094-2099. doi:10.1111/acer.13865

39. Hill-Kapturczak N, Dougherty DM, Roache JD, Karns-Wright TE, Javors MA. Differences in the Synthesis and Elimination of Phosphatidylethanol 16:0/18:1 and 16:0/18:2 After Acute Doses of Alcohol. *Alcoholism-Clinical and Experimental Research*. May 2018;42(5):851-860. doi:10.1111/acer.13620

40. van Breemen RB, Yuan Y, Banuvar S, et al. Pharmacokinetics of prenylated hop phenols in women following oral administration of a standardized extract of hops. *Molecular Nutrition & Food Research*. Oct 2014;58(10):1962-1969. doi:10.1002/mnfr.201400245

41. Rodda LN, Gerostamoulos D, Drummer OH. Pharmacokinetics of Iso-alpha-Acids in Volunteers Following the Consumption of Beer. *Journal of Analytical Toxicology*. Jul-Aug 2014;38(6):354-359. doi:10.1093/jat/bku038

42. Hoiseth G, Morini L, Polettini A, Christophersen A, Morland J. Blood kinetics of ethyl glucuronide and ethyl sulphate in heavy drinkers during alcohol detoxification. *Forensic Science International*. Jul 1 2009;188(1-3):52-56. doi:10.1016/j.forsciint.2009.03.017

43. Wurst FM, Dresen S, Allen JP, Wiesbeck G, Graf M, Weinmann W. Ethyl sulphate: a direct ethanol metabolite reflecting recent alcohol consumption. *Addiction*. Feb 2006;101(2):204-211. doi:10.1111/j.1360-0443.2005.01245.x

44. Karacaoglu E, Odabasi AB, Akcan R, et al. Time dependent change of ethanol consumption biomarkers, ethyl glucuronide and ethyl sulphate, after single dose ethanol intake. *Turkish Journal of Biochemistry-Turk Biyokimya Dergisi*. Jun 2019;44(3):379-387. doi:10.1515/tjb-2018-0173

45. Agueusop I, Musholt PB, Klaus B, Hightower K, Kannt A. Short-term variability of the human serum metabolome depending on nutritional and metabolic health status. *Scientific Reports*. 2020/10/01 2020;10(1):16310. doi:10.1038/s41598-020-72914-7

46. Shahrzad S, Aoyagi K, Winter A, Koyama A, Bitsch I. Pharmacokinetics of gallic acid and its relative bioavailability from tea in healthy humans. *Journal of Nutrition*. Apr 2001;131(4):1207-1210.

47. van der Pijl PC, Foltz M, Glube ND, Peters S, Duchateau GSMJE. Pharmacokinetics of black tea-derived phenolic acids in plasma. *Journal of Functional Foods*. Aug 2015;17:667-675. doi:10.1016/j.jff.2015.06.020

48. Walle T, Hsieh F, DeLegge MH, Oatis JE, Walle UK. High absorption but very low bioavailability of oral resveratrol in humans. *Drug Metabolism and Disposition*. Dec 2004;32(12):1377-1382. doi:10.1124/dmd.104.000885

49. Miro-Casas E, Covas MI, Farre M, et al. Hydroxytyrosol disposition in humans. *Clinical Chemistry*. Jun 2003;49(6):945-952. doi:10.1373/49.6.945

50. Jones AW. Alcohol, its absorption, distribution, metabolism, and excretion in the body and pharmacokinetic calculations. *WIREs Forensic Science*. 2019;1(5):e1340. doi:<https://doi.org/10.1002/wfs2.1340>

51. Landberg R, Hanhineva K, Tuohy K, et al. Biomarkers of cereal food intake. *Genes Nutr*. 2019;14:28. doi:10.1186/s12263-019-0651-9

52. Ross AB, Kamal-Eldin A, Åman P. Dietary Alkylresorcinols: Absorption, Bioactivities, and Possible Use as Biomarkers of Whole-grain Wheat–and Rye–rich Foods. *Nutrition Reviews*. 2004;62(3):81-95. doi:10.1111/j.1753-4887.2004.tb00029.x

53. Ross AB, Svelander C, Karlsson G, Savolainen OI. Identification and quantification of even and odd chained 5-n alkylresorcinols, branched chain-alkylresorcinols and methylalkylresorcinols in Quinoa (Chenopodium quinoa). *Food Chem*. Apr 1 2017;220:344-351. doi:10.1016/j.foodchem.2016.10.020

54. Wierzbicka R, Zamaratskaia G, Kamal-Eldin A, Landberg R. Novel urinary alkylresorcinol metabolites as biomarkers of whole grain intake in free-living Swedish adults. *Mol Nutr Food Res*. Jul 2017;61(7)doi:10.1002/mnfr.201700015

55. Wang P, Yang J, Yerke A, Sang S. Avenacosides: Metabolism, and potential use as exposure biomarkers of oat intake. *Mol Nutr Food Res*. Jul 2017;61(7)doi:10.1002/mnfr.201700196

56. Wang P, Zhang S, Yerke A, et al. Avenanthramide Metabotype from Whole-Grain Oat Intake is Influenced by Faecalibacterium prausnitzii in Healthy Adults. *J Nutr*. Jun 1 2021;151(6):1426-1435. doi:10.1093/jn/nxab006

57. Sang S. Biomarkers of Whole Grain Intake. *J Agric Food Chem*. Oct 10 2018;66(40):10347-10352. doi:10.1021/acs.jafc.8b04110

58. Nordin E, Steffensen SK, Laursen BB, et al. An inverse association between plasma benzoxazinoid metabolites and PSA after rye intake in men with prostate cancer revealed with a new method. *Sci Rep*. Mar 28 2022;12(1):5260. doi:10.1038/s41598-022-08856-z

59. Wu H, Mhd Omar NA, Håkansson N, Wolk A, Michaëlsson K, Landberg R. Evaluation of alkylresorcinols in adipose tissue biopsies as a long-term biomarker of whole-grain wheat and rye intake in free-living Swedish men and women. *Public Health Nutr*. Jul 2018;21(10):1933-1942. doi:10.1017/s1368980018000484

60. Sang S. Biomarkers of Whole Grain Intake. *Journal of Agricultural and Food Chemistry*. 2018/10/10 2018;66(40):10347-10352. doi:10.1021/acs.jafc.8b04110

61. Wierzbicka R, Wu H, Franek M, Kamal-Eldin A, Landberg R. Determination of alkylresorcinols and their metabolites in biological samples by gas chromatography-mass spectrometry. *J Chromatogr B Analyt Technol Biomed Life Sci*. Sep 1 2015;1000:120-9. doi:10.1016/j.jchromb.2015.07.009

62. Koskela A, Linko-Parvinen AM, Hiisivuori P, et al. Quantification of alkylresorcinol metabolites in urine by HPLC with coulometric electrode array detection. *Clin Chem*. Jul 2007;53(7):1380-3. doi:10.1373/clinchem.2006.084764

63. Linko AM, Parikka K, Wähälä K, Adlercreutz H. Gas chromatographic-mass spectrometric method for the determination of alkylresorcinols in human plasma. *Anal Biochem*. Sep 15 2002;308(2):307-13. doi:10.1016/s0003-2697(02)00226-9

64. Wierzbicka R, Eyer L, Landberg R, Kamal-Eldin A, Franek M. Development of antibodies for determination of alkylresorcinol metabolites in human urine and elucidation of ELISA cross-reactivity. *J Immunol Methods*. Nov 2014;413:12-24. doi:10.1016/j.jim.2014.07.007

65. Jensen BM, Adhikari KB, Schnoor HJ, Juel-Berg N, Fomsgaard IS, Poulsen LK. Quantitative analysis of absorption, metabolism, and excretion of benzoxazinoids in humans after the consumption of high- and low-benzoxazinoid diets with similar contents of cereal dietary fibres: a crossover study. *Eur J Nutr*. Feb 2017;56(1):387-397. doi:10.1007/s00394-015-1088-6

66. Sang S, Chu Y. Whole grain oats, more than just a fiber: Role of unique phytochemicals. *Mol Nutr Food Res*. Jul 2017;61(7)doi:10.1002/mnfr.201600715

67. Beckmann M, Lloyd AJ, Haldar S, Seal C, Brandt K, Draper J. Hydroxylated phenylacetamides derived from bioactive benzoxazinoids are bioavailable in humans after habitual consumption of whole grain sourdough rye bread. *Mol Nutr Food Res*. Oct 2013;57(10):1859-73. doi:10.1002/mnfr.201200777

68. Hanhineva K, Brunius C, Andersson A, et al. Discovery of urinary biomarkers of whole grain rye intake in free-living subjects using nontargeted LC-MS metabolite profiling. *Molecular Nutrition & Food Research*. 2015;59(11):2315-2325. doi:<https://doi.org/10.1002/mnfr.201500423>

69. Jawhara M, Sørensen SB, Heitmann BL, Andersen V. Biomarkers of Whole-Grain and Cereal-Fiber Intake in Human Studies: A Systematic Review of the Available Evidence and Perspectives. *Nutrients*. Dec 6 2019;11(12)doi:10.3390/nu11122994

70. Landberg R, Linko AM, Kamal-Eldin A, Vessby B, Adlercreutz H, Aman P. Human plasma kinetics and relative bioavailability of alkylresorcinols after intake of rye bran. *J Nutr*. Nov 2006;136(11):2760-5. doi:10.1093/jn/136.11.2760

71. Söderholm PP, Lundin JE, Koskela AH, Tikkanen MJ, Adlercreutz HC. Pharmacokinetics of alkylresorcinol metabolites in human urine. *Br J Nutr*. Oct 2011;106(7):1040-4. doi:10.1017/s0007114511001383

72. Söderholm PP, Koskela AH, Lundin JE, Tikkanen MJ, Adlercreutz HC. Plasma pharmacokinetics of alkylresorcinol metabolites: new candidate biomarkers for whole-grain rye and wheat intake. *Am J Clin Nutr*. Nov 2009;90(5):1167-71. doi:10.3945/ajcn.2009.28290

73. Zhu Y, Shurlknight KL, Chen X, Sang S. Identification and pharmacokinetics of novel alkylresorcinol metabolites in human urine, new candidate biomarkers for whole-grain wheat and rye intake. *J Nutr*. Feb 2014;144(2):114-22. doi:10.3945/jn.113.184663

74. Landberg R, Aman P, Friberg LE, Vessby B, Adlercreutz H, Kamal-Eldin A. Dose response of whole-grain biomarkers: alkylresorcinols in human plasma and their metabolites in urine in relation to intake. *Am J Clin Nutr*. Jan 2009;89(1):290-6. doi:10.3945/ajcn.2008.26709

75. Wolk A, Vessby B, Ljung H, Barrefors P. Evaluation of a biological marker of dairy fat intake. *Am J Clin Nutr*. Aug 1998;68(2):291-5. doi:10.1093/ajcn/68.2.291

76. Mozaffarian D, de Oliveira Otto MC, Lemaitre RN, et al. trans-Palmitoleic acid, other dairy fat biomarkers, and incident diabetes: the Multi-Ethnic Study of Atherosclerosis (MESA). *Am J Clin Nutr*. Apr 2013;97(4):854-61. doi:10.3945/ajcn.112.045468

77. Münger LH, Trimigno A, Picone G, et al. Identification of Urinary Food Intake Biomarkers for Milk, Cheese, and Soy-Based Drink by Untargeted GC-MS and NMR in Healthy Humans. *J Proteome Res*. Sep 1 2017;16(9):3321-3335. doi:10.1021/acs.jproteome.7b00319

78. Rouge P, Cornu A, Biesse-Martin AS, Lyan B, Rochut N, Graulet B. Identification of quinoline, carboline and glycinamide compounds in cow milk using HRMS and NMR. *Food Chem*. Dec 1 2013;141(3):1888-94. doi:10.1016/j.foodchem.2013.04.072

79. Rodríguez-Gómez R, García-Córcoles MT, Çipa M, Barrón D, Navalón A, Zafra-Gómez A. Determination of quinolone residues in raw cow milk. Application of polar stir-bars and ultra-high performance liquid chromatography-tandem mass spectrometry. *Food Addit Contam Part A Chem Anal Control Expo Risk Assess*. Jun 2018;35(6):1127-1138. doi:10.1080/19440049.2018.1430382

80. Vionnet N, Münger LH, Freiburghaus C, et al. Assessment of lactase activity in humans by measurement of galactitol and galactonate in serum and urine after milk intake. *Am J Clin Nutr*. Feb 1 2019;109(2):470-477. doi:10.1093/ajcn/nqy296

81. Sofie Biong A, Berstad P, Pedersen JI. Biomarkers for intake of dairy fat and dairy products. *European Journal of Lipid Science and Technology*. 2006;108(10):827-834. doi:<https://doi.org/10.1002/ejlt.200600044>

82. Baylin A, Kabagambe EK, Siles X, Campos H. Adipose tissue biomarkers of fatty acid intake. *The American Journal of Clinical Nutrition*. 2002;76(4):750-757. doi:10.1093/ajcn/76.4.750

83. Brevik A, Veierød MB, Drevon CA, Andersen LF. Evaluation of the odd fatty acids 15:0 and 17:0 in serum and adipose tissue as markers of intake of milk and dairy fat. *Eur J Clin Nutr*. Dec 2005;59(12):1417-22. doi:10.1038/sj.ejcn.1602256

84. Guertin KA, Moore SC, Sampson JN, et al. Metabolomics in nutritional epidemiology: identifying metabolites associated with diet and quantifying their potential to uncover diet-disease relations in populations. *Am J Clin Nutr*. Jul 2014;100(1):208-17. doi:10.3945/ajcn.113.078758

85. Sun Q, Ma J, Campos H, Hu FB. Plasma and erythrocyte biomarkers of dairy fat intake and risk of ischemic heart disease. *The American Journal of Clinical Nutrition*. 2007;86(4):929-937. doi:10.1093/ajcn/86.4.929

86. Yakoob MY, Shi P, Hu FB, et al. Circulating biomarkers of dairy fat and risk of incident stroke in U.S. men and women in 2 large prospective cohorts. *Am J Clin Nutr*. Dec 2014;100(6):1437-47. doi:10.3945/ajcn.114.083097

87. Yakoob MY, Shi P, Willett WC, et al. Circulating Biomarkers of Dairy Fat and Risk of Incident Diabetes Mellitus Among Men and Women in the United States in Two Large Prospective Cohorts. *Circulation*. Apr 26 2016;133(17):1645-54. doi:10.1161/circulationaha.115.018410

88. Micha R, King IB, Lemaitre RN, et al. Food sources of individual plasma phospholipid trans fatty acid isomers: the Cardiovascular Health Study. *Am J Clin Nutr*. Apr 2010;91(4):883-93. doi:10.3945/ajcn.2009.28877

89. Playdon MC, Ziegler RG, Sampson JN, et al. Nutritional metabolomics and breast cancer risk in a prospective study. *Am J Clin Nutr*. Aug 2017;106(2):637-649. doi:10.3945/ajcn.116.150912

90. Wang Y, Gapstur SM, Carter BD, et al. Untargeted Metabolomics Identifies Novel Potential Biomarkers of Habitual Food Intake in a Cross-Sectional Study of Postmenopausal Women. *J Nutr*. Jun 1 2018;148(6):932-943. doi:10.1093/jn/nxy027

91. Smedman AE, Gustafsson IB, Berglund LG, Vessby BO. Pentadecanoic acid in serum as a marker for intake of milk fat: relations between intake of milk fat and metabolic risk factors. *Am J Clin Nutr*. Jan 1999;69(1):22-9. doi:10.1093/ajcn/69.1.22

92. Playdon MC, Sampson JN, Cross AJ, et al. Comparing metabolite profiles of habitual diet in serum and urine. *Am J Clin Nutr*. Sep 2016;104(3):776-89. doi:10.3945/ajcn.116.135301

93. Allen NE, Grace PB, Ginn A, et al. Phytanic acid: measurement of plasma concentrations by gas-liquid chromatography-mass spectrometry analysis and associations with diet and other plasma fatty acids. *Br J Nutr*. Mar 2008;99(3):653-9. doi:10.1017/s000711450782407x

94. Kotsopoulos J, Tworoger SS, Campos H, et al. Reproducibility of plasma and urine biomarkers among premenopausal and postmenopausal women from the Nurses' Health Studies. *Cancer Epidemiol Biomarkers Prev*. Apr 2010;19(4):938-46. doi:10.1158/1055-9965.Epi-09-1318

95. Park YJ, Volpe SL, Decker EA. Quantitation of carnosine in humans plasma after dietary consumption of beef. *Journal of Agricultural and Food Chemistry*. Jun 15 2005;53(12):4736-4739. doi:10.1021/jf047934h

96. Block WD, Hubbard RW, Steele BF. Excretion of histidine and histidine derivatives by human subjects ingesting protein from different sources. *The Journal of nutrition*. 1965-Apr 1965;85:419-25.

97. Sjolin J, Hjort G, Friman G, Hambraeus L. Urinary excretion of 1-methylhistidine: A qualitative indicator of exogenous 3-methylhistidine and intake of meats from various sources. *Metabolism-Clinical and Experimental*. Dec 1987;36(12):1175-1184. doi:10.1016/0026-0495(87)90245-9

98. Gil-Agusti M, Esteve-Romero J, Carda-Broch S. Anserine and carnosine determination in meat samples by pure micellar liquid chromatography. *Journal of Chromatography A*. May 2 2008;1189(1-2):444-450. doi:10.1016/j.chroma.2007.11.075

99. Abe H, Okuma E, Sekine H, Maeda A, Yoshiue S. Human urinary excretion of L-histidine-related compounds after ingestion of several meats and fish muscle. Journal Article. *Int J Biochem*. Sep 1993;25(9):1245-9.

100. Abe H. Distribution of free l-histidine and related dipeptides in the muscle of fresh-water fishes. *Comparative Biochemistry and Physiology Part B: Comparative Biochemistry*. // 1983;76(1):35-39. doi:<http://dx.doi.org/10.1016/0305-0491(83)90167-0>

101. Allen NE, Grace PB, Ginn A, et al. Phytanic acid: measurement of plasma concentrations by gas-liquid chromatography-mass spectrometry analysis and associations with diet and other plasma fatty acids. 10.1017/S000711450782407X. *Br J Nutr*. 2008;99(3):653-659.

102. Wang Z, Klipfell E, Bennett BJ, et al. Gut flora metabolism of phosphatidylcholine promotes cardiovascular disease. *Nature*. 2011 2011;472(7341):57-63.

103. Koeth RA, Wang Z, Levison BS, et al. Intestinal microbiota metabolism of l-carnitine, a nutrient in red meat, promotes atherosclerosis. Article. *Nature Medicine*. 2013;19:576-85. doi:10.1038/nm.3145

104. Cheung W, Keski-Rahkonen P, Assi N, et al. A metabolomic study of biomarkers of meat and fish intake. *The American Journal of Clinical Nutrition*. January 25, 2017 2017;105(3):600-608. doi:10.3945/ajcn.116.146639

105. Gibson R, Lau C-HE, Loo RL, et al. The association of fish consumption and its urinary metabolites with cardiovascular risk factors: the International Study of Macro-/Micronutrients and Blood Pressure (INTERMAP). *The American Journal of Clinical Nutrition*. 2019;111(2):280-290. doi:10.1093/ajcn/nqz293

106. Ohshima H, Bartsch H. [5] Quantitative estimation of endogenous N-nitrosation in humans by monitoring N-nitrosoproline in urine. *Methods in Enzymology*. Academic Press; 1999:40-49.

107. Kidd LCR, Stillwell WG, Yu MC, et al. Urinary excretion of 2-amino-1-methyl-6-phenylimidazo 4,5-b pyridine (PhIP) in white, African-American, and Asian-American men in Los Angeles County. *Cancer Epidemiology Biomarkers & Prevention*. May 1999;8(5):439-445.

108. Stich HF, Hornby AP, Dunn BP. THE EFFECT OF DIETARY FACTORS ON NITROSOPROLINE LEVELS IN HUMAN-URINE. *International Journal of Cancer*. 1984 1984;33(5):625-628. doi:10.1002/ijc.2910330512

109. Herrmann SS, Duedahl-Olesen L, Granby K. Occurrence of volatile and non-volatile N-nitrosamines in processed meat products and the role of heat treatment. *Food Control*. 2015/02/01/ 2015;48:163-169. doi:<https://doi.org/10.1016/j.foodcont.2014.05.030>

110. Stuff JE, Goh ET, Barrera SL, Bondy ML, Forman MR. Construction of an N-nitroso database for assessing dietary intake. *Journal of Food Composition and Analysis: 22 (Suppl) S42-S47*. 2009;22(Suppl.):42-S47.

111. Wedekind R, Keski-Rahkonen P, Robinot N, et al. Syringol metabolites as new biomarkers for smoked meat intake. *American Journal of Clinical Nutrition*. 2019;110(6):1424-1433.

112. Wedekind R, Keski-Rahkonen P, Robinot N, et al. Pepper Alkaloids and Processed Meat Intake: Results from a Randomized Trial and the European Prospective Investigation into Cancer and Nutrition (EPIC) Cohort. *Molecular Nutrition & Food Research*. 2021/02/16 2021;n/a(n/a):2001141. doi:<https://doi.org/10.1002/mnfr.202001141>

113. Mitry P, Wawro N, Rohrmann S, Giesbertz P, Daniel H, Linseisen J. Plasma concentrations of anserine, carnosine and pi-methylhistidine as biomarkers of habitual meat consumption. *European Journal of Clinical Nutrition*. 2019/05/01 2019;73(5):692-702. doi:10.1038/s41430-018-0248-1

114. Myint T, Fraser GE, Lindsted KD, Knutsen SF, Hubbard RW, Bennett HW. Urinary 1-methylhistidine is a marker of meat consumption in black and in white California seventh-day Adventists. 10.1093/aje/152.8.752. *American Journal of Epidemiology*. 2000;152(8):752-755.

115. Pallister T, Jennings A, Mohney RP, et al. Characterizing Blood Metabolomics Profiles Associated with Self-Reported Food Intakes in Female Twins. *PLoS One*. 2016;11(6):e0158568. doi:10.1371/journal.pone.0158568

116. Shibutami E, Ishii R, Harada S, et al. Charged metabolite biomarkers of food intake assessed via plasma metabolomics in a population-based observational study in Japan. *PLoS One*. 2021;16(2):e0246456. doi:10.1371/journal.pone.0246456

117. Yeum K-J, Orioli M, Regazzoni L, et al. Profiling histidine dipeptides in plasma and urine after ingesting beef, chicken or chicken broth in humans. 10.1007/s00726-009-0291-2. *Amino Acids*. 2010;38:847-58.

118. Fraser GE, Jaceldo-Siegl K, Henning SM, et al. Biomarkers of Dietary Intake Are Correlated with Corresponding Measures from Repeated Dietary Recalls and Food-Frequency Questionnaires in the Adventist Health Study-2. *J Nutr*. Feb 3 2016;doi:10.3945/jn.115.225508

119. Ji H, Yu MC, Stillwell WG, et al. URINARY-EXCRETION OF 2-AMINO-3,8-DIMETHYLIMIDAZO- 4,5-F QUINOXALINE IN WHITE, BLACK, AND ASIAN MEN IN LOS-ANGELES-COUNTY. *Cancer Epidemiology Biomarkers & Prevention*. Jul-Aug 1994;3(5):407-411.

120. Kobayashi M, Hanaoka T, Hashimoto H, Tsugane S. 2-amino-1-methyl-6-phenylimidazo 4,5-b pyridine (PhIP) level in human hair as biomarkers for dietary grilled/stir-fried meat and fish intake. *Mutation Research-Genetic Toxicology and Environmental Mutagenesis*. Dec 30 2005;588(2):136-142. doi:10.1016/j.mrgentox.2005.09.008

121. Bachmann K, Galeazzi R, Millet A, Burger AG. Plasma levels of 3-methylhistidine after ingestion of the pure amino acid or of muscular proteins measured by radioimmunoassay. *Metabolism*. 1984/02/01/ 1984;33(2):107-110. doi:<https://doi.org/10.1016/0026-0495(84)90120-3>

122. Yin X, Gibbons H, Rundle M, et al. Estimation of Chicken Intake by Adults Using Metabolomics-Derived Markers. *The Journal of Nutrition*. October 1, 2017 2017;147(10):1850-1857. doi:10.3945/jn.117.252197

123. Kochlik B, Gerbracht C, Grune T, Weber D. The Influence of Dietary Habits and Meat Consumption on Plasma 3-Methylhistidine-A Potential Marker for Muscle Protein Turnover. *Molecular Nutrition & Food Research*. May 2018;62(9)1701062. doi:10.1002/mnfr.201701062

124. Xiao Q, Moore SC, Boca SM, et al. Sources of Variability in Metabolite Measurements from Urinary Samples. *Plos One*. May 1 2014;9(5)e95749. doi:10.1371/journal.pone.0095749

125. Maitre L, Lau C-HE, Vizcaino E, et al. Assessment of metabolic phenotypic variability in children’s urine using 1H NMR spectroscopy. Article. *Scientific Reports*. 04/19/online 2017;7:46082. doi:10.1038/srep46082

<https://www.nature.com/articles/srep46082#supplementary-information>

126. Zheng Y, Yu B, Alexander D, Couper DJ, Boerwinkle E. Medium-Term Variability of the Human Serum Metabolome in the Atherosclerosis Risk in Communities (ARIC) Study. *OMICS : a Journal of Integrative Biology*. 2014;18(6):364-373. doi:10.1089/omi.2014.0019

127. Sampson JN, Boca SM, Shu XO, et al. Metabolomics in epidemiology: sources of variability in metabolite measurements and implications. *Cancer epidemiology, biomarkers & prevention*. 2013-Apr 2013;22(4):631-40. doi:10.1158/1055-9965.epi-12-1109

128. Townsend MK, Clish CB, Kraft P, et al. Reproducibility of metabolomic profiles among men and women in 2 large cohort studies. Journal Article

Research Support, N.I.H., Extramural

Research Support, Non-U.S. Gov't. *Clin Chem*. Nov 2013;59(11):1657-67.

129. Vetter W, Wendlinger C. Furan fatty acids – valuable minor fatty acids in food. *Lipid Technology*. 2013;25(1):7-10. doi:<https://doi.org/10.1002/lite.201300247>

130. Hannemann K, Puchta V, Simon E, Ziegler H, Ziegler G, Spiteller G. The common occurrence of furan fatty acids in plants. *Lipids*. 1989/04/01 1989;24(4):296-298. doi:10.1007/BF02535166

131. Hanhineva K, Lankinen MA, Pedret A, et al. Nontargeted metabolite profiling discriminates diet-specific biomarkers for consumption of whole grains, fatty fish, and bilberries in a randomized controlled trial. *J Nutr*. Jan 2015;145(1):7-17. doi:10.3945/jn.114.196840

132. Zheng Y, Yu B, Alexander D, Steffen LM, Boerwinkle E. Human metabolome associates with dietary intake habits among African Americans in the atherosclerosis risk in communities study. *Am J Epidemiol*. Jun 15 2014;179(12):1424-33. doi:10.1093/aje/kwu073

133. Byelashov OA, Sinclair AJ, Kaur G. Dietary sources, current intakes, and nutritional role of omega-3 docosapentaenoic acid. *Lipid Technol*. Apr 2015;27(4):79-82. doi:10.1002/lite.201500013

134. Andersen M-BS, Reinbach HC, Rinnan Å, Barri T, Mithril C, Dragsted LO. Discovery of exposure markers in urine for Brassica-containing meals served with different protein sources by UPLC-qTOF-MS untargeted metabolomics. *Metabolomics*. 2013/10/01 2013;9(5):984-997. doi:10.1007/s11306-013-0522-0

135. Lloyd AJ, Fave G, Beckmann M, et al. Use of mass spectrometry fingerprinting to identify urinary metabolites after consumption of specific foods. *Am J Clin Nutr*. Oct 2011;94(4):981-91. doi:10.3945/ajcn.111.017921

136. Cheung W, Keski-Rahkonen P, Assi N, et al. A metabolomic study of biomarkers of meat and fish intake. *Am J Clin Nutr*. Mar 2017;105(3):600-608. doi:10.3945/ajcn.116.146639

137. Li-Gao R, Hughes DA, le Cessie S, et al. Assessment of reproducibility and biological variability of fasting and postprandial plasma metabolite concentrations using 1H NMR spectroscopy. *PLoS One*. 2019;14(6):e0218549. doi:10.1371/journal.pone.0218549

138. Wang Y, Gapstur SM, Carter BD, et al. Untargeted Metabolomics Identifies Novel Potential Biomarkers of Habitual Food Intake in a Cross-Sectional Study of Postmenopausal Women. *The Journal of Nutrition*. 2018;148(6):932-943. doi:10.1093/jn/nxy027

139. Edmands WM, Beckonert OP, Stella C, et al. Identification of human urinary biomarkers of cruciferous vegetable consumption by metabonomic profiling. *J Proteome Res*. Oct 7 2011;10(10):4513-21. doi:10.1021/pr200326k

140. PubChem Compound Summary for CID 122164824, N-acetylalliin. National Center for Biotechnology Information. 2022. <https://pubchem.ncbi.nlm.nih.gov/compound/N-acetylalliin>.

141. Al-Delaimy WK, Ferrari P, Slimani N, et al. Plasma carotenoids as biomarkers of intake of fruits and vegetables: individual-level correlations in the European Prospective Investigation into Cancer and Nutrition (EPIC). *Eur J Clin Nutr*. Dec 2005;59(12):1387-96. doi:10.1038/sj.ejcn.1602252

142. Hammond BR, Jr., Renzi LM. Carotenoids. *Adv Nutr*. Jul 1 2013;4(4):474-6. doi:10.3945/an.113.004028

143. Okuda M, Sasaki S, Bando N, et al. Carotenoid, tocopherol, and fatty acid biomarkers and dietary intake estimated by using a brief self-administered diet history questionnaire for older Japanese children and adolescents. *J Nutr Sci Vitaminol (Tokyo)*. Jun 2009;55(3):231-41. doi:10.3177/jnsv.55.231

144. Zheng JS, Sharp SJ, Imamura F, et al. Association of plasma biomarkers of fruit and vegetable intake with incident type 2 diabetes: EPIC-InterAct case-cohort study in eight European countries. *Bmj*. Jul 8 2020;370:m2194. doi:10.1136/bmj.m2194

145. Ey J, Schömig E, Taubert D. Dietary sources and antioxidant effects of ergothioneine. *J Agric Food Chem*. Aug 8 2007;55(16):6466-74. doi:10.1021/jf071328f

146. Metabocard for Alliin (HMDB0301759). The Metabolomics Innovation Centre. <https://hmdb.ca/metabolites/HMDB0301759#references>

147. Metabocard for S-Allylcysteine (HMDB0034323). The Metabolomics Innovation Centre.

148. Campbell DR, Gross MD, Martini MC, Grandits GA, Slavin JL, Potter JD. Plasma carotenoids as biomarkers of vegetable and fruit intake. *Cancer Epidemiol Biomarkers Prev*. Sep 1994;3(6):493-500.

149. Brantsaeter AL, Haugen M, Rasmussen SE, Alexander J, Samuelsen SO, Meltzer HM. Urine flavonoids and plasma carotenoids in the validation of fruit, vegetable and tea intake during pregnancy in the Norwegian Mother and Child Cohort Study (MoBa). *Public Health Nutr*. Aug 2007;10(8):838-47. doi:10.1017/s1368980007339037

150. Bogers RP, Van Assema P, Kester AD, Westerterp KR, Dagnelie PC. Reproducibility, validity, and responsiveness to change of a short questionnaire for measuring fruit and vegetable intake. *Am J Epidemiol*. May 1 2004;159(9):900-9. doi:10.1093/aje/kwh123

151. Marshall JR, Lanza E, Bloch A, et al. Indexes of food and nutrient intakes as predictors of serum concentrations of nutrients: the problem of inadequate discriminant validity. The Polyp Prevention Trial Study Group. *Am J Clin Nutr*. Apr 1997;65(4 Suppl):1269s-1274s. doi:10.1093/ajcn/65.4.1269S

152. Jansen MC, Van Kappel AL, Ocké MC, et al. Plasma carotenoid levels in Dutch men and women, and the relation with vegetable and fruit consumption. *Eur J Clin Nutr*. Oct 2004;58(10):1386-95. doi:10.1038/sj.ejcn.1601981

153. van Kappel AL, Steghens JP, Zeleniuch-Jacquotte A, Chajès V, Toniolo P, Riboli E. Serum carotenoids as biomarkers of fruit and vegetable consumption in the New York Women's Health Study. *Public Health Nutr*. Jun 2001;4(3):829-35. doi:10.1079/phn2000115

154. Irwig MS, El-Sohemy A, Baylin A, Rifai N, Campos H. Frequent intake of tropical fruits that are rich in beta-cryptoxanthin is associated with higher plasma beta-cryptoxanthin concentrations in Costa Rican adolescents. *J Nutr*. Oct 2002;132(10):3161-7. doi:10.1093/jn/131.10.3161

155. Toft U, Kristoffersen L, Ladelund S, et al. Relative validity of a food frequency questionnaire used in the Inter99 study. *Eur J Clin Nutr*. Aug 2008;62(8):1038-46. doi:10.1038/sj.ejcn.1602815

156. Andersen LF, Veierød MB, Johansson L, Sakhi A, Solvoll K, Drevon CA. Evaluation of three dietary assessment methods and serum biomarkers as measures of fruit and vegetable intake, using the method of triads. *Br J Nutr*. Apr 2005;93(4):519-27. doi:10.1079/bjn20041381

157. Krogholm KS, Bysted A, Brantsæter AL, et al. Evaluation of flavonoids and enterolactone in overnight urine as intake biomarkers of fruits, vegetables and beverages in the Inter99 cohort study using the method of triads. *Br J Nutr*. Nov 28 2012;108(10):1904-12. doi:10.1017/s0007114512000104

158. Mennen LI, Sapinho D, Ito H, et al. Urinary flavonoids and phenolic acids as biomarkers of intake for polyphenol-rich foods. *Br J Nutr*. Jul 2006;96(1):191-8. doi:10.1079/bjn20061808

159. Mohammadifard N, Omidvar N, Houshiarrad A, Neyestani T, Naderi GA, Soleymani B. Validity and reproducibility of a food frequency questionnaire for assessment of fruit and vegetable intake in Iranian adults(*). *J Res Med Sci*. Oct 2011;16(10):1286-97.

160. Playdon MC, Moore SC, Derkach A, et al. Identifying biomarkers of dietary patterns by using metabolomics. *Am J Clin Nutr*. Feb 2017;105(2):450-465. doi:10.3945/ajcn.116.144501

161. Zamora-Ros R, Achaintre D, Rothwell JA, et al. Urinary excretions of 34 dietary polyphenols and their associations with lifestyle factors in the EPIC cohort study. *Sci Rep*. Jun 7 2016;6:26905. doi:10.1038/srep26905

162. Dauchet L, Péneau S, Bertrais S, et al. Relationships between different types of fruit and vegetable consumption and serum concentrations of antioxidant vitamins. *Br J Nutr*. Sep 2008;100(3):633-41. doi:10.1017/s000711450892170x

163. Krogholm KS, Bredsdorff L, Alinia S, Christensen T, Rasmussen SE, Dragsted LO. Free fruit at workplace intervention increases total fruit intake: a validation study using 24 h dietary recall and urinary flavonoid excretion. *Eur J Clin Nutr*. Oct 2010;64(10):1222-8. doi:10.1038/ejcn.2010.130

164. Wu X, Cai H, Gao YT, et al. Correlations of urinary phytoestrogen excretion with lifestyle factors and dietary intakes among middle-aged and elderly Chinese women. *Int J Mol Epidemiol Genet*. 2012;3(1):18-29.

165. Busby MG, Jeffcoat AR, Bloedon LT, et al. Clinical characteristics and pharmacokinetics of purified soy isoflavones: single-dose administration to healthy men. *The American Journal of Clinical Nutrition*. 2002;75(1):126-136. doi:10.1093/ajcn/75.1.126

166. Fischer L, Mahoney C, Jeffcoat AR, et al. Clinical characteristics and pharmacokinetics of purified soy isoflavones: multiple-dose administration to men with prostate neoplasia. *Nutrition and cancer*. 2004;48(2):160-70. doi:10.1207/s15327914nc4802_5

167. Hosoda K, Furuta T, Yokokawa A, Ogura K, Hiratsuka A, Ishii K. Plasma profiling of intact isoflavone metabolites by high-performance liquid chromatography and mass spectrometric identification of flavone glycosides daidzin and genistin in human plasma after administration of kinako. *Drug metabolism and disposition: the biological fate of chemicals*. Aug 2008;36(8):1485-95. doi:10.1124/dmd.108.021006

168. Setchell KD, Clerici C. Equol: history, chemistry, and formation. *J Nutr*. Jul 2010;140(7):1355s-62s. doi:10.3945/jn.109.119776

169. Hazim S, Curtis PJ, Schär MY, et al. Acute benefits of the microbial-derived isoflavone metabolite equol on arterial stiffness in men prospectively recruited according to equol producer phenotype: a double-blind randomized controlled trial. *Am J Clin Nutr*. Mar 2016;103(3):694-702. doi:10.3945/ajcn.115.125690

170. Perera T, Young MR, Zhang Z, et al. Identification and monitoring of metabolite markers of dry bean consumption in parallel human and mouse studies. *Mol Nutr Food Res*. Apr 2015;59(4):795-806. doi:10.1002/mnfr.201400847

171. Kim MJ, Yang HJ, Kim JH, et al. Obesity-related metabolomic analysis of human subjects in black soybean peptide intervention study by ultraperformance liquid chromatography and quadrupole-time-of-flight mass spectrometry. *Journal of obesity*. 2013;2013:874981. doi:10.1155/2013/874981

172. Brevik A, Rasmussen SE, Drevon CA, Andersen LF. Urinary excretion of flavonoids reflects even small changes in the dietary intake of fruits and vegetables. *Cancer Epidemiol Biomarkers Prev*. May 2004;13(5):843-9.

173. Lampe JW, Gustafson DR, Hutchins AM, et al. Urinary isoflavonoid and lignan excretion on a Western diet: relation to soy, vegetable, and fruit intake. *Cancer Epidemiol Biomarkers Prev*. Aug 1999;8(8):699-707.

174. Maskarinec G, Singh S, Meng L, Franke AA. Dietary soy intake and urinary isoflavone excretion among women from a multiethnic population. *Cancer Epidemiol Biomarkers Prev*. Jul 1998;7(7):613-9.

175. Frankenfeld CL, Patterson RE, Kalhorn TF, Skor HE, Howald WN, Lampe JW. Validation of a soy food frequency questionnaire with plasma concentrations of isoflavones in US adults. *Journal of the American Dietetic Association*. Oct 2002;102(10):1407-13. doi:10.1016/s0002-8223(02)90313-5

176. Jaceldo-Siegl K, Fraser GE, Chan J, Franke A, Sabaté J. Validation of soy protein estimates from a food-frequency questionnaire with repeated 24-h recalls and isoflavonoid excretion in overnight urine in a Western population with a wide range of soy intakes. *Am J Clin Nutr*. May 2008;87(5):1422-7. doi:10.1093/ajcn/87.5.1422

177. Franke AA, Hebshi SM, Pagano I, Kono N, Mack WJ, Hodis HN. Urine accurately reflects circulating isoflavonoids and ascertains compliance during soy intervention. *Cancer Epidemiol Biomarkers Prev*. Jul 2010;19(7):1775-83. doi:10.1158/1055-9965.Epi-10-0116

178. Franke AA, Morimoto Y, Yeh LM, Maskarinec G. Urinary isoflavonoids as a dietary compliance measure among premenopausal women. *Asia Pacific journal of clinical nutrition*. 2006;15(1):88-94.

179. Frankenfeld CL, Patterson RE, Horner NK, et al. Validation of a soy food-frequency questionnaire and evaluation of correlates of plasma isoflavone concentrations in postmenopausal women. *Am J Clin Nutr*. Mar 2003;77(3):674-80. doi:10.1093/ajcn/77.3.674

180. Kirkman LM, Lampe JW, Campbell DR, Martini MC, Slavin JL. Urinary lignan and isoflavonoid excretion in men and women consuming vegetable and soy diets. *Nutrition and cancer*. 1995;24(1):1-12. doi:10.1080/01635589509514388

181. Frankenfeld CL. O-desmethylangolensin: the importance of equol's lesser known cousin to human health. *Adv Nutr*. Jul 2011;2(4):317-24. doi:10.3945/an.111.000539

182. Tseng M, Olufade T, Kurzer MS, et al. Food frequency questionnaires and overnight urines are valid indicators of daidzein and genistein intake in U.S. women relative to multiple 24-h urine samples. *Nutrition and cancer*. 2008;60(5):619-26. doi:10.1080/01635580801993751

183. Verkasalo PK, Appleby PN, Allen NE, Davey G, Adlercreutz H, Key TJ. Soya intake and plasma concentrations of daidzein and genistein: validity of dietary assessment among eighty British women (Oxford arm of the European Prospective Investigation into Cancer and Nutrition). *Br J Nutr*. Sep 2001;86(3):415-21. doi:10.1079/bjn2001424

184. King RA, Bursill DB. Plasma and urinary kinetics of the isoflavones daidzein and genistein after a single soy meal in humans. *Am J Clin Nutr*. May 1998;67(5):867-72. doi:10.1093/ajcn/67.5.867

185. Kano M, Takayanagi T, Harada K, Sawada S, Ishikawa F. Bioavailability of isoflavones after ingestion of soy beverages in healthy adults. *J Nutr*. Sep 2006;136(9):2291-6. doi:10.1093/jn/136.9.2291

186. Setchell KD, Brown NM, Desai PB, et al. Bioavailability, disposition, and dose-response effects of soy isoflavones when consumed by healthy women at physiologically typical dietary intakes. *J Nutr*. Apr 2003;133(4):1027-35. doi:10.1093/jn/133.4.1027

187. Lampe JW. Isoflavonoid and lignan phytoestrogens as dietary biomarkers. *J Nutr*. Mar 2003;133 Suppl 3:956s-964s. doi:10.1093/jn/133.3.956S

188. Whitton C, Neelakantan N, Ong CN, van Dam RM. Reproducibility of Dietary Biomarkers in a Multiethnic Asian Population. *Mol Nutr Food Res*. Apr 2019;63(8):e1801104. doi:10.1002/mnfr.201801104

189. Sun Q, Bertrand KA, Franke AA, Rosner B, Curhan GC, Willett WC. Reproducibility of urinary biomarkers in multiple 24-h urine samples. *Am J Clin Nutr*. Jan 2017;105(1):159-168. doi:10.3945/ajcn.116.139758

190. Teitelbaum SL, Britton JA, Calafat AM, et al. Temporal variability in urinary concentrations of phthalate metabolites, phytoestrogens and phenols among minority children in the United States. *Environmental research*. Feb 2008;106(2):257-69. doi:10.1016/j.envres.2007.09.010

191. Allred CD, Twaddle NC, Allred KF, et al. Soy processing affects metabolism and disposition of dietary isoflavones in ovariectomized BALB/c mice. *J Agric Food Chem*. Nov 2 2005;53(22):8542-50. doi:10.1021/jf051246w

192. Atkinson W, Downer P, Lever M, Chambers ST, George PM. Effects of orange juice and proline betaine on glycine betaine and homocysteine in healthy male subjects. *Eur J Nutr*. Dec 2007;46(8):446-52. doi:10.1007/s00394-007-0684-5

193. Gibbons H, Michielsen CJR, Rundle M, et al. Demonstration of the utility of biomarkers for dietary intake assessment; proline betaine as an example. *Mol Nutr Food Res*. Oct 2017;61(10)doi:10.1002/mnfr.201700037

194. Erlund I, Meririnne E, Alfthan G, Aro A. Plasma kinetics and urinary excretion of the flavanones naringenin and hesperetin in humans after ingestion of orange juice and grapefruit juice. *J Nutr*. Feb 2001;131(2):235-41. doi:10.1093/jn/131.2.235

195. Nielsen SE, Freese R, Kleemola P, Mutanen M. Flavonoids in human urine as biomarkers for intake of fruits and vegetables. *Cancer Epidemiol Biomarkers Prev*. May 2002;11(5):459-66.

196. Huntley NF, Patience JF. Xylose metabolism in the pig. *PLoS One*. 2018;13(10):e0205913. doi:10.1371/journal.pone.0205913

197. McNamara AE, Collins C, Harsha P, et al. Metabolomic-Based Approach to Identify Biomarkers of Apple Intake. *Mol Nutr Food Res*. Jun 2020;64(11):e1901158. doi:10.1002/mnfr.201901158

198. Vazquez-Manjarrez N, Weinert CH, Ulaszewska MM, et al. Discovery and Validation of Banana Intake Biomarkers Using Untargeted Metabolomics in Human Intervention and Cross-sectional Studies. *J Nutr*. Oct 1 2019;149(10):1701-1713. doi:10.1093/jn/nxz125

199. Lang R, Lang T, Bader M, Beusch A, Schlagbauer V, Hofmann T. High-Throughput Quantitation of Proline Betaine in Foods and Suitability as a Valid Biomarker for Citrus Consumption. *J Agric Food Chem*. Mar 1 2017;65(8):1613-1619. doi:10.1021/acs.jafc.6b05824

200. Harnly JM, Doherty RF, Beecher GR, et al. Flavonoid content of U.S. fruits, vegetables, and nuts. *J Agric Food Chem*. Dec 27 2006;54(26):9966-77. doi:10.1021/jf061478a

201. Escarpa A, González MC. High-performance liquid chromatography with diode-array detection for the determination of phenolic compounds in peel and pulp from different apple varieties. *J Chromatogr A*. Oct 9 1998;823(1-2):331-7. doi:10.1016/s0021-9673(98)00294-5

202. Tsao R, Yang R, Young JC, Zhu H. Polyphenolic profiles in eight apple cultivars using high-performance liquid chromatography (HPLC). *J Agric Food Chem*. Oct 8 2003;51(21):6347-53. doi:10.1021/jf0346298

203. Wingerath T, Stahl W, Sies H. beta-Cryptoxanthin selectively increases in human chylomicrons upon ingestion of tangerine concentrate rich in beta-cryptoxanthin esters. *Arch Biochem Biophys*. Dec 20 1995;324(2):385-90. doi:10.1006/abbi.1995.0052

204. Kanazawa K, Sakakibara H. High content of dopamine, a strong antioxidant, in Cavendish banana. *J Agric Food Chem*. Mar 2000;48(3):844-8. doi:10.1021/jf9909860

205. Edmands WM, Ferrari P, Rothwell JA, et al. Polyphenol metabolome in human urine and its association with intake of polyphenol-rich foods across European countries. *Am J Clin Nutr*. Oct 2015;102(4):905-13. doi:10.3945/ajcn.114.101881

206. Tahiri I, Garro-Aguilar Y, Cayssials V, et al. Urinary flavanone concentrations as biomarkers of dietary flavanone intakes in the European Prospective Investigation into Cancer and Nutrition (EPIC) study. *Br J Nutr*. Mar 28 2020;123(6):691-698. doi:10.1017/S0007114519003131

207. Michaud DS, Giovannucci EL, Ascherio A, et al. Associations of plasma carotenoid concentrations and dietary intake of specific carotenoids in samples of two prospective cohort studies using a new carotenoid database. *Cancer Epidemiol Biomarkers Prev*. Apr 1998;7(4):283-90.

208. Ferrari P, Al-Delaimy WK, Slimani N, et al. An approach to estimate between- and within-group correlation coefficients in multicenter studies: plasma carotenoids as biomarkers of intake of fruits and vegetables. *Am J Epidemiol*. Sep 15 2005;162(6):591-8. doi:10.1093/aje/kwi242

209. Pujos-Guillot E, Hubert J, Martin JF, et al. Mass spectrometry-based metabolomics for the discovery of biomarkers of fruit and vegetable intake: citrus fruit as a case study. *J Proteome Res*. Apr 5 2013;12(4):1645-59. doi:10.1021/pr300997c

210. Playdon MC, Sampson JN, Cross AJ, et al. Comparing metabolite profiles of habitual diet in serum and urine. *The American Journal of Clinical Nutrition*. 2016;104(3):776-789. doi:10.3945/ajcn.116.135301

211. Koeth RA, Wang Z, Levison BS, et al. Intestinal microbiota metabolism of l-carnitine, a nutrient in red meat, promotes atherosclerosis. *Nature Medicine*. 2013/05/01 2013;19(5):576-585. doi:10.1038/nm.3145

212. Etxeberria U, Arias N, Boqué N, et al. Shifts in microbiota species and fermentation products in a dietary model enriched in fat and sucrose. *Benef Microbes*. Mar 2015;6(1):97-111. doi:10.3920/bm2013.0097

213. Romano KA, Vivas EI, Amador-Noguez D, Rey FE. Intestinal microbiota composition modulates choline bioavailability from diet and accumulation of the proatherogenic metabolite trimethylamine-N-oxide. *mBio*. Mar 17 2015;6(2):e02481. doi:10.1128/mBio.02481-14

214. Beckmann M, Joosen AM, Clarke MM, et al. Changes in the human plasma and urinary metabolome associated with acute dietary exposure to sucrose and the identification of potential biomarkers of sucrose intake. *Molecular Nutrition & Food Research*. 2016;60(2):444-457. doi:<https://doi.org/10.1002/mnfr.201500495>

215. Valenzuela LO, O'Grady SP, Enright LE, Murtaugh M, Sweeney C, Ehleringer JR. Evaluation of childhood nutrition by dietary survey and stable isotope analyses of hair and breath. *Am J Hum Biol*. May 2018;30(3):e23103. doi:10.1002/ajhb.23103

216. Yun HY, Tinker LF, Neuhouser ML, et al. The Carbon Isotope Ratios of Serum Amino Acids in Combination with Participant Characteristics can be Used to Estimate Added Sugar Intake in a Controlled Feeding Study of US Postmenopausal Women. *J Nutr*. Oct 12 2020;150(10):2764-2771. doi:10.1093/jn/nxaa195

217. Choy K, Nash SH, Kristal AR, Hopkins S, Boyer BB, O'Brien DM. The Carbon Isotope Ratio of Alanine in Red Blood Cells Is a New Candidate Biomarker of Sugar-Sweetened Beverage Intake. *The Journal of Nutrition*. 2013;143(6):878-884. doi:10.3945/jn.112.172999

218. Moore LB, Liu SV, Halliday TM, Neilson AP, Hedrick VE, Davy BM. Urinary Excretion of Sodium, Nitrogen, and Sugar Amounts Are Valid Biomarkers of Dietary Sodium, Protein, and High Sugar Intake in Nonobese Adolescents. *The Journal of Nutrition*. 2017;147(12):2364-2373. doi:10.3945/jn.117.256875

219. Kuhnle GG, Joosen AM, Wood TR, Runswick SA, Griffin JL, Bingham SA. Detection and quantification of sucrose as dietary biomarker using gas chromatography and liquid chromatography with mass spectrometry. *Rapid Commun Mass Spectrom*. 2008;22(3):279-82. doi:10.1002/rcm.3355

220. Choy K, Nash SH, Kristal AR, Hopkins S, Boyer BB, O'Brien DM. The carbon isotope ratio of alanine in red blood cells is a new candidate biomarker of sugar-sweetened beverage intake. *J Nutr*. Jun 2013;143(6):878-84. doi:10.3945/jn.112.172999

221. Gibbons H, McNulty BA, Nugent AP, et al. A metabolomics approach to the identification of biomarkers of sugar-sweetened beverage intake. *Am J Clin Nutr*. Mar 2015;101(3):471-7. doi:10.3945/ajcn.114.095604

222. Abreu TC, Hulshof PJM, Boshuizen HC, Trijsburg L, Gray N, de Vries JHM. Validity Coefficient of Repeated Measurements of Urinary Marker of Sugar Intake Is Comparable to Urinary Nitrogen as Marker of Protein Intake in Free-living Subjects. *Cancer Epidemiol Biomarkers Prev*. Jan 2021;30(1):193-202. doi:10.1158/1055-9965.Epi-20-0271

223. MacDougall CR, Hill CE, Jahren AH, et al. The δ13C Value of Fingerstick Blood Is a Valid, Reliable, and Sensitive Biomarker of Sugar-Sweetened Beverage Intake in Children and Adolescents. *The Journal of Nutrition*. 2018;148(1):147-152. doi:10.1093/jn/nxx017

224. Hedrick VE, Zoellner JM, Jahren AH, Woodford NA, Bostic JN, Davy BM. A Dual-Carbon-and-Nitrogen Stable Isotope Ratio Model Is Not Superior to a Single-Carbon Stable Isotope Ratio Model for Predicting Added Sugar Intake in Southwest Virginian Adults. *The Journal of Nutrition*. 2015;145(6):1362-1369. doi:10.3945/jn.115.211011

225. Intemann T, Pigeot I, De Henauw S, et al. Urinary sucrose and fructose to validate self-reported sugar intake in children and adolescents: results from the I.Family study. *Eur J Nutr*. Apr 2019;58(3):1247-1258. doi:10.1007/s00394-018-1649-6

226. Bingham S, Luben R, Welch A, Tasevska N, Wareham N, Khaw KT. Epidemiologic Assessment of Sugars Consumption Using Biomarkers: Comparisons of Obese and Nonobese Individuals in the European Prospective Investigation of Cancer Norfolk. *Cancer Epidemiology, Biomarkers & Prevention*. 2007;16(8):1651-1654. doi:10.1158/1055-9965.Epi-06-1050

227. Le MT, Frye RF, Rivard CJ, et al. Effects of high-fructose corn syrup and sucrose on the pharmacokinetics of fructose and acute metabolic and hemodynamic responses in healthy subjects. *Metabolism*. 2012;61(5):641-651.

228. Votruba SB, Shaw PA, Oh EJ, et al. Associations of plasma, RBCs, and hair carbon and nitrogen isotope ratios with fish, meat, and sugar-sweetened beverage intake in a 12-wk inpatient feeding study. *The American Journal of Clinical Nutrition*. 2019;110(6):1306-1315. doi:10.1093/ajcn/nqz208

229. Tasevska N, Runswick SA, McTaggart A, Bingham SA. Urinary sucrose and fructose as biomarkers for sugar consumption. *Cancer Epidemiol Biomarkers Prev*. May 2005;14(5):1287-94. doi:10.1158/1055-9965.Epi-04-0827

230. Abreu TC, Hulshof PJM, Boshuizen HC, Trijsburg L, Gray N, de Vries JHM. Validity Coefficient of Repeated Measurements of Urinary Marker of Sugar Intake Is Comparable to Urinary Nitrogen as Marker of Protein Intake in Free-living Subjects. *Cancer Epidemiology, Biomarkers & Prevention*. 2021;30(1):193-202. doi:10.1158/1055-9965.Epi-20-0271

231. Wang JS, Luo H, Wang P, et al. Validation of green tea polyphenol biomarkers in a phase II human intervention trial. *Food Chem Toxicol*. Jan 2008;46(1):232-40. doi:10.1016/j.fct.2007.08.007

232. Sun CL, Yuan JM, Koh WP, Yu MC. Green tea, black tea and breast cancer risk: a meta-analysis of epidemiological studies. *Carcinogenesis*. Jul 2006;27(7):1310-5. doi:10.1093/carcin/bgi276

233. Hodgson JM, Chan SY, Puddey IB, et al. Phenolic acid metabolites as biomarkers for tea- and coffee-derived polyphenol exposure in human subjects. *Br J Nutr*. Feb 2004;91(2):301-6. doi:10.1079/bjn20031046

234. Yuan JM, Gao YT, Yang CS, Yu MC. Urinary biomarkers of tea polyphenols and risk of colorectal cancer in the Shanghai Cohort Study. *Int J Cancer*. Mar 15 2007;120(6):1344-50. doi:10.1002/ijc.22460

235. Manach C, Williamson G, Morand C, Scalbert A, Remesy C. Bioavailability and bioefficacy of polyphenols in humans. I. Review of 97 bioavailability studies. *Am J Clin Nutr*. Jan 2005;81(1 Suppl):230S-242S. doi:10.1093/ajcn/81.1.230S

236. Neveu V, Perez-Jiménez J, Vos F, et al. Phenol-Explorer: an online comprehensive database on polyphenol contents in foods. *Database*. January 1, 2010 2010;2010:bap024. doi:10.1093/database/bap024

237. USDA. Composition of Foods Raw, Processed, Prepared - USDA National Nutrient Database for Standard Reference, Release 22. 2009:<http://www.ars.usda.gov/Services/docs.htm?docid=18879> (accessed January 3, 2009).

238. Guertin KA, Loftfield E, Boca SM, et al. Serum biomarkers of habitual coffee consumption may provide insight into the mechanism underlying the association between coffee consumption and colorectal cancer. *The American Journal of Clinical Nutrition*. March 11, 2015 2015;101:1000-11. doi:10.3945/ajcn.114.096099

239. Rothwell JA, Keski-Rahkonen P, Robinot N, et al. A metabolomic study of biomarkers of habitual coffee intake in four European countries. *Molec Nutr Food Res*. 2019;63(22):e1900659.

240. Rothwell JA, Loftfield E, Wedekind R, et al. A Metabolomic Study of the Variability of the Chemical Composition of Commonly Consumed Coffee Brews. *Metabolites*. 2019;9(1):17.

241. Shi L, Brunius C, Johansson I, et al. Plasma metabolite biomarkers of boiled and filtered coffee intake and their association with type 2 diabetes risk. *Journal of internal medicine*. Dec 9 2019;doi:10.1111/joim.13009

242. de Oliveira Otto MC, Nettleton JA, Lemaitre RN, et al. Biomarkers of dairy fatty acids and risk of cardiovascular disease in the Multi-ethnic Study of Atherosclerosis. *Journal of the American Heart Association*. Jul 18 2013;2(4):e000092. doi:10.1161/jaha.113.000092

243. Lucas M, Proust F, Blanchet C, et al. Is marine mammal fat or fish intake most strongly associated with omega-3 blood levels among the Nunavik Inuit? *Prostaglandins, leukotrienes, and essential fatty acids*. Sep 2010;83(3):143-50. doi:10.1016/j.plefa.2010.06.006

244. Medina-Remón A, Barrionuevo-González A, Zamora-Ros R, et al. Rapid Folin-Ciocalteu method using microtiter 96-well plate cartridges for solid phase extraction to assess urinary total phenolic compounds, as a biomarker of total polyphenols intake. *Analytica chimica acta*. Feb 16 2009;634(1):54-60. doi:10.1016/j.aca.2008.12.012
